# Facile N9-Alkylation of Xanthine Derivatives and Their Use as Precursors for *N*-Heterocyclic Carbene Complexes

**DOI:** 10.3390/molecules26123705

**Published:** 2021-06-17

**Authors:** Moloud Mokfi, Jörg Rust, Christian W. Lehmann, Fabian Mohr

**Affiliations:** 1 Faculty of Mathematics and Natural Sciences, Inorganic Chemistry, Bergische University Wuppertal, 42119 Wuppertal, Germany; moloud.mokfi@yahoo.com; 2 Chemical Crystallography and Electron Microscopy Centre, Max Planck Institute for Coal Research, 45470 Mülheim an der Ruhr, Germany; rust@mpi-muelheim.mpg.de (J.R.); lehmann@mpi-muelheim.mpg.de (C.W.L.)

**Keywords:** xanthines, *N*-heterocyclic carbenes, X-ray structures

## Abstract

The xanthine-derivatives 1,3,7-trimethylxanthine, 1,3-dimethyl-7-benzylxanthine and 1,3-dimethyl-7-(4-chlorobenzyl)xanthine are readily ethylated at N9 using the cheap alkylating agents ethyl tosylate or diethyl sulfate. The resulting xanthinium tosylate or ethyl sulfate salts can be converted into the corresponding PF_6_^−^ and chloride salts. The reaction of these xanthinium salts with silver(I) oxide results in the formation of different silver(I) carbene-complexes. In the presence of ammonia, ammine complexes [Ag(NHC)(NH_3_)]PF_6_ are formed, whilst with Et_2_NH, the bis(carbene) salts [Ag(NHC)_2_]PF_6_ were isolated. Using the xanthinium chloride salts neutral silver(I) carbenes [Ag(NHC)Cl] were prepared. These silver complexes were used in a variety of transmetallation reactions to give the corresponding gold(I), ruthenium(II) as well as rhodium(I) and rhodium(III) complexes. The compounds were characterized by various spectroscopic methods as well as X-ray diffraction.

## 1. Introduction

Metal complexes containing *N*-heterocyclic carbenes (NHCs) are today considered a common class of ligands in organometallic chemistry. Their seemingly endless structural variety combined with a very stable metal-carbon-bond makes carbene complexes so important in homogeneous catalysis and also for biomedical applications [1,2,3,4,5,6,7,8,9,10,11,12,13,14]. Typically, most *N*-heterocyclic carbenes are derived from substituted imidazolium, benzimidazolium or triazolium salts. Related to this class of compounds are the xanthinium salts, which may also be used as precursors for *N*-heterocyclic carbenes. Especially xanthinium salts derived from caffeine or theophylline have been studied to some extent. The first reported xanthine-derived NHC complex was the mercury(II) bis(NHC) salt [Hg(NHC)_2_]ClO_4_ (NHC = 1,3,7,9-tetramethylxanthin-8-ylidene), prepared by Beck in 1976 [15]. After lying dormant for more than a quarter of a century, the groups of Youngs and Herrmann independently began to reinvestigate NHC-complexes derived from caffeine and its derivatives with rhodium(I), iridium(I) and silver(I) [16,17,18]. Especially the silver(I) complexes developed and patented by Youngs were found to be highly active against various pathogens [19,20,21,22,23,24]. Other groups have examined xanthine-derived carbene complexes with metals including Pt(II) [25,26,27], Pd(II) [28,29,30,31,32], Au(I) [33], Ir(I) [34] and Ag(I) [35,36]. Focus of these studies was their activity against cancer cells (Pt, Pd, Au, Ir and Ag) or homogeneous catalysis (Pd, Rh). There is also a publication on copper complexes containing a caffeine-derived NHC, but the results must be considered doubtful [37]. The compounds are referred to as being blue copper(II) species, but an alleged X-ray structure (data neither shown in the publication nor deposited with the CCDC) and NMR spectroscopic data are clearly consistent with the presence of copper(I). A short review from 2018 summarizes the so far known chemistry of xanthine-based *N*-heterocyclic carbenes [38]. Given our interest in NHC-complexes of various metals and their biological activity and reactivity [39,40,41,42,43,44], we wished to develop more efficient methods to access a greater variety of xanthinium salts as well as their corresponding metal carbene-complexes.

## 2. Results and Discussion

Quaternization at the 9-position of xanthines including caffeine and its derivatives typically requires forcing conditions (high temperature and pressure), long reaction times and a large excess of alkyl halide. In the case of *N*9-methylation of xanthines, typically, 20 equivalents of methyl iodide are used and reactions are carried out in refluxing DMF or in a closed vessel for up to 20 h [19,28,35,45]. Trimethyloxonium tetrafluoroborate has also be used by some groups for *N*-methylation of xanthines [17,33]. Although reaction conditions are mild and no large excess is required, [Me_3_O]BF_4_ is considerably more expensive than MeI and requires anhydrous conditions. As an alternative to these reagents, methyl tosylate or dimethyl sulfate have been successfully used for *N*9-methylation in xanthines, affording the respective xanthinium tosylate or methyl sulfate salts in high yields and short reaction times (90 min at 170 °C without additional solvent) [16,46,47,48]. Based on this procedure, we examined the feasibility of also introducing ethyl groups in position 9 of different xanthine derivatives with ethyl tosylate or diethyl sulfate.

**Scheme 1:**
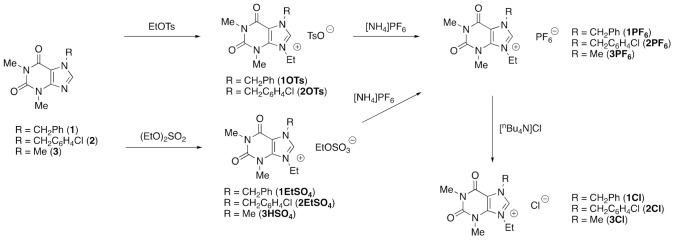
Synthesis of xanthinium salts.

Gratifyingly, heating 1,3-dimethyl-7-benzylxanthine in neat ethyl tosylate at 150 °C cleanly affords the corresponding 1,3-dimethyl-7-benzyl-9-ethylxanthinium tosylate in two hours with an isolated yield of 87% (Scheme 1).

Work-up merely involves washing the product with diethyl ether and drying. This result encouraged us to try the even cheaper diethyl sulfate. Indeed, reacting 1,3-dimethyl-7-benzylxanthine in neat diethyl sulfate at 130 °C for two hours afforded the desired product as the ethyl sulfate salt in 87% yield (Scheme 1). Using the same procedure, the corresponding 4-chlorobenzyl- and caffeine-derivatives were also isolated as colorless solids in high yields (Scheme 1). In the case of caffeine, the ethyl sulfate anion evidently hydrolyzes during the reaction, forming the hydrogen sulfate salt instead. These xanthinium salts were characterized by NMR spectroscopy and electrospray mass spectrometry, the latter (in positive ion mode) shows only one signal corresponding to the respective xanthinium cations and, in negative ion mode, only the signals for the anions (EtSO_4_^−^, TsO^−^ or PF_6_^−^). The positive-ion mass spectra of the caffeine derivatives also feature one signal, which can be assigned to the fragment [*M-Me+H*]^+^, formed by loss of one methyl group.

The tosylate, ethyl sulfate or hydrogen sulfate anions in these salts could readily be exchanged in water to give the corresponding PF_6_^−^ salts **1PF_6_**, **2PF_6_** and **3PF_6_** (Scheme 1). Since halide salts are however the most convenient precursors for *N*-heterocyclic carbene metal complexes, we investigated the possibility of converting the PF_6_^−^ salts into the corresponding chlorides. The group of Visentin reported that a xanthinium tetrafluoroborate could be converted into the chloride salt by anion exchange with [Ph_4_As]Cl [31]. Given the toxicity and high cost of [Ph_4_As]Cl, we sought alternative reagents to accomplish this anion exchange. Gratifyingly, we found that mixing THF solutions of the xanthinium PF_6_^−^ salts with [^n^Bu_4_N]Cl results in precipitation of the chloride salts **1Cl**, **2Cl** and **3Cl**, which could be isolated almost quantitatively (Scheme 1). Based on mass spectrometry, the samples are free of PF_6_^−^ anions and the presence of a chloride anion was confirmed by a sharp singlet in their ^35^Cl-NMR spectra. Unfortunately, it was not possible to access the chloride salts directly from the ethyl sulfate of hydrogen sulfate salts.

**Scheme 2:**
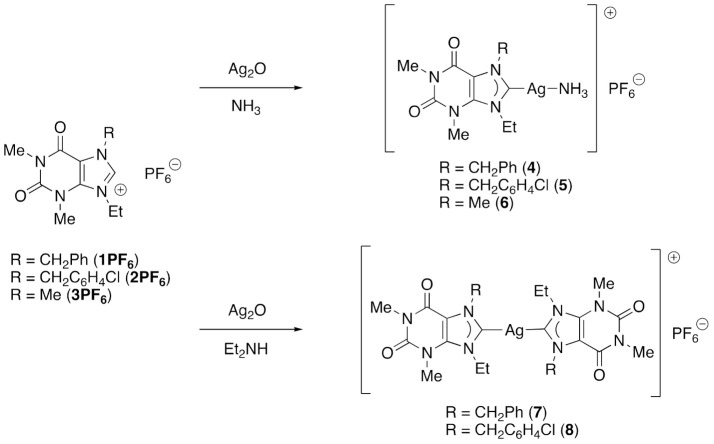
Synthesis of silver-NHC complexes.

With these xanthinium salts in hand, we examined their use as precursors for *N*-heterocyclic carbene complexes of silver. The reaction of the respective PF_6_^−^ salts with Ag_2_O in a mixture of ethanol and aqueous ammonia afforded the corresponding cationic silver(I) carbene complexes [Ag(NHC)(NH_3_)]PF_6_, (NHC = 1,3-dimethyl-7-benzyl-9-ethylxanthine-8-ylidene (**4**), 1,3-dimethyl-7-(4-chlorobenzyl)-9-ethylxanthine-8-ylidene (**5**) and 1,3,7-trimethyl-9-ethylxanthine-8-ylidene (**6**)) containing ammonia as co-ligand (Scheme 2) [49].

**Figure 1:**
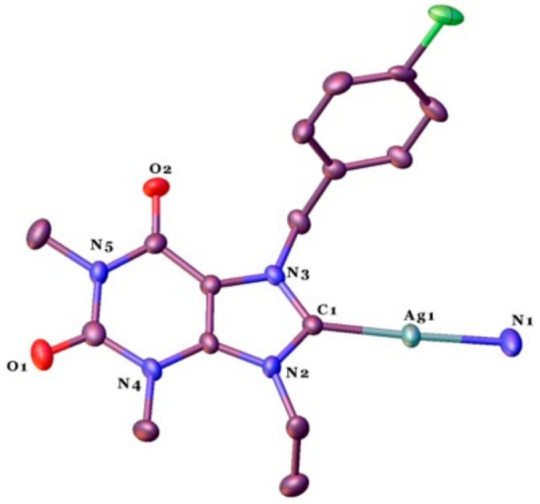
Molecular structure of compound **5**. Thermal ellipsoids are drawn at the 50% level. Hydrogen atoms and the PF_6_^−^ anion have been omitted for clarity.

**Figure 2:**
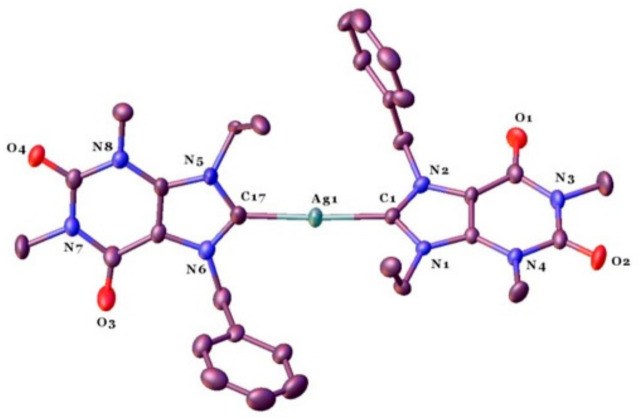
Molecular structure of compound **7**. Thermal ellipsoids are drawn at the 50% level. Hydrogen atoms and the PF_6_^−^ anion have been omitted for clarity.

When using Et_2_NH instead of ammonia, we isolated the bis(carbene) silver(I) salts [Ag(NHC)_2_]PF_6_ (NHC = 1,3-dimethyl-7-benzyl-9-ethylxanthine-8-ylidene (**7**) and 1,3-dimethyl-7-(4-chlorobenzyl)-9-ethylxanthine-8-ylidene (**8**)) (Scheme 2). The silver carbene compounds were characterized by NMR spectroscopy, mass spectrometry and single crystal X-ray diffraction. The proton spectra of the compounds lack the signal for the proton at position 8, consistent with carbene formation. This is further confirmed by a significant shift of the resonance of the carbon atom at position 8. In the xanthinium salts, these are observed between 138 and 139 ppm, whilst in the silver carbene complexes they fall in the range of 185 to 187 ppm. The chemical shifts of metal-bound carbene-carbon resonances can often vary, depending on the other ligands bound to the metal. In this series of silver compounds, the nature of the second ligand (NH_3_, carbene or Cl^−^) does not appear to have a significant effect on the chemical shift of the carbene-carbon resonance. In the case of the bis(carbene) complexes **7** and **8**, coupling between the ^13^C and the ^107/109^Ag isotopes with coupling constants of 192 and 217 Hz could be observed. These values are typical for silver(I) NHC-complexes, which have been observed in several other bis(carbene) complexes [50]. In the case of the ammonia complexes **4**–**6**, broad signals due to the ammine can be observed in their proton NMR spectra at about 3 ppm. Furthermore, bands between 3300 and 3400 cm^−1^ due to the NH_3_ stretching frequency are seen in their IR spectra. The molecular structures of compounds **5** and **7** are shown in Figure 1 and Figure 2.

In each case, the coordination about the silver is, as expected, linear with angles of 176.28° and 177.29°, respectively. The carbon-silver bond lengths (ca. 2.06 Å) are typical for silver(I)-NHC complexes. Although Ag-carbene complexes with coordinated ammonia were postulated as intermediates and one example was isolated and characterized spectroscopically [49], complex **5** is the only structurally authenticated example of a silver(I) carbene with ammonia co-ligands to date.

**Scheme 3:**
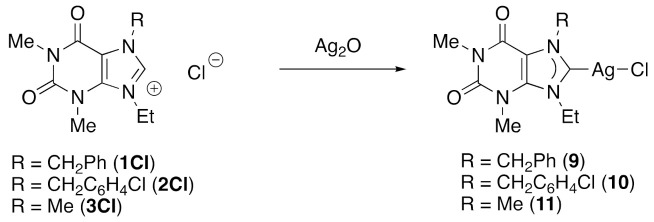
Synthesis of silver-chloride complexes.

The chloride salts **1Cl**, **2Cl** and **3Cl** readily react with Ag_2_O in CH_2_Cl_2_ to form the silver chloride complexes [Ag(NHC)Cl] (NHC = 1,3-dimethyl-7-benzyl-9-ethylxanthine-8-ylidene (**9**), 1,3-dimethyl-7-(4-chlorobenzyl)-9-ethylxanthine-8-ylidene (**10**) and 1,3,7-trimethyl-9-ethylxanthine-8-ylidene (**11**)) as colorless solids in reasonable yields (Scheme 3).

**Figure 3:**
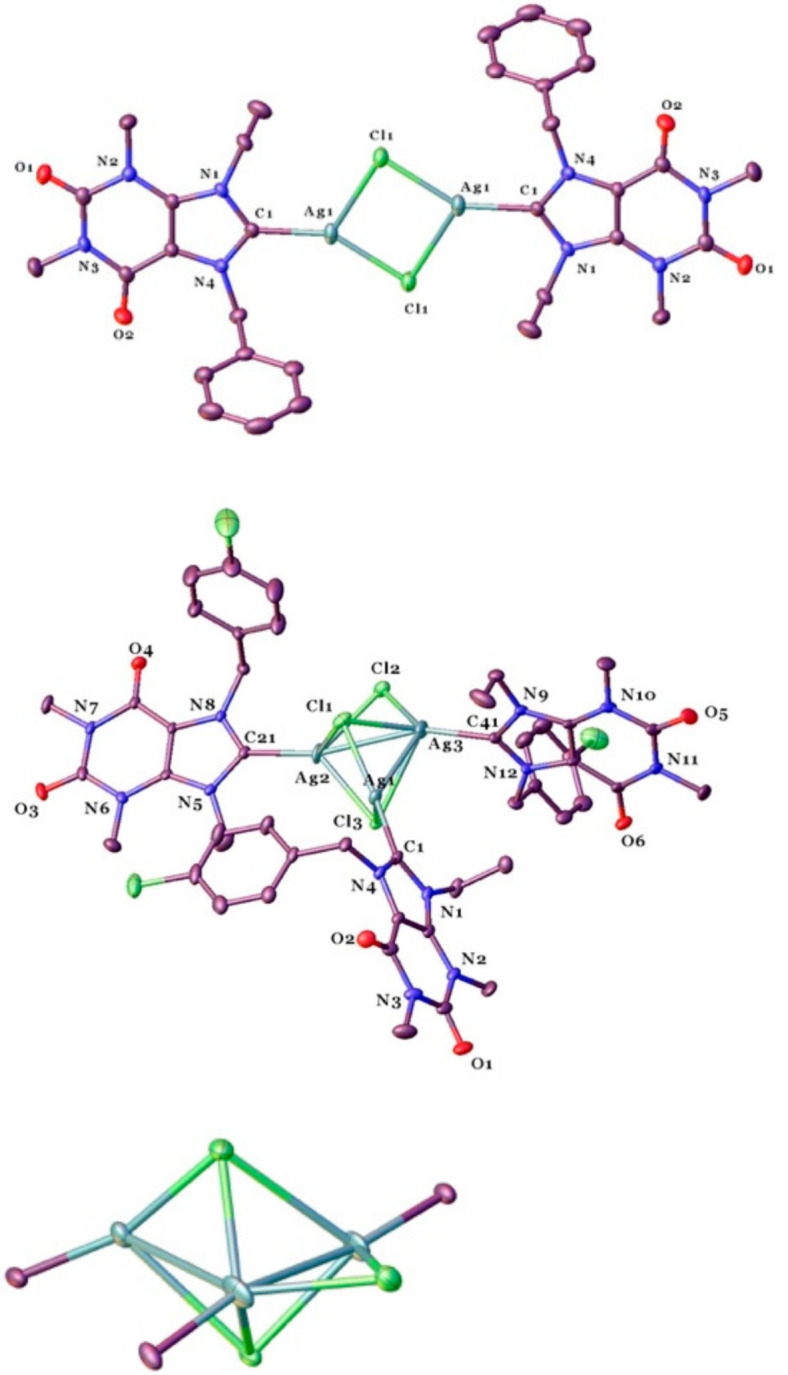
Molecular structures of compounds **9** (top) and **10** (center and bottom). Thermal ellipsoids are drawn at the 50% level. Hydrogen atoms have been omitted for clarity. Atoms labeled with an asterisk are generated by symmetry. The bottom image shows only the atoms directly attached to Ag in complex **10**.

The formation of the carbene complex is again evident from the NMR spectroscopic data. In compounds **9** and **10**, the resonance for the carbene-carbon atom is observed at about 187 ppm in their ^13^C NMR spectra. In the solid-state, the molecules exist as either a chloro-bridged dimer with tri-coordinated silver atoms (**9**) or a unique trimer (**10**) as depicted in Figure 3.

For compound **9** the two Ag-Cl bond lengths of 2.44 Å and 2.72 Å are unequal, resulting in an Ag_2_Cl_2_ parallelogram. This structural motif is observed in several other silver NHC complexes [51,52]. Crystals of complex **10** were very small and thin, therefore diffraction data was collected at beamline P11 at the PETRA III synchrotron located at DESY in Hamburg, Germany. The trimeric structure of **10** is unique and, so far, has no precedence in the literature. The molecule consists of a planar Ag_3_-triangle with two shorter (3.11 and 3.21 Å) one much longer (3.89 Å) Ag-Ag distances. Above and below this Ag_3_-plane there is a μ_3_-bridging chloride ligand. The third chloride acts as μ_2_-ligand between two silver atoms in the plane of the triangle. Each silver atom is also *C*-bound to the carbene ligand (Figure 3 bottom). As can be seen, the C-Ag-Cl angles are not linear, resulting in a distorted tetrahedral coordination environment about each silver atom.

**Scheme 4:**
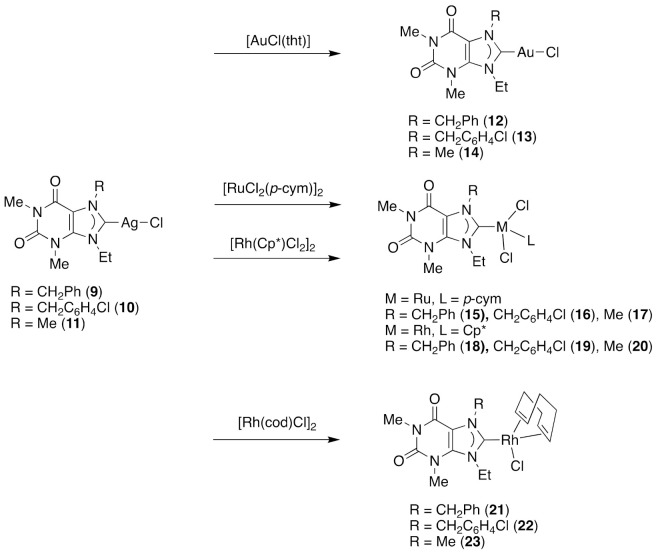
Transmetallation reactions.

Silver NHC complexes are commonly used in transmetallation reactions to transfer the NHC ligand to a different metal. We therefore examined the reaction of the silver chloride complexes **9**–**11** with various other metal salts including [AuCl(tht)] (tht = tetrahydrothiophene), [Ru(*p*-cym)Cl_2_]_2_, [Rh(Cp*)Cl_2_]_2_ and [Rh(cod)Cl]_2_. In each case the corresponding metal xanthine-8-ylidene-derivatives were formed in good yields as air- and moisture-stable solids (Scheme 4).

**Figure 4:**
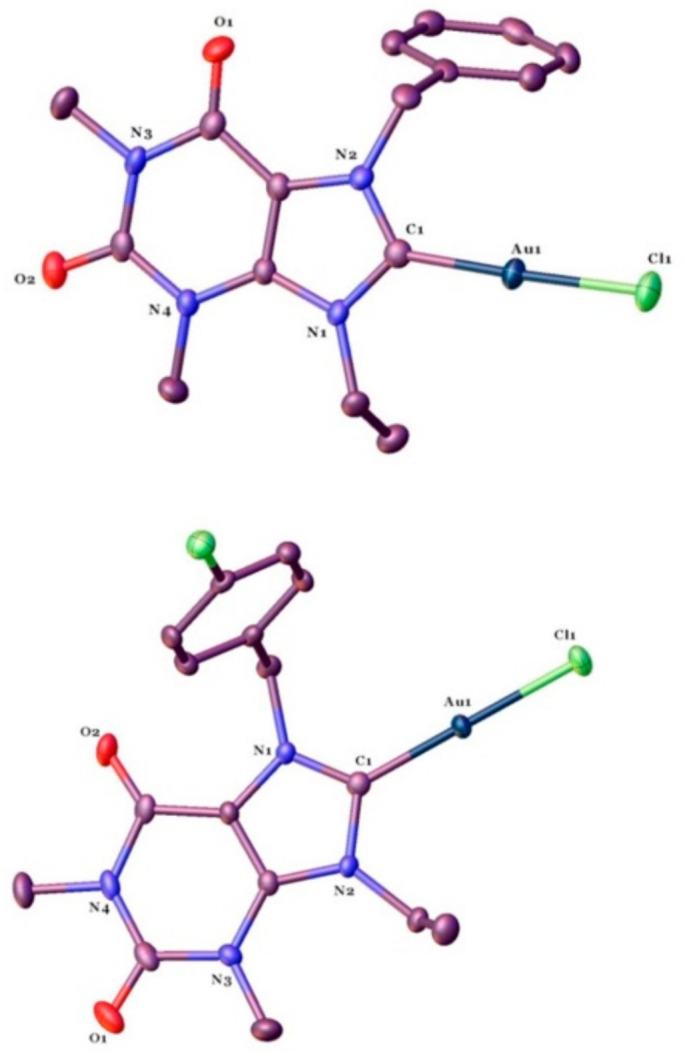

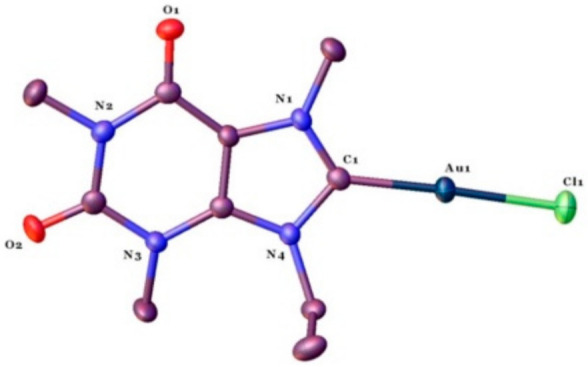
Molecular structures of compounds **12** (top), **13** (middle) and **14** (bottom). Thermal ellipsoids are drawn at the 50% level. Hydrogen atoms have been omitted for clarity.

**Figure 5:**
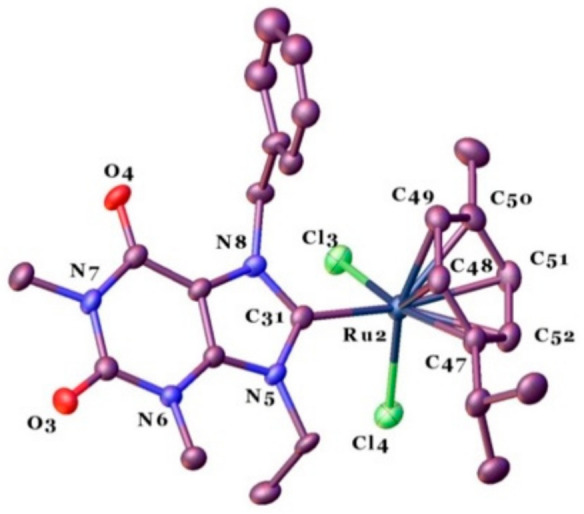
Molecular structure of compound **15**. Thermal ellipsoids are drawn at the 50% level. Hydrogen atoms have been omitted for clarity.

**Figure 6:**
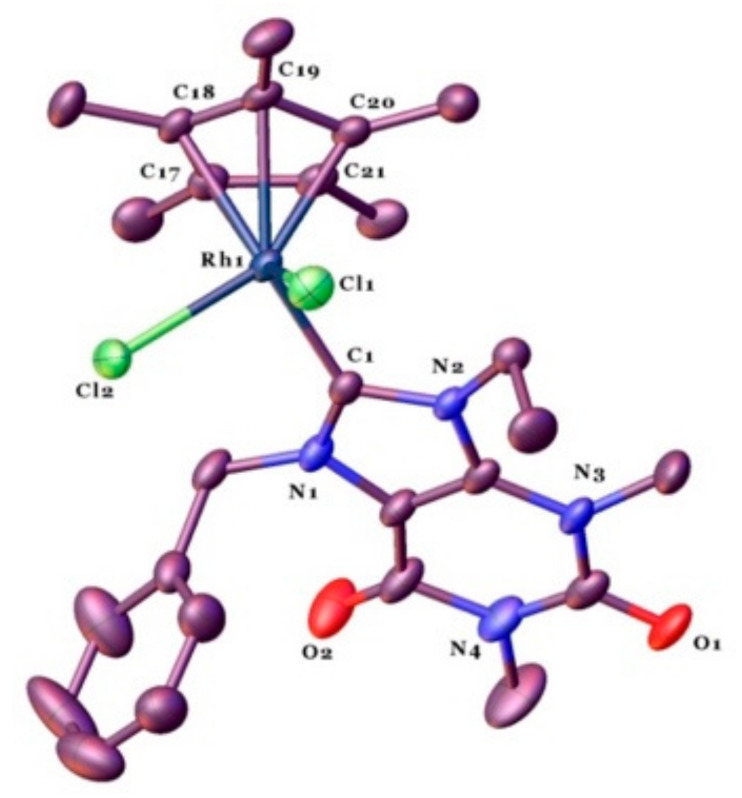

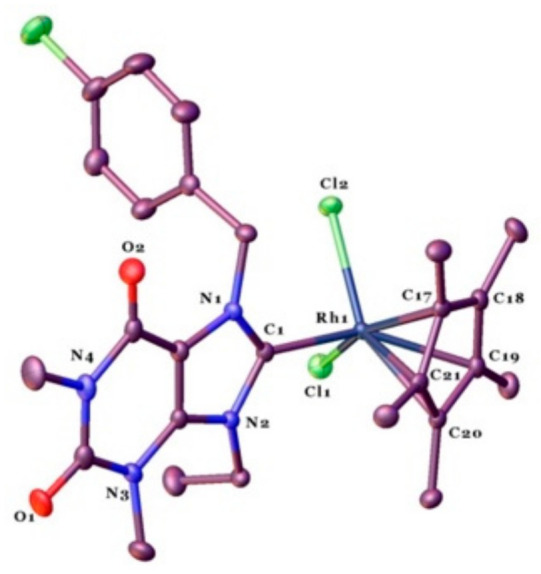
Molecular structures of compounds **18** (top) and **19** (bottom). Thermal ellipsoids are drawn at the 50% level. Hydrogen atoms have been omitted for clarity.

**Figure 7:**
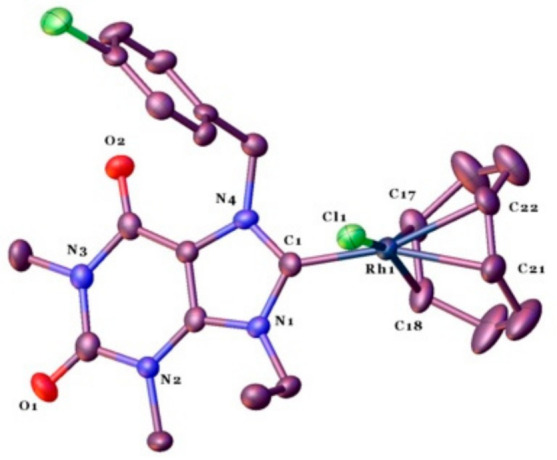
Molecular structure of compound **22**. Thermal ellipsoids are drawn at the 50% level. Hydrogen atoms have been omitted for clarity.

Complexes **12**–**23** were characterized by various spectroscopic methods and, in several cases, by X-ray diffraction. In all these compounds, the chemical shifts of the carbene-carbon resonances in the ^13^C-NMR spectra are most diagnostic. Compared to the silver-precursors, the resonances are shifted either slightly upfield or downfield, depending on the metal. In case of the Rh-complexes, coupling between the ^13^C and ^103^Rh nuclei can be observed. Several complexes were characterized by X-ray diffraction, the molecular structures of which are shown in Figure 4, Figure 5, Figure 6 and Figure 7.

The gold(I) complexes (Figure 4) feature linear coordination (C-Au-Cl angles of 177–179°) at the gold, as would be expected. There is only one other reported X-ray structure of a xanthine-derived NHC Au(I) halide complex, namely that of the iodo-complex [Au(NHC)I] (NHC = 1,3,7,9-tetramethylxanthine-8-ylidene) [33]. The Au-C bond lengths in **12**–**13** are with around 1.9 Å, similar to those observed in the 1,3,7,9-tetramethylxanthine-8-ylidene derivative.

The structure of the ruthenium(II) complex **15** (Figure 5) features one *C*-bound carbene and two chloride ligands at the arene-Ru center. This piano-stool type arrangement is typical for this class of compounds. While the preparation and biological properties of the 1,3,7,9-tetramethylxanthine-8-ylidene-analogue of **15** have been published [53], this is the first X-ray structure of an arene ruthenium complex containing a xanthine-derived carbene ligand.

The Rh(III) complexes **18** and **19** (Figure 6) feature a similar piano-stool geometry, with the xanthine-derivative *C8*-bound to the metal. The only other structurally characterized examples of Cp*Rh(III) complexes with xanthine-derived carbene ligands are those reported by Hahn containing 7-picolyl- or 7-imidazoly-substituted theobromine-derivatives [54].

The structure of complex **22** (Figure 7) contains a *cis*-configured, square planar rhodium(I) center with a *C8*-bound xanthine-derivative. The overall geometry is similar to that reported for the 1,3,7,9-tetramethylxanthine-8-ylidene-analogue [RhI(cod)(NHC)] [18].

## 3. Materials and Methods

Reactions were routinely carried out under ambient conditions without protection from air or moisture. Solvents were HPLC-grade and all other chemicals were procured from commercial suppliers (Merck, Darmstadt, Germany, Alfa Aesar, Kandel, Germany, TCI Chemicals, Eschborn, Germany or Acros Organics, Geel, Belgium) and were used as received. 1,3-Dimethyl-7-benzylxanthine was prepared from theophylline and benzyl bromide as described previously [55,56]. The 4-chlorobenzyl-derivative was prepared similarly. [AuCl(tht)], [RuCl_2_(*p*-cym)]_2_, [Rh(Cp*)Cl_2_]_2_ and [Rh(cod)Cl]_2_ were prepared by kwon methods [57,58,59,60]. NMR spectra were recorded on Bruker Avance 400 or Bruker Avance III 600 instruments (Bruker, Berlin, Germany). Spectra were referenced externally to Me_4_Si (^1^H, ^13^C), 1 M aq. NaCl (^35^Cl) or 85% H_3_PO_4_ (^31^P). High-resolution electrospray mass spectra were measured on a Bruker Daltonics micrOTOF instrument (Bruker, Bremen, Germany). IR spectra were recorded on a Nicolet iS5 spectrometer (Thermo Fisher Scientific, Erlangen, Germany) fitted with a diamond iD7 ATR unit.

**1,3-Dimethyl-7-benzyl-9-ethylxanthinium tosylate (1OTs)**

A mixture of 1,3-dimethyl-7-benzylxanthine (**1**) (0.100 g, 0.370 mmol) and ethyl tosylate (0.267 g, 1.35 mmol) was heated in an open vial to 150 °C for 2 h. After cooling, excess diethyl ether was added and the resulting solid was isolated by filtration, washed with Et_2_O and subsequently dried in air. As a result, 0.151 g (87%) of a colorless solid was obtained. ^1^H NMR (400 MHz, DMSO-d_6_) δ = 9.63 (s, 1 H, C^8^H), 7.34–7.49 (m, 7 H, OTs, Ph), 7.10 (d, *J* = 8.4 Hz, 2 H, OTs), 5.71 (s, 2 H, N^7^CH_2_), 4.59 (q, *J* = 7.2 Hz, 2 H, N^9^CH_2_), 3.71 (s, 3 H, N^3^Me), 3.26 (s, 3 H, N^1^Me), 2.29 (s, 3 H, Me OTs), 1.54 (t, *J* = 7.2 Hz, 3 H, Me). ^13^C{^1^H} NMR (100 MHz, DMSO-d_6_) δ = 153.06 (C^6^=O), 150.27 (C^2^=O), 145.68 (OTs), 139.51 (C^4^), 138.51 (C^8^), 137.55 (OTs), 134.14 (Ph), 128.78, (Ph), 128.67 (Ph), 128.04 (Ph), 127.98 (OTs), 125.40 (OTs), 107.09 (C^5^), 51.21 (N^7^CH_2_), 45.32 (N^9^CH_2_), 31.66 (N^3^Me), 28.39 (N^1^Me), 20.72 (Me OTs), 15.03 (Me). High-resolution positive-ion ESMS (*m*/*z*): calculated for [*M*]^+^ 299.1508; found 299.1511.

**1,3-Dimethyl-7-(4-chlorobenzyl)-9-ethylxanthinium tosylate (2OTs)**

This was prepared as described above using 1,3-dimethyl-7-(4-chlorobenzyl)xanthine (**2**) (0.100 g, 0.330 mmol) and ethyl tosylate (0.238 g, 1.19 mmol). As a result, 0.139 g (84%) of a colorless solid was obtained. ^1^H NMR (400 MHz, DMSO-d_6_) δ = 9.60 (s, 1 H, C^8^H), 7.44–7.49 (m, 6 H, OTs, Bn), 7.09–7.12 (m, 2 H, OTs), 5.68 (s, 2 H, N^7^CH_2_), 4.58 (q, *J* = 7.2 Hz, 2 H, N^9^CH_2_), 3.71 (s, 3 H, N^3^Me), 3.25 (s, 3 H, N^1^Me), 2.29 (s, 3 H, Me OTs), 1.53 (t, *J* = 7.2 Hz, 3 H, Me). ^13^C{^1^H} NMR (100 MHz, DMSO-d_6_) δ = 153.02 (C^6^=O), 150.25 (C^2^=O), 145.68 (OTs), 139.51 (C^4^), 138.61 (C^8^), 137.54 (OTs), 133.43 (Bn), 133.05 (Bn), 130.09 (Bn), 128.72 (Bn), 127.97 (OTs), 125.39 (OTs), 107.06 (C^5^), 50.57 (N^7^CH_2_), 45.34 (N^9^CH_2_), 31.66 (N^3^Me), 28.39 (N^1^Me), 20.71 (Me OTs), 14.96 (Me). High-resolution positive-ion ESMS (*m*/*z*): calculated for [*M*]^+^ 333.1118; found 333.1118.

**1,3-Dimethyl-7-benzyl-9-ethylxanthinium ethyl sulfate (1EtSO_4_)**

This was prepared as described above by heating a mixture of 1,3-dimethyl-7-benzylxanthine (**1**) (0.20 g, 0.74 mmol) and diethyl sulfate (194 μL, 1.48 mmol) to 130 °C for 2 h. As a result, 0.273 g (87%) of a colorless solid was obtained. ^1^H NMR (600 MHz, CDCl_3_) δ = 9.99 (s, 1 H, C^8^H), 7.34–7.66 (m, 5 H, Ph), 5.80 (s, 2 H, N^7^CH_2_), 4.76 (q, *J* = 7.2 Hz, 2 H, N^9^CH_2_), 3.06 (q, *J* = 7.1 Hz, 2 H, OCH_2_), 3.85 (s, 3 H, N^3^Me), 3.43 (s, 3 H, N^1^Me), 1.68 (t, *J* = 7.2 Hz, 3 H, Me), 1.27 (t, *J* = 7.1 Hz, 3 H, OCH_2_Me). ^13^C{^1^H} NMR (150 MHz, DMSO-d_6_) δ = 153.08 (C^6^=O), 150.29 (C^2^=O), 139.55 (C^4^), 138.54 (C^8^), 134.15 (Ph), 128.77 (Ph), 128.66 (Ph), 128.03 (Ph), 107.11 (C^5^), 61.07 (OCH_2_), 51.20 (N^7^CH_2_), 45.31 (N^9^CH_2_), 31.67 (N^3^Me), 28.39 (N^1^Me), 15.06 (Me, OCH_2_Me). High-resolution positive-ion ESMS (*m*/*z*): calculated for [*M*]^+^ 299.1508; found 299.1503.

**1,3-Dimethyl-7-(4-chlorobenzyl)-9-ethylxanthinium ethyl sulfate (2EtSO_4_)**

This was prepared as described above using 1,3-dimethyl-7-(4-chlorobenzyl)xanthine (**2**) (0.200 g, 0.650 mmol) and ethyl tosylate (172 μL, 1.31 mmol). As a result, 0.264 g (88%) of a colorless solid was obtained. ^1^H NMR (600 MHz, CDCl_3_) δ = 10.06 (s, 1 H, C^8^H), 7.33–7.62 (m, 4 H, Bn), 5.78 (s, 2 H, N^7^CH_2_), 4.74 (q, *J* = 7.3 Hz, 2 H, N^9^CH_2_), 4.03 (q, *J* = 7.1 Hz, 2 H, OCH_2_), 3.85 (s, 3 H, N^3^Me), 3.43 (s, 3 H, N^1^Me), 1.68 (t, *J* = 7.3 Hz, 3 H, Me), 1.27 (t, *J* = 7.1 Hz, 3 H, OCH_2_Me). ^13^C{^1^H} NMR (100 MHz, DMSO-d_6_) δ = 153.06 (C^6^=O), 150.29 (C^2^=O), 139.56 (C^4^), 138.61 (C^8^), 133.44 (Bn), 133.05 (Bn), 130.09 (Bn), 128.73 (Bn), 107.10 (C^5^), 61.09 (OCH_2_), 50.58 (N^7^CH_2_), 45.34 (N^9^CH_2_), 31.68 (N^3^Me), 28.40 (N^1^Me), 15.07 (Me), 14.99 (OCH_2_Me). High-resolution positive-ion ESMS (*m*/*z*): calculated for [*M*]^+^ 333.1118; found 333.1122.

**1,3,7-Trimethyl-9-ethylxanthinium hydrogen sulfate (3HSO_4_)**

A mixture of caffeine (**3**) (0.287 g, 1.480 mmol) and diethyl sulfate (388 μL, 2.96 mmol) was heated in an open vial to 130 °C for 2 h. After cooling to room temperature, a 3:7 mixture of acetone and toluene (ca. 10 mL) was added. Upon standing over night, a colorless solid deposited, which was isolated by filtration and was washed with a small amount of acetone. After drying in vacuum, 0.435 g (91%) of the product was obtained. ^1^H NMR (600 MHz, DMSO-d_6_) δ = 9.42 (s, 1 H, C^8^H), 4.58 (q, *J* = 7.2 Hz, 2 H, N^9^CH_2_), 4.07 (s, 3 H, N^7^Me), 3.73 (s, 3 H, N^3^Me), 3.28 (s, 3 H, N^1^Me), 1.53 (t, *J* = 7.2 Hz, 3 H, Me). ^13^C{^1^H} NMR (100 MHz, DMSO-d_6_) δ = 153.31 (C^6^=O), 150.34 (C^2^=O), 138.87 (C^4^), 138.76 (C^8^), 107.87 (C^5^), 44.97 (N^9^CH_2_), 35.61 (N^7^Me), 31.58 (N^3^Me), 28.31 (N^1^Me), 15.06 (Me). High-resolution positive-ion ESMS (*m*/*z*): calculated for [*M-Me+H*]^+^ 209.1039; found 209.1035.

**1,3-Dimethyl-7-benzyl-9-ethylxanthinium hexafluorophosphate (1PF_6_)**

A solution of **1EtSO_4_** (0.100 g, 0.236 mmol) in water (10 mL) was treated with a solution of [NH_4_]PF_6_ (0.0403 g, 0.247 mmol) in water (10 mL). After 20 min at room temperature the resulting precipitate was isolated by filtration, washed with water and Et_2_O and was dried in air. A colorless solid was obtained in 74% yield (0.077 g). The compound can also be prepared by the same method from **1OTs** in similar yield. ^1^H NMR (400 MHz, DMSO-d_6_) δ = 9.60 (s, 1 H, C^8^H), 7.32–7.49 (m, 5 H, Ph), 5.71 (s, 2 H, N^7^CH_2_), 4.59 (q, *J* = 7.2 Hz, 2 H, N^9^CH_2_), 3.72 (s, 3 H, N^3^Me), 3.27 (s, 3 H, N^1^Me), 1.54 (t, *J* = 7.2 Hz, 3 H, Me). ^13^C{^1^H} NMR (100 MHz, DMSO-d_6_) δ = 153.07 (C^6^=O), 150.28 (C^2^=O), 139.51 (C^4^), 138.48 (C^8^), 134.11 (Ph), 128.78 (Ph), 128.68 (Ph), 128.00 (Ph), 107.11 (C^5^), 51.22 (N^7^CH_2_), 45.30 (N^9^CH_2_), 31.66 (N^3^Me), 28.39 (N^1^Me), 15.07 (Me). ^31^P NMR (DMSO-d_6_) δ = −144.19 (hept., *J* = 711 Hz, PF_6_). High-resolution positive-ion ESMS (*m*/*z*): calculated for [*M*]^+^ 299.1508; found 299.1511.

**1,3-Dimethyl-7-(4-chlorobenzyl)-9-ethylxanthinium hexafluorophosphate (2PF_6_)**

This was prepared as described above using **2EtSO_4_** (0.100 g, 0.218 mmol) and [NH_4_]PF_6_ (0.0373 g, 0.229 mmol). As a result, 0.077 g (74%) of a colorless solid was obtained. The compound can also be prepared by the same method from **2OTs** in similar yield. ^1^H NMR (400 MHz, DMSO-d_6_) δ = 9.57 (s, 1 H, C^8^H), 7.44–7.54 (m, 4 H, Bn), 5.70 (s, 2 H, N^7^CH_2_), 4.59 (q, *J* = 7.2 Hz, 2 H, N^9^CH_2_), 3.72 (s, 3 H, N^3^Me), 3.26 (s, 3 H, N^1^Me), 1.54 (t, *J* = 7.2 Hz, 3 H, Me). ^13^C{^1^H} NMR (100 MHz, DMSO-d_6_) δ = 153.04 (C^6^=O), 150.27 (C^2^=O), 139.52 (C^4^), 138.58 (C^8^), 133.46 (Bn), 133.01 (Bn), 130.07 (Bn), 128.74 (Bn), 107.09 (C^5^), 50.59 (N^7^CH_2_), 45.32 (N^9^CH_2_), 31.67 (N^3^Me), 28.40 (N^1^Me), 15.00 (Me). ^31^P NMR (DMSO-d_6_) δ = −144.19 (hept., *J* = 711 Hz, PF_6_). High-resolution positive-ion ESMS (*m*/*z*): calculated for [*M*]^+^ 333.1118; found 333.1118.

**1,3,7-Trimethyl-9-ethylxanthinium hexafluorophosphate (3PF_6_)**

This was prepared as described above using **3HSO_4_** (0.433 g, 1.412 mmol) and [NH_4_]PF_6_ (0.440 g, 2.70 mmol). As a result, 0.382 g (77%) of a colorless solid was obtained. ^1^H NMR (400 MHz, DMSO-d_6_) δ = 9.34 (s, 1 H, C^8^H), 4.56 (q, *J* = 7.2 Hz, 2 H, N^9^CH_2_), 4.05 (d, *J* = 0.6 Hz, 3 H, N^7^Me), 3.71 (s, 3 H, N^3^Me), 3.27 (s, 3 H, N^1^Me), 1.50 (t, *J* = 7.2 Hz, 3 H, Me). ^13^C{^1^H} NMR (100 MHz, DMSO-d_6_) δ = 153.31 (C^6^=O), 150.33 (C^2^=O), 138.85 (C^4^), 138.69 (C^8^), 107.88 (C^5^), 44.98 (N^9^CH_2_), 35.63 (N^7^Me), 31.56 (N^3^Me), 28.32 (N^1^Me), 15.09 (Me). ^31^P NMR (DMSO-d_6_) δ = -144.21 (hept., *J* = 711 Hz, PF_6_). High-resolution positive-ion ESMS (*m*/*z*): calculated for [*M-Me+H*]^+^ 209.1039; found 209.1035.

**1,3-Dimethyl-7-benzyl-9-ethylxanthinium chloride (1Cl)**

A solution of **1PF_6_** (0.2416 g, 0.544 mmol) in THF (3 mL) was treated with a solution of [^n^Bu_4_N]Cl (0.304 g, 1.094 mmol) in THF (2.5 mL). After 2 h at room temperature the resulting precipitate was isolated by filtration, washed with Et_2_O and dried. A colorless solid was obtained in 85% yield (0.1548 g). ^1^H NMR (400 MHz, DMSO-d_6_) δ = 10.01 (s, 1 H, C^8^H), 7.32–7.54 (m, 5 H, Ph), 5.74 (s, 2 H, N^7^CH_2_), 4.62 (q, *J* = 7.2 Hz, 2 H, N^9^CH_2_), 3.72 (s, 3 H, N^3^Me), 3.26 (s, 3 H, N^1^Me), 1.55 (t, *J* = 7.2 Hz, 3 H, Me). ^13^C{^1^H} NMR (100 MHz, DMSO-d_6_) δ = 153.08 (C^6^=O), 150.29 (C^2^=O), 139.53 (C^4^), 138.71 (C^8^), 134.22 (Ph), 128.74 (Ph), 128.64 (Ph), 128.09 (Ph), 107.03 (C^5^), 51.10 (N^7^CH_2_), 45.30 (N^9^CH_2_), 31.69 (N^3^Me), 28.39 (N^1^Me), 15.09 (Me). ^35^Cl NMR (54 MHz, DMSO-d_6_) δ = 69.4 (s, Cl^−^). High-resolution positive-ion ESMS (*m*/*z*): calculated for [*M*]^+^ 299.1508; found 299.1503.

**1,3-Dimethyl-7-(4-chlorobenzyl)-9-ethylxanthinium chloride (2Cl)**

This was prepared as described above using **2PF_6_** (0.2604 g, 0.544 mmol) and [^n^Bu_4_N]Cl (0.304 g, 1.094 mmol). As a result, 0.152 g (75%) colorless solid was obtained. ^1^H NMR (400 MHz, DMSO-d_6_) δ = 10.01 (s, 1 H, C^8^H), 7.47–7.55 (m, 4 H, Bn), 5.73 (s, 2 H, N^7^CH_2_), 4.61 (q, *J* = 7.2 Hz, 2 H, N^9^CH_2_), 3.72 (s, 3 H, N^3^Me), 3.26 (s, 3 H, N^1^Me), 1.55 (t, *J* = 7.2 Hz, 3 H, Me). ^13^C{^1^H} NMR (100 MHz, DMSO-d_6_) δ = 153.05 (C^6^=O), 150.27 (C^2^=O), 139.53 (C^4^), 138.81 (C^8^), 133.42 (Bn), 133.14 (Bn), 130.17 (Bn), 128.69 (Bn), 107.00 (C^5^), 50.44 (N^7^CH_2_), 45.33 (N^9^CH_2_), 31.70 (N^3^Me), 28.39 (N^1^Me), 15.01(Me). ^35^Cl NMR (54 MHz, DMSO-d_6_) δ = 69.4 (s, Cl^−^). High-resolution positive-ion ESMS (*m*/*z*): calculated for [*M*]^+^ 333.1118; found 333.1122.

**1,3,7-Trimethyl-9-ethylxanthinium chloride (3Cl)**

This was prepared as described above using **3PF_6_** (0.200 g, 0.543 mmol) and [^n^Bu_4_N]Cl (0.304 g, 1.094 mmol). As a result, 0.136 g (97%) of a colorless solid was obtained. ^1^H NMR (400 MHz, DMSO-d_6_) δ = 9.58 (s, 1 H, C^8^H), 4.58 (q, *J* = 7.2 Hz, 2 H, N^9^CH_2_), 4.07 (d, *J* = 0.6 Hz, 3 H, N^7^Me), 3.72 (s, 3 H, N^3^Me), 3.28 (s, 3 H, N^1^Me), 1.51 (t, *J* = 7.2 Hz, 3 H, Me). ^13^C{^1^H} NMR (150 MHz, DMSO-d_6_) δ = 153.32 (C^6^=O), 150.34 (C^2^=O), 138.92 (C^4^), 138.85 (C^8^), 107.82 (C^5^), 44.97 (N^9^CH_2_), 35.59 (N^7^Me), 31.61 (N^3^Me), 28.31 (N^1^Me), 15.12 (Me). ^35^Cl NMR (39 MHz, DMSO-d_6_) δ = 38.50 (s, Cl^−^). High-resolution positive-ion ESMS (*m*/*z*): calculated for [*M*]^+^ 223.1195; found 223.1191.

**[(NHC)Ag(NH_3_)]PF_6_ (4)**

A suspension of **1PF_6_** (0.111 g, 0.250 mmol) in EtOH (3 mL) was treated with Ag_2_O (0.029 g, 0.125 mmol) and concentrated ammonia solution (170 μL, 2.6 mmol). After stirring at room temperature for 30 min, the colorless solid was isolated by filtration and was washed with cold EtOH and Et_2_O. A colorless solid was obtained in 78% yield (0.110 g). ^1^H NMR (400 MHz, DMSO-d_6_) δ = 7.25–7.40 (m, 5 H, Ph), 5.72 (s, 2 H, N^7^CH_2_), 4.59 (m, 2 H, N^9^CH_2_), 3.72 (s, 3 H, N^3^Me), 3.50 (s, 3 H, NH_3_), 3.24 (s, 3 H, N^1^Me), 1.42 (m, 3 H, Me). ^13^C{^1^H} NMR (150 MHz, DMSO-d_6_) δ = 185.27 (C-Ag), 152.93 (C^6^=O), 150.57 (C^2^=O), 140.26 (C^4^), 136.56 (Ph), 128.58 (Ph), 127.88 (Ph), 127.27 (Ph), 108.28 (C^5^), 52.86 (N^7^CH_2_), 46.32 (N^9^CH_2_), 31.35 (N^3^Me), 28.15 (N^1^Me), 17.21 (Me). ^31^P NMR (DMSO-d_6_) δ = −144.19 (hept., *J* = 711 Hz, PF_6_). High-resolution positive-ion ESMS (*m*/*z*): calculated for [*M+H+Na*]^+^ 446.0722; found 446.0686. IR (ATR): 3300–3400 cm^−1^ ν(NH_3_), 833 cm^−1^ ν(PF_6_).

**[(NHC)Ag(NH_3_)]PF_6_ (5)**

This was prepared as described above using **2PF_6_** (0.119 g, 0.250 mmol), Ag_2_O (0.029 g, 0.125 mmol) and concentrated ammonia solution (170 μL, 2.6 mmol). As a result, 0.116 g (77%) of a colorless solid was obtained. ^1^H NMR (400 MHz, DMSO-d_6_) δ = 7.28–7.45 (m, 4 H, Bn), 5.71 (s, 2 H, N^7^CH_2_), 4.60 (m, 2 H, N^9^CH_2_), 3.72 (s, 3 H, N^3^Me), 3.23 (s, 3 H, N^1^Me), 3.03 (s, 3 H, NH_3_), 1.43 (m, 3 H, Me). ^13^C{^1^H} NMR (100 MHz, DMSO-d_6_) δ = 197.15 (C-Ag), 152.92 (C^6^=O), 150.60 (C^2^=O), 140.34 (C^4^), 135.63 (Bn), 132.60 (Bn), 129.10 (Bn), 128.56 (Bn), 108.22 (C^5^), 52.02 (N^7^CH_2_), 46.35 (N^9^CH_2_), 31.39 (N^3^Me), 28.20 (N^1^Me), 17.26 (Me). ^31^P NMR (DMSO-d_6_) δ = −144.19 (hept., *J* = 711 Hz, PF_6_). High-resolution positive-ion ESMS (*m*/*z*): calculated for [*M+H+Na*]^+^ 480.0332; found 480.0328. IR (ATR): 3300–3400 cm^−1^ ν(NH_3_), 833 cm^−1^ ν(PF_6_). X-ray quality crystals were obtained by slow diffusion of CHCl_3_ into an acetone solution of the compound.

**[(NHC)Ag(NH_3_)]PF_6_ (6)**

This was prepared as described above using **3PF_6_** (0.092 g, 0.250 mmol), Ag_2_O (0.029 g, 0.125 mmol) and concentrated ammonia solution (163 μL, 2.7 mmol). As a result, 0.084 g (68%) of a colorless solid was obtained. H NMR (600 MHz, DMSO-d_6_) δ = 4.60 (q, *J* = 7.0 Hz, 2 H, N^9^CH_2_), 4.08 (s, 3 H, N^7^Me), 3.72 (s, 3 H, N^3^Me), 3.26 (s, 3 H, N^1^Me), 3.06 (br. s, 3 H, NH_3_), 1.46 (t, *J* = 7.0 Hz, 3 H, Me). ^13^C{^1^H} NMR (150 MHz, DMSO-d_6_) δ = 153.21 (C^6^=O), 150.68 (C^2^=O), 139.83 (C^4^), 108.88 (C^5^), 46.04 (N^9^CH_2_), 38.03 (N^7^Me), 31.34 (N^3^Me), 28.17 (N^1^Me), 17.38 (Me). The carbene-carbon signal could not be detected. ^31^P NMR (DMSO-d_6_) δ = −144.19 (hept., *J* = 711 Hz, PF_6_). High-resolution positive-ion ESMS (*m*/*z*): calculated for [*M*]^+^ 347.0511; found 347.0505. IR (ATR): 3300–3400 cm^−1^ ν(NH_3_), 833 cm^−1^ ν(PF_6_).

**[Ag(NHC)_2_]PF_6_ (7)**

A suspension of **1PF_6_** (0.0555 g, 0.125 mmol) in absolute EtOH (2 mL) was treated with Ag_2_O (0.0145 g, 0.0626 mmol) and Et_2_NH (65 μL, 0.627 mmol). After stirring at room temperature for 4 h, the colorless solid was isolated by filtration and was washed with cold EtOH and Et_2_O. A colorless solid was obtained in 80% yield (0.0425 g). ^1^H NMR (600 MHz, DMSO-d_6_) δ = 7.25–7.33 (m, 10 H, Ph), 5.74 (s, 4 H, N^7^CH_2_), 4.56 (q, *J* = 7.2 Hz, 4 H, N^9^CH_2_), 3.73 (s, 6 H, N^3^Me), 3.25 (s, 6 H, N^1^Me), 1.39 (t, *J* = 7.2 Hz, 6 H, Me). ^13^C{^1^H} NMR (150 MHz, DMSO-d_6_) δ = 186.24 (d, *J* = 217 Hz, C^109^Ag), 186.22 (d, *J* = 192 Hz, C^107^Ag), 152.98 (C^6^=O), 150.61 (C^2^=O), 140.23 (C^8^), 136.59 (Ph), 128.61 (Ph), 127.88 (Ph), 127.00 (Ph), 108.34 (C^5^), 52.69 (N^7^CH_2_), 46.35 (N^9^CH_2_), 31.38 (N^3^Me), 28.19 (N^1^Me), 17.26 (Me). ^31^P NMR (acetone-d_6_) δ = −144.27 (hept., *J* = 707 Hz, PF_6_). High-resolution positive-ion ESMS (*m*/*z*): calculated for [*M*]^+^ 703.1910; found 703.1906. X-ray quality crystals were obtained by slow diffusion of Et_2_O into an acetone solution of the compound.

**[Ag(NHC)_2_]PF_6_ (8)**

This was prepared as described above using **2PF_6_** (0.0598 g, 0.125 mmol), Ag_2_O (0.0145 g, 0.0626 mmol) and Et_2_NH (65 μL, 0.627 mmol). As a result, 0.040 g (72%) of a colorless solid was obtained. ^1^H NMR (400 MHz, Acetone-d6) δ = 7.30–7.43 (m, 8 H, Bn), 5.83 (s, 4 H, N^7^CH_2_), 4.79 (q, J = 7.3 Hz, 4 H, N^9^CH_2_), 3.88 (s, 6 H, N^3^Me), 3.30 (s, 6 H, N^1^Me), 1.60 (t, J = 7.3 Hz, 6 H, Me). ^13^C{^1^H} NMR (100 MHz, Acetone-d6) δ = 187.42 (unres. d, C-Ag), 154.96 (C^6^=O), 152.47 (C^2^=O), 142.06 (C^4^), 137.19 (Bn), 135.15 (Bn), 130.77 (Bn), 130.41 (Bn), 110.44 (C^5^), 54.25 (N^7^CH_2_), 48.63 (N^9^CH_2_), 32.77 (N^3^Me), 29.36 (N^1^Me), 18.76 (Me). ^31^P NMR (acetone-d6) δ = −144.27 (hept., *J* = 707 Hz, PF_6_). High-resolution positive-ion ESMS (*m*/*z*): calculated for [*M*]^+^ 771.1131; found 771.1130.

**[Ag(NHC)Cl] (9)**

A solution of **1Cl** (0.05 g, 0.149 mmol) in CH_2_Cl_2_ (7.5 mL) was treated with Ag_2_O (0.0174 g, 0.0751 mmol). After 4 h at room temperature most of the silver oxide had dissolved. The mixture was passed through Celite, and the filtrate was concentrated in vacuum. Addition of Et_2_O precipitated a colorless solid, which was isolated by filtration and was washed with Et_2_O. A colorless solid was obtained in 67% yield (0.0445 g). ^1^H NMR (400 MHz, DMSO-d_6_) δ = 7.26–7.39 (m, 5 H, Ph), 5.70 (s, 2 H, N^7^CH_2_), 4.58 (q, *J* = 7.2 Hz, 2 H, N^9^CH_2_), 3.71 (s, 3 H, N^3^Me), 3.23 (s, 3 H, N^1^Me), 1.44 (t, *J* = 7.2 Hz, 3 H, Me). ^13^C{^1^H} NMR (100 MHz, DMSO-d_6_) δ = 186.77 (C-Ag), 152.97 (C^6^=O), 150.59 (C^2^=O), 140.24 (C^4^), 136.56 (Ph), 128.60 (Ph), 127.93 (Ph), 127.38 (Ph), 108.15 (C^5^), 52.88 (N^7^CH_2_), 46.41 (N^9^CH_2_), 31.38 (N^3^Me), 28.20 (N^1^Me), 17.24 (Me). High-resolution positive-ion ESMS (*m*/*z*): calculated for [*M+Na*]^+^ 463.0067; found 463.077. X-ray quality crystals were obtained by slow diffusion of Et_2_O into a CH_2_Cl_2_ solution of the compound.

**[Ag(NHC)Cl] (10)**

This was prepared as described above using **2Cl** (0.0554 g, 0.140 mmol) and Ag_2_O (0.0174 g, 0.0751 mmol). As a result, 0.0475 g (66%) of a colorless solid was obtained. ^1^H NMR (400 MHz, Acetone-d_6_) δ = 7.34–7.58 (m, 4 H, Bn), 5.81 (s, 2 H, N^7^CH_2_), 4.78 (q, *J* = 7.2 Hz, 2 H, N^9^CH_2_), 3.89 (s, 3 H, N^3^Me), 3.31 (s, 3 H, N^1^Me), 1.59 (t, *J* = 7.2 Hz, 3 H, Me). ^13^C{^1^H} NMR (100 MHz, Acetone-d_6_) δ = 187.60 (C-Ag), 154.87 (C^6^=O), 152.35 (C^2^=O), 142.24 (C^4^), 137.22 (Bn), 135.10 (Bn), 131.48 (Bn), 130.23 (Bn), 110.12 (C^5^), 54.39 (N^7^CH_2_), 48.48 (N^9^CH_2_), 32.78 (N^3^Me), 29.36 (N^1^Me), 18.61 (Me). High-resolution positive-ion ESMS (*m*/*z*): calculated for [*M+Na*]^+^ 496.9677; found 496.9671. X-ray quality crystals were obtained by slow diffusion of Et_2_O into a CH_2_Cl_2_ solution of the compound.

**[Ag(NHC)Cl] (11)**

This was prepared as described above using **3Cl** (0.0387 g, 0.150 mmol) and Ag_2_O (0.0174 g, 0.0751 mmol). As a result, 0.0285 g (52%) of a colorless solid was obtained. ^1^H NMR (400 MHz, DMSO-d_6_) δ = 4.56 (q, *J* = 7.2 Hz, 2 H, N^9^CH_2_), 4.04 (s, 3 H, N^7^Me), 3.70 (s, 3 H, N^3^Me), 3.24 (s, 3 H, N^1^Me), 1.44 (t, *J* = 7.2 Hz, 3 H, Me). ^13^C{^1^H} NMR (100 MHz, DMSO-d_6_) δ = 185.25 (C-Ag), 153.18 (C^6^=O), 150.66 (C^2^=O), 139.80 (C^4^), 108.79 (C^5^), 46.09 (N^9^CH_2_), 37.99 (N^7^Me), 31.33 (N^3^Me), 28.14 (N^1^Me), 17.30 (Me). High-resolution positive-ion ESMS (*m*/*z*): calculated for [*M-AgCl*]^+^ 223.1195; found 223.1218.

**[Au(NHC)Cl] (12)**

A solution of complex **9** (0.0442 g, 0.100 mmol) and [AuCl(tht)] (0.0320 g, 0.0998 mmol) in CH_2_Cl_2_ (5 mL) was left to stir at room temperature for ca. 4 h. The mixture was passed through Celite, and the solvent was removed under reduced pressure. The resulting material was washed with Et_2_O and dried in air. A colorless product was obtained in 64% yield (0.034 g). ^1^H NMR (600 MHz, CDCl_3_) δ = 7.62 (d, *J* = 6.7 Hz, 2 H, *o*-Ph), 7.27–7.36 (m, 3 H, *m*-Ph, *p*-Ph), 5.78 (s, 2 H, N^7^CH_2_), 4.70 (q, *J* = 7.2 Hz, 2 H, N^9^CH_2_), 3.79 (s, 3 H, N^3^Me), 3.38 (s, 3 H, N^1^Me), 1.58 (t, *J* = 7.2 Hz, 3 H, Me). ^13^C{^1^H} NMR (150 MHz, CDCl_3_) δ = 176.79 (C-Au), 152.93 (C^6^=O), 150.63 (C^2^=O), 139.33 (C^4^), 134.87 (*ipso*-Ph), 128.84, 128.73, 128.59 (Ph), 108.16 (C^5^), 53.68 (N^7^CH_2_), 46.71 (N^9^CH_2_), 31.68 (N^3^Me), 28.84 (N^1^Me), 17.33 (Me). High-resolution positive-ion ESMS (*m*/*z*): calculated for [*M+Na*]^+^ 553.0682; found 553.0792.

**[Au(NHC)Cl] (13)**

This was prepared as described above using **10** (0.0476 g, 0.100 mmol) and [AuCl(tht)] (0.0320 g, 0.0998 mmol). As a result, 0.0420 g (74%) colorless solid was obtained. ^1^H NMR (600 MHz, CDCl_3_) δ = 7.58 (d, *J* = 8.4 Hz, 2 H, *o*-C_6_H_4_), 7.28–7.31 (m, 2 H, *m*-C_6_H_4_), 5.74 (s, 2 H, N^7^CH_2_), 4.69 (q, *J* = 7.2 Hz, 2 H, N^9^CH_2_), 3.79 (s, 3 H, N^3^Me), 3.38 (s, 3 H, N^1^Me), 1.58 (t, *J* = 7.2 Hz, 3 H, Me). ^13^C{^1^H} NMR (150 MHz, CDCl_3_) δ = 176.79 (C-Au), 152.96 (C^6^=O), 150.57 (C^2^=O), 139.40 (C^4^), 134.87 (*ipso*-Bn), 133.29 (Bn), 130.10 (Bn), 129.04 (Bn), 108.04 (C^5^), 52.96 (N^7^CH_2_), 46.77 (N^9^CH_2_), 31.70 (N^3^Me), 28.86 (N^1^Me), 17.33 (Me). High-resolution positive-ion ESMS (*m*/*z*): calculated for [*M+Na*]^+^ 587.0292; found 587.0291.

**[Au(NHC)Cl] (14)**

This was prepared as described above using **11** (0.0365 g, 0.988 mmol) and [AuCl(tht)] (0.0320 g, 0.0998 mmol). As a result, 0.0295 g (55%) colorless solid was obtained. ^1^H NMR (400 MHz, CDCl_3_) δ = 4.71 (q, *J* = 7.2 Hz, 2 H, N^9^CH_2_), 4.17 (s, 3 H, N^7^Me), 3.83 (s, 3 H, N^3^Me), 3.43 (s, 3 H, N^1^Me), 1.60 (t, *J* = 7.2 Hz, 3 H, Me). ^13^C{^1^H} NMR (100 MHz, CDCl_3_) δ = 177.33 (C-Au), 153.28 (C^6^=O), 150.71 (C^2^=O), 138.10 (C^4^), 113.18 (C^5^), 46.47 (N^9^CH_2_), 37.98 (N^7^Me), 31.65 (N^3^Me), 28.76 (N^1^Me), 17.36 (Me). High-resolution positive-ion ESMS (*m*/*z*): calculated for [*M+Na*]^+^ 477.0369; found 477.0366. X-ray quality crystals were obtained by slow diffusion of Et_2_O into a CH_2_Cl_2_ solution of the compound.

**[RuCl_2_(NHC)(*p*-cym)] (15)**

This was prepared as described above using complex **9** (0.0438 g, 0.099 mmol) and [RuCl_2_(*p*-cym)]_2_ (0.0294 g, 0.048 mmol). A brown product was obtained in 72% yield (0.0431 g). ^1^H NMR (400 MHz, CDCl_3_, 223 K) δ = 7.30–7.48 (m, 3 H, Ph), 6.89 (m, 2 H, Ph), 6.31 (d, 1 H, *J* = 17.0 Hz, N^7^CH_2_), 5.96 (d, 1H, *J* = 17.0 Hz, N^7^CH_2_), 5.44–5.53 (m, 1 H, *p*-cym), 5.33–5.44 (m, 1 H, N^9^CH_2_), 5.19–5.29 (m, 2 H, *p*-cym), 5.17–5.18 (m, 1 H, *p*-cym), 4.34–4.49 (m, 1 H, N^9^CH_2_), 3.78 (s, 3 H, N^3^Me), 3.29 (s, 3 H, N^1^Me), 2.65–2.78 (m, 1 H, *p*-cym), 2.04 (s, 3 H, *p*-cym), 1.52 (s, 3 H, Me), 1.30 (d, *J* = 6.3 Hz, 3 H, *p*-cym), 1.19 (d, *J* = 6.3 Hz, 3 H, *p*-cym). ^13^C{^1^H} NMR (100 MHz, CDCl_3_, 223 K) δ = 189.37 (C-Ru), 151.52 (C^6^=O), 150.87 (C^2^=O), 140.88 (C^4^), 138.58 (*ipso*-Ph), 129.19 (Ph), 127.38 (Ph), 123.50 (Ph), 111.12 (C^5^), 107.77 (*p*-cym), 94.64 (*p*-cym), 86.37 (*p*-cym), 86.12 (*p*-cym), 83.42 (*p*-cym), 82.78 (*p*-cym), 53.69 (N^7^CH_2_), 47.18 (N^9^CH_2_), 32.57 (N^3^Me), 30.48 (*p*-cym), 28.67 (N^1^Me), 22.57 (*p*-cym), 22.32 (*p*-cym), 18.01 (*p*-cym), 17.70 (Me). High-resolution positive-ion ESMS (*m*/*z*): calculated for [*M-Cl*]^+^ 569.1257; found 569.1257. X-ray quality crystals were obtained by slow diffusion of hexane into a CH_2_Cl_2_ solution of the compound.

**[RuCl_2_(NHC)(*p*-cym)] (16)**

This was prepared as described above using complex **10** (0.0471 g, 0.107 mmol) and [RuCl_2_(*p*-cym)]_2_ (0.0294 g, 0.048 mmol). A brown product was obtained. As a result, 0.0495 g (76%). ^1^H NMR (400 MHz, CDCl_3_, 223 K) δ = 7.35–7.44 (d, *J* = 7.5 Hz, 2 H, Bn), 6.86 (m, 2 H, Bn), 6.30 (d, 1 H, *J* = 17.2 Hz, N^7^CH_2_), 5.87 (d, *J* = 17.2 Hz, N^7^CH_2_), 5.49–5.52 (m, 1 H, *p*-cym), 5.40–5.42 (m, 1 H, N^9^CH_2_), 5.29–5.31 (m, 1 H, *p*-cym), 5.23–5.25 (m, 2 H, *p*-cym), 4.37–4.50 (m, 1 H, N^9^CH_2_), 3.78 (s, 3 H, N^3^Me), 3.29 (s, 3 H, N^1^Me), 2.65–2.78 (m, 1 H, *p*-cym), 2.05 (s, 3 H, *p*-cym), 1.52 (s, 3 H, Me), 1.30 (d, *J* = 4.3 Hz, 3 H, *p*-cym), 1.22 (d, *J* = 5.7 Hz, 3 H, *p*-cym). ^13^C{^1^H} NMR (100 MHz, CDCl_3_, 223 K) δ = 189.52 (C-Ru), 151.48 (C^6^=O), 150.78 (C^2^=O), 140.97 (C^4^), 137.18 (*ipso*-Bn), 132.82 (Bn), 129.38 (Bn), 125.03 (Bn), 110.90 (C^5^), 108.04 (*p*-cym), 94.68 (*p*-cym), 86.37 (*p*-cym), 86.06 (*p*-cym), 83.26 (*p*-cym), 83.04 (*p*-cym), 53.41 (N^7^CH_2_), 47.23 (N^9^CH_2_), 32.55 (N^3^Me), 30.58 (*p*-cym), 28.69 (N^1^Me), 22.46 (*p*-cym), 18.09 (*p*-cym), 17.76 (Me). High-resolution positive-ion ESMS (*m*/*z*): calculated for [*M-Cl*]^+^ 603.0868; found 603.0874.

**[RuCl_2_(NHC)(*p*-cym)] (17)**

This was prepared as described above using complex **11** (0.0362 g, 0.099 mmol) and [RuCl_2_(*p*-cym)]_2_ (0.0294 g, 0.048 mmol). A brown product was obtained. As a result, 0.0420 g (80%). ^1^H NMR (600 MHz, CDCl_3_) δ = 5.47–5.66 (m, 2 H, *p*-cym), 6.26 (d, *J* = 6.1 Hz, 2 H, *p*-cym), 4.50–4.67 (m, 2 H, N^9^CH_2_), 4.36 (s, 3 H, N^7^Me), 3.77 (s, 3 H, N^3^Me), 3.42 (s, 3 H, N^1^Me), 3.02 (unres. pent, 1 H, *p*-cym), 2.18 (s, 3 H, *p*-cym), 1.41 (t, *J* = 7.3 Hz, 3 H, Me), 1.35 (d, *J* = 6.9 Hz, 3 H, *p*-cym). ^13^C{^1^H} NMR (150 MHz, CDCl_3_) δ = 186.12 (C-Ru), 152.80 (C^6^=O), 151.17 C^2^=O), 140.76 (C^4^), 111.85 (C^5^), 109.36 (*p*-cym), 99.08 (*p*-cym), 86.22 (*p*-cym), 83.03 (*p*-cym), 81.27 (*p*-cym), 80.52 (*p*-cym), 46.70 (N^9^CH_2_), 39.66 (N^7^Me), 32.51 (N^3^Me), 30.89 (*p*-cym), 28.58 (N^1^Me), 23.23 (*p*-cym), 22.11 (*p*-cym), 18.93 (*p*-cym), 17.36 (Me). High-resolution positive-ion ESMS (*m*/*z*): calculated for [*M-Cl*]^+^ 493.0944; found 493.0947.

[RhCl_2_(NHC)(Cp*)] (18)

This was prepared as described above using complex **9** (0.0177 g, 0.040 mmol) and [Rh(Cp*)Cl_2_]_2_ (0.0124 g, 0.020 mmol). A red-brown product was obtained in 53% yield (0.013 g). ^1^H NMR (400 MHz, CDCl_3_) δ = 7.26–7.32 (m, 2 H, *m*-Ph), 7.23 (t, *J* = 7.3 Hz, 1 H, *p*-Ph), 6.89 (d, *J* = 7.3 Hz, 2 H, *o*-Ph), 6.39 (d, *J* = 16.5 Hz, 1 H, N^7^CH_2_), 5.98 (d, *J* = 16.5 Hz, 1 H, N^7^CH_2_), 5.18–5.29 (m, 2 H, N^9^CH_2_), 4.66–4.79 (m, 2 H, N^9^CH_2_), 3.79 (s, 3 H, N^3^Me), 3.24 (s, 3 H, N^1^Me), 1.54 (s, 15 H, Cp*), 1.49 (t, *J* = 7.3 Hz, 3 H, Me). ^13^C{^1^H} NMR (150 MHz, CDCl_3_) δ = 183.71 (d, *J* = 54.5 Hz, C-Rh), 151.73 (C^6^=O), 151.27 (C^2^=O), 141.13 (C^4^), 138.58 (*ipso*-Ph), 128.56 (Ph), 127.12 (Ph), 124.67 (Ph), 111.72 (C^5^), 97.21 (d, *J* = 7.2 Hz, Cp*), 54.78 (N^7^CH_2_), 47.10 (N^9^CH_2_), 32.50 (N^3^Me), 28.60 (N^1^Me), 17.66 (Me), 9.49 (Cp*). High-resolution positive-ion ESMS (*m*/*z*): calculated for [*M+Na*]^+^ 629.0933; found 629.0930. X-ray quality crystals were obtained by slow diffusion of Et_2_O into a CH_2_Cl_2_ solution of the compound.

[RhCl_2_(NHC)(Cp*)] (19)

This was prepared as described above using complex **10** (0.0190 g, 0.040 mmol) and [Rh(Cp*)Cl_2_]_2_ (0.0124 g, 0.020 mmol). As a result, 0.0140 g, (55%) of a red-brown solid was obtained. ^1^H NMR (400 MHz, CDCl_3_) δ = 7.24–7.28 (m, 2 H, B), 6.92 (d, *J* = 7.8 Hz, 2 H, Bn), 6.14–6.29 (s, 1 H, N^7^CH_2_), 5.95–6.10 (s, 1 H, N^7^CH_2_), 5.10–5.25 (s, 1 H, N^9^CH_2_), 4.70–4.90 (s, 1 H, N^9^CH_2_), 3.79 (s, 3 H, N^3^Me), 3.25 (s, 3 H, N^1^Me), 1.56 (s, 15 H, Cp*), 1.49 (t, *J* = 7.2 Hz, 3 H, Me). ^13^C{^1^H} NMR (150 MHz, CDCl_3_) δ = 183.60 (d, *J* = 53.3 Hz, C-Rh), 151.76 (C^6^=O), 151.20 (C^2^=O), 141.34 (C^4^), 136.90 (*ipso*-Bn), 132.93 (Bn), 128.65 (Bn), 126.64 (Bn), 111.52 (C^5^), 97.24 (d, *J* = 7.1 Hz, Cp*), 54.35 (N^7^CH_2_), 47.00 (N^9^CH_2_), 32.51 (N^3^Me), 28.64 (N^1^Me), 17.77 (Me), 9.52 (Cp*). High-resolution positive-ion ESMS (*m*/*z*): calculated for [*M-Cl*]^+^ 605.0957; found 605.0957. X-ray quality crystals were obtained by slow diffusion of Et_2_O into a CH_2_Cl_2_ solution of the compound.

[RhCl_2_(NHC)(Cp*)] (20)

This was prepared as described above using complex **11** (0.0322 g, 0.0881 mmol) and [Rh(Cp*)Cl_2_]_2_ (0.0248 g, 0.0400 mmol). As a result, 0.0355 g, (76%) of a red-brown solid was obtained. ^1^H NMR (400 MHz, CDCl_3_) δ = 4.73–5.09 (m, 2 H, N^9^CH_2_), 4.33 (s, 3 H, N^7^Me), 3.75 (s, 3 H, N^3^Me), 3.39 (s, 3 H, N^1^Me), 1.66 (s, 15 H, Cp*), 1.39 (t, *J* = 7.2 Hz, 3 H, Me). ^13^C{^1^H} NMR (150 MHz, CDCl_3_) δ = 152.94 (C^6^=O), 151.20 (C^2^=O), 140.92 (C^4^), 112.06 (C^5^), 96.98 (d, *J* = 7.0 Hz, Cp*), 46.41 (N^9^CH_2_), 39.14 (N^7^Me), 32.54 (N^3^Me), 28.61 (N^1^Me), 17.55 (Me), 9.59 (Cp*). The carbene-carbon signal could not be detected. High-resolution positive-ion ESMS (*m*/*z*): calculated for [*M-Cl*]^+^ 495.1034; found 495.1030.

**[RhCl(NHC)(cod)] (21)**

This was prepared as described above using complex **9** (0.0352 g, 0.0797 mmol) and [Rh(cod)Cl]_2_ (0.0196 g, 0.0397 mmol). A yellow product was obtained in 48% yield (0.021 g). ^1^H NMR (400 MHz, CDCl_3_) δ = 7.44 (d, *J* = 7.3 Hz, 2 H, *o*-Ph), 7.33 (t, *J* = 7.3 Hz, 2 H, *m*-Ph), 7.23–7.29 (m, 1 H, *p*-Ph), 6.25 (d, *J* = 14.8 Hz, 1 H, N^7^CH_2_), 6.10 (d, *J* = 14.8 Hz, 1 H, N^7^CH_2_), 5.42–5.53 (m, 1 H, N^9^CH_2_), 5.16–5.23 (m, 1 H, cod), 5.07–5.15 (m, 1 H, cod), 4.99–5.07 (m, 1 H, N^9^CH_2_), 3.78 (s, 3 H, N^3^Me), 3.30 (s, 3 H, N^1^Me), 3.26–3.32 (m, 3 H, cod), 2.99–3.06 (m, 1 H, cod), 2.25–2.52 (m, 3 H, cod), 1.83–2.10 (m, 4 H, cod), 1.71–1.76 (m, 1 H, cod), 1.69 (t, *J* = 7.2 Hz, 3 H, Me). ^13^C{^1^H} NMR (100 MHz, CDCl_3_) δ = 193.49 (C-Rh), 152.14 (C^6^=O), 150.81 (C^2^=O), 140.08 (C^4^), 136.92 (*ipso*-Ph), 128.50 (*m*-Ph), 127.66 (*p*-Ph), 127.32 (*o*-Ph), 109.00 (C^5^), 100.89 (d, *J* = 6.8 Hz, cod), 100.12 (d, *J* = 6.8 Hz, cod), 69.84 (d, *J* = 14.3 Hz, cod), 69.67 (d, *J* = 14.3 Hz, cod), 53.71 (N^7^CH_2_), 46.33 (N^9^CH_2_), 33.53 (cod), 31.89 (cod), 31.59 (N^3^Me), 28.55 (cod), 28.11 (N^1^Me), 29.25 (cod), 17.67 (Me). High-resolution positive-ion ESMS (*m*/*z*): calculated for [*M-Cl*]^+^ 509.1424; found 509.1429.

**[RhCl(NHC)(cod)] (22)**

This was prepared as described above using complex **10** (0.0635 g, 0.1334 mmol) and [Rh(cod)Cl]_2_ (0.0329 g, 0.0604 mmol). As a result, 0.0520 g (72%) of a yellow solid was obtained. ^1^H NMR (400 MHz, CDCl_3_) δ = 7.47 (d, *J* = 8.4 Hz, 2 H, Bn), 7.29 (m, 2 H, Bn), 6.26 (d, *J* = 14.7 Hz, 1 H, N^7^CH_2_), 5.99 (d, *J* = 14.7 Hz, 1 H, N^7^CH_2_), 5.40–5.50 (m, 1 H, N^9^CH_2_), 5.10–5.23 (m, 2 H, cod), 4.99–5.10 (m, 1 H, N^9^CH_2_), 3.78 (s, 3 H, N^3^Me), 3.30 (s, 3 H, N^1^Me), 3.00–3.10 (m, 1 H, cod), 2.28–2.52 (m, 3 H, cod), 2.10–2.20 (m, 1 H, cod), 1.75–2.08 (m, 4 H, cod), 1.69 (t, *J* = 7.2 Hz, 3 H, Me). ^13^C{^1^H} NMR (100 MHz, CDCl_3_) δ = 193.28 (C-Rh), 152.12 (C^6^=O), 150.74 (C^2^=O), 140.23 (C^4^), 135.15 (*ipso*-Bn), 133.66 (Bn), 129.13 (Bn), 128.65 (Bn), 109.94 (C^5^), 101.08 (d, *J* = 6.7 Hz, cod), 100.54 (d, *J* = 6.7 Hz, cod), 69.98 (d, *J* = 13.8 Hz, cod), 69.66 (d, *J* = 13.8 Hz, cod), 53.19 (N^7^CH_2_), 46.35 (N^9^CH_2_), 33.41 (cod), 33.06 (cod), 31.58 (N^3^Me), 29.22 (cod), 28.55 (N^1^Me), 28.19 (cod), 16.89 (Me). High-resolution positive-ion ESMS (*m*/*z*): calculated for [*M-Cl*]^+^ 543.1034; found 543.1037. X-ray quality crystals were obtained by slow diffusion of hexane into a CH_2_Cl_2_ solution of the compound.

**[RhCl(NHC)(cod)] (23)**

This was prepared as described above using complex **11** (0.0291 g, 0.0796 mmol) and [Rh(cod)Cl]_2_ (0.0196 g, 0.0360 mmol). As a result, 0.0370 g (99%) of a yellow solid was obtained. ^1^H NMR (400 MHz, CDCl_3_) δ = 5.40–5.31 (m, 1 H, N^9^CH_2_), 5.09 (dd, *J* = 21.0, 5.3 Hz, 2 H, cod), 4.90 (m, 1 H, N^9^CH_2_), 4.39 (s, 3 H, N^7^Me), 3.76 (s, 3 H, N^3^Me), 3.48–3.39 (m, 2 H, cod), 3.35 (s, 3 H, N^1^Me), 2.53–2.37 (m, 4 H, cod), 2.01 (d, *J* = 9.5 Hz, 4 H, cod), 1.61 (t, *J* = 7.2 Hz, 3 H, Me). ^13^C{^1^H} NMR (150 MHz, CDCl_3_) δ = 191.11 (br. m, C-Rh), 152.81 (C^6^=O), 150.79 (C^2^=O), 139.61 (C^4^), 110.51 (C^5^), 100.59 (d, *J* = 6.2 Hz, cod), 100.30 (d, *J* = 6.2 Hz, cod), 69.62 (cod), 68.88 (cod), 46.04 (N^9^CH_2_), 37.42 (N^7^CH_2_), 32.79 (d, *J* = 5.6 Hz, cod), 31.48 (N^3^Me), 28.81 (N^1^Me), 28.72 (cod), 28.45 (cod), 28.00 (cod), 16.93 (Me). High-resolution positive-ion ESMS (*m*/*z*): calculated for [*M-Cl*]^+^ 433.1111; found 433.1114.

**X-ray crystallography**

Diffraction data for complex **15** was collected at 100 K using a Bruker-AXS Kappa Mach3 APEX-II system located in front of a Iμs microfocus source equipped with Incoatec Helios mirrors for Mo radiation. For data integration the APEX3 package was employed. For data scaling and absorption correction SADABS was used [61]. Diffraction data for complexes **5**, **7**, **9**, **12**–**14** and **18**–**22** was collected at 150 K using a Rigaku Oxford Diffraction Gemini E Ultra diffractometer, equipped with an EOS CCD area detector and a four-circle kappa goniometer. Data integration, scaling and empirical absorption correction was carried out using the CrysAlis Pro program package [62]. Data for complex **10** was collected at beamline P11, PETRA III DESY Hamburg. For data integration XDS [63] (version 31 January 2020) and for scaling as well as absorption correction SADABS were used. All structures were solved by direct methods or intrinsic phasing (SHELXT [64]) and refined using SHELXL [65] as implemented in the Olex2 graphical user interface [66]. Crystallographic and refinement details are collected in Table S1 in Supplementary Materials.

## 4. Conclusions

We present, herein, a simple method to ethylate various xanthine derivatives at position N9 using ethyl tosylate or diethyl sulfate. High yields of the pure xanthinium salts were obtained in short times with minimal work-up. The anions could be exchanged to give the corresponding PF_6_^−^ and Cl^−^ salts. The xanthinium PF_6_^−^ salts reacted with Ag_2_O in the presence of NH_3_ or Et_2_NH to furnish either silver carbene complexes with ammonia co-ligands or bis(carbene) silver compounds, respectively. The xanthinium chloride salts gave the corresponding neutral Ag-carbene complexes. These silver compounds were used successfully to transfer the carbene ligands to a variety of other metals including Au(I), Ru(II), Rh(I) and Rh(III). Work is ongoing to incorporate other groups into the xanthine-backbone by this approach and to examine possible applications of these metal-carbenes.

## Data Availability

Data sharing is not applicable.

## Supplementary Materials

The following are available online, Table S1: Crystallographic and refinement details for all X-ray structures reported herein. Figures S1–S70: NMR spectra of the compounds.

## Sample Availability

Samples of the compounds are available from the authors.
