# Supplementary material for: Facile N9-Alkylation of Xanthine Derivatives and Their Use as Precursors for N-Heterocyclic Carbene Complexes
Source: Molecules. 2021 Jun 17;26(12):3705. doi: 10.3390/molecules26123705 (PMC8234118; doi:10.3390/molecules26123705)
Supplement: Supplementary file 1 [file molecules-26-03705-s001.zip › molecules-1258248-supplementary.pdf]

Supplementary Material

Facile N9-alkylation of xanthine derivatives and their use as precursors for N-heterocyclic carbene complexes.

Moloud Mokfi [1], Jörg Rust [2], Christian W. Lehmann [2] and Fabian Mohr [1,*]

[1] Fakultät für Mathematik und Naturwissenschaften, Anorganische Chemie, Bergische Universität Wuppertal, Gaußstr. 20, 42119 Wuppertal, Germany.

[2] Chemische Kristallographie und Elektronenmikroskopie, Max-Planck-Institut für Kohlenforschung, Kaiser-Wilhelm-Platz 1, 45470 Mülheim an der Ruhr, Germany.

Contents

Table S1. Crystallographic and refinement details.

Figures S1-S70. NMR spectra of the compounds.

Table S1. Crystallographic and refinement details.

	[Ag(NHC)(NH_3)]PF_6 (**5**)	[Ag(NHC)$_2$]PF_6 (**7**)	[AgCl(NHC)]·(**9**)	[AgCl(NHC)]·(**10**)	[AuCl(NHC)]·(**12**)	[AuCl(NHC)]·(**13**)
CCDC code	2073071	2073072	2073073	2075635	2073074	2073075
Empirical formula	$C_{16}H_{20}ClF_6N_5O_2PAg$	$C_{32}H_{36}F_6N_8O_4PAg$	$C_{32}H_{36}Cl_2N_8O_4Ag_2$	$C_{48}H_{51}Cl_6N_{12}O_6Ag_3$	$C_{16}H_{18}ClN_4O_2Au$	$C_{16}H_{17}Cl_2N_4O_2Au$
Formula weight	602.66	849.53	883.33	1428.32	530.76	565.20
Crystal system	Orthorhombic	Orthorhombic	Triclinic	Triclinic	Monoclinic	Monoclinic
Space group	$Pna2_1$	$Pca2_1$	P-1	P-1	$P2_1/n$	$P2_1/n$
a/Å	18.2489(4)	20.7353(5)	8.7313(5)	12.7960(4)	11.3106(2)	11.5439(4)
b/Å	13.0368(3)	10.7229(2)	10.1183(6)	14.2460(6)	9.1372(2)	9.3535(4)
c/Å	9.0580(3)	15.4374(4)	11.1408(6)	17.7630(8)	16.0606(4)	16.3214(6)
α/°	90	90	107.453(5)	112.002(2)	90	90
β/°	90	90	103.485(5)	97.983(5)	91.485(2)	92.204(4)
γ/°	90	90	104.032(5)	100.642(6)	90	90
Volume/Å^3	2154.97(9)	3432.37(15)	859.72(10)	2872.6(2)	1659.26(6)	1761.01(12)
Z	4	4	1	2	4	4
ρ_{calc} mg/mm^3	1.858	1.644	1.706	1.651	2.125	2.132
μ/mm^{-1}	1.210	0.717	1.344	0.932	9.044	8.675
F(000)	1200.0	1728.0	444.0	1428	1016.0	1080.0
Crystal size/mm^3	0.02 × 0.05 × 0.10	0.04 × 0.08 × 0.13	0.03 × 0.06 × 0.12	0.04 × 0.08 × 0.09	0.03 × 0.09 × 0.10	0.04 × 0.04 × 0.06
2Θ range	5.476 to 59.024°	5.026 to 59.06°	4.778 to 59.056°	1.108 to 26.902°	5.13 to 58.766°	4.996 to 58.462°
Reflections collected	7489	11608	7649	60327	8241	9461
Independent reflections	3933	6284	4014	17428	3825	4061
Data/restraints/parameters	3933/235/348	6284/1/494	4014/0/220	17428/0/686	3825/0/220	4061/0/229
Goodness-of-fit on F^2	1.028	1.052	1.066	1.031	1.064	1.029
Final R indices [I>2σ(I)]	R_1 = 0.0348<br>wR_2 = 0.0786	R_1 = 0.0347<br>wR_2 = 0.0705	R_1 = 0.0294<br>wR_2 = 0.0634	R_1 = 0.0558<br>wR_2 = 0.1522	R_1 = 0.0246<br>wR_2 = 0.0493	R_1 = 0.0243<br>wR_2 = 0.0403
Largest difference peak/hole/eÅ^3	0.67/-0.53	0.65/-0.82	0.48/-0.33	2.2/-2.5	1.29/-0.91	0.69/-1.10

Table S1 continued. Crystallographic and refinement details.

	[AuCl(NHC)]·(**14**)	[RuCl$_2$(NHC)(p-cym)] (**15**)	[Cp*RhCl$_2$(NHC)] (**18**)	[Cp*RhCl$_2$(NHC)] (**19**)	[RhCl(NHC)(cod)]·(**22**)
CCDC code	2073076	2075634	2073077	2073078	2073079
Empirical formula	$C_{10}H_{14}ClN_4O_2Au$	$C_{53}H_{65}Cl_7N_8O_4Ru_2$	$C_{26}H_{33}Cl_2N_4O_2Rh$	$C_{27}H_{34}Cl_5N_4O_2Rh$	$C_{24}H_{29}Cl_2N_4O_2Rh$
Formula weight	454.67	1328.42	607.37	726.74	579.32
Crystal system	Monoclinic	Monoclinic	Monoclinic	Monoclinic	Monoclinic
Space group	I2/a	$P2_1/c$	$P2_1/c$	$P2_1/c$	$P2_1/c$
a/Å	13.8722(4)	13.3442(5)	9.5291(4)	9.4877(3)	9.4637(7)
b/Å	13.3255(3)	20.1961(7)	21.4169(12)	21.5981(8)	17.8884(17)
c/Å	14.5932(4)	20.7032(7)	14.7086(7)	14.6634(5)	15.0763(16)
α/°	90	90	90	90	90
β/°	107.474(3)	95.414(2)	102.977(4)	101.454(3)	99.401(9)
γ/°	90	90	90	90	90
Volume/Å^3	2573.12(12)	5554.6(3)	2025.1(3)	2944.93(18)	2518.0(4)
Z	8	4	4	4	4
ρ_{calc} mg/mm^3	2.347	1.589	1.379	1.639	1.528
μ/mm^{-1}	11.642	0.933	0.794	1.067	0.919
F(000)	1712.0	2712	1248.0	1480.0	1184.0
Crystal size/mm^3	0.03 × 0.04 × 0.16	0.012 × 0.031 × 0.081	0.02 × 0.04 × 0.05	0.02 × 0.05 × 0.07	0.02 × 0.04 × 0.12
2Θ range	6.114 to 58.754°	1.412 to 30.780°	4.748 to 58.656°	4.718 to 58.962°	4.922 to 58.876°
Reflections collected	6494	175605	15933	17628	14472
Independent reflections	2964	17268	6884	6803	5791
Data/restraints/parameters	2964/0/167	17268/0/691	6884/0/324	6803/0/360	5791/0/301
Goodness-of-fit on F^2	1.086	1.017	1.020	1.020	1.141
Final R indices [I>2σ(I)]	R_1 = 0.0216<br>wR_2 = 0.0494	R_1 = 0.0515<br>wR_2 = 0.1287	R_1 = 0.0385<br>wR_2 = 0.0779	R_1 = 0.0305<br>wR_2 = 0.0617	R_1 = 0.0653<br>wR_2 = 0.1215
Largest difference peak/hole/eÅ^3	0.89/-1.16	2.6/-1.8	0.58/-0.51	0.45/-0.43	1.25/-0.78

NMR spectra

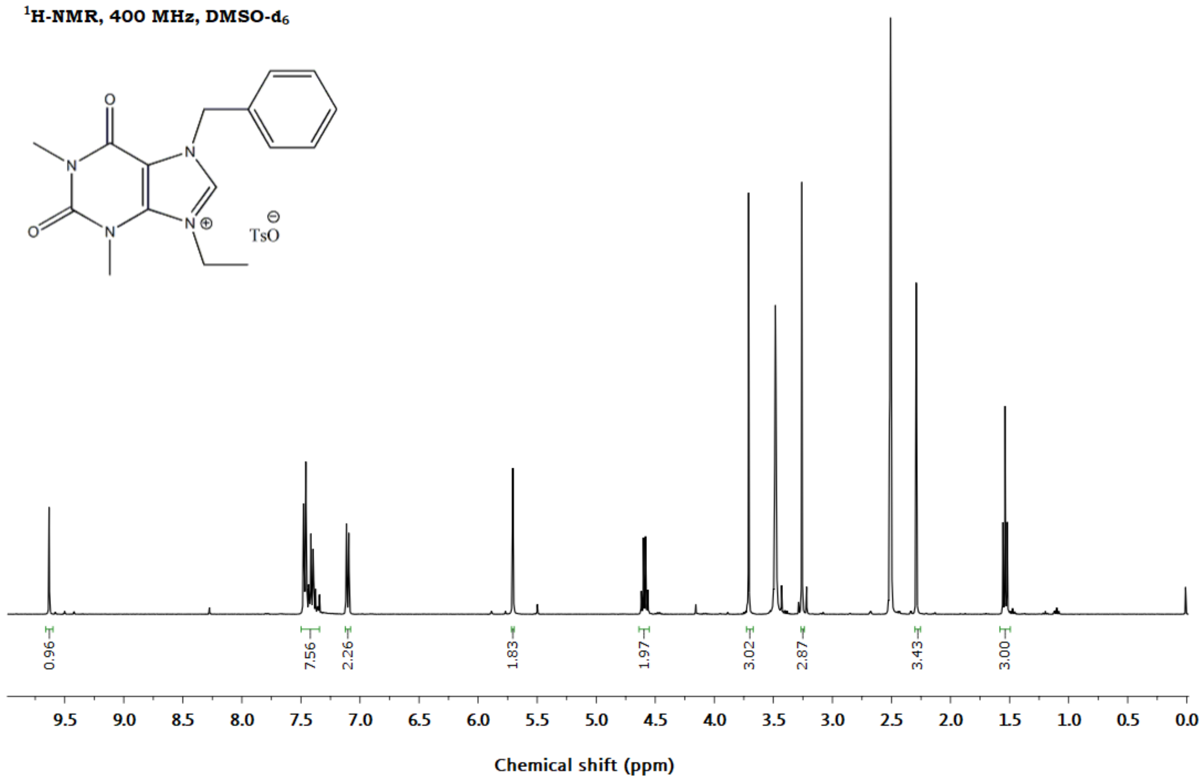

Figure S1

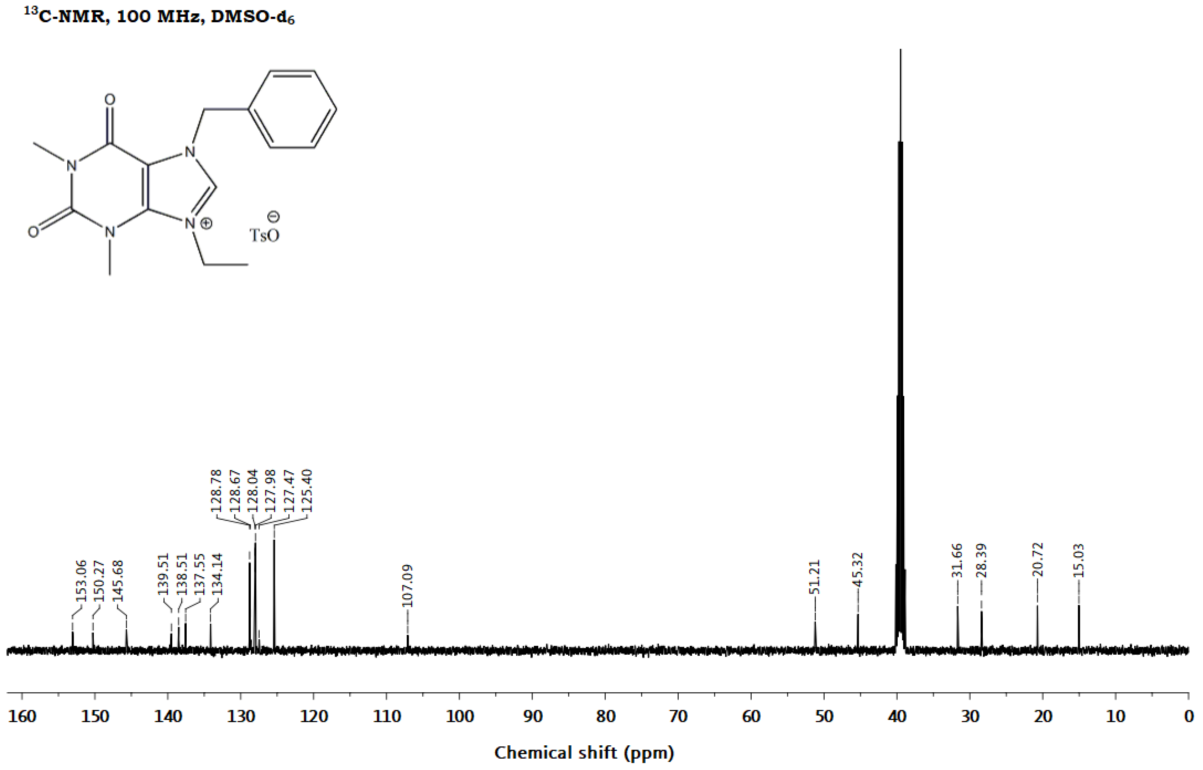

Figure S2

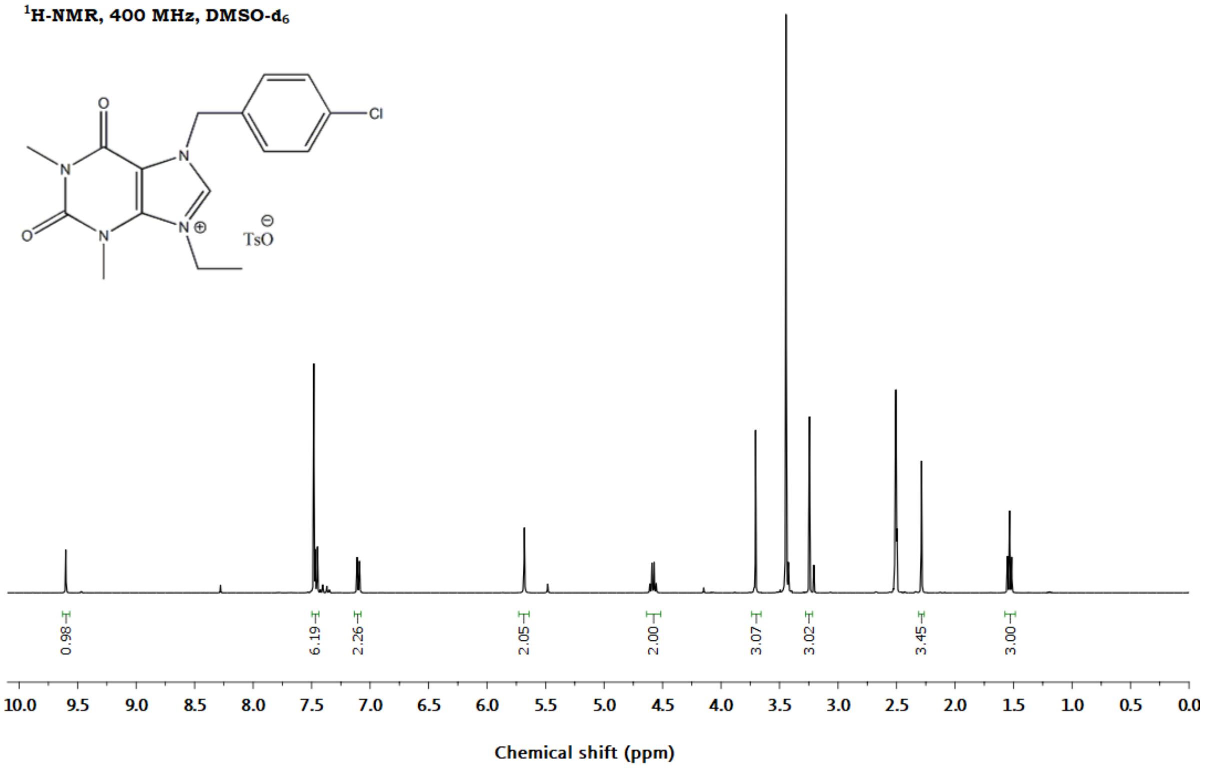

Figure S3

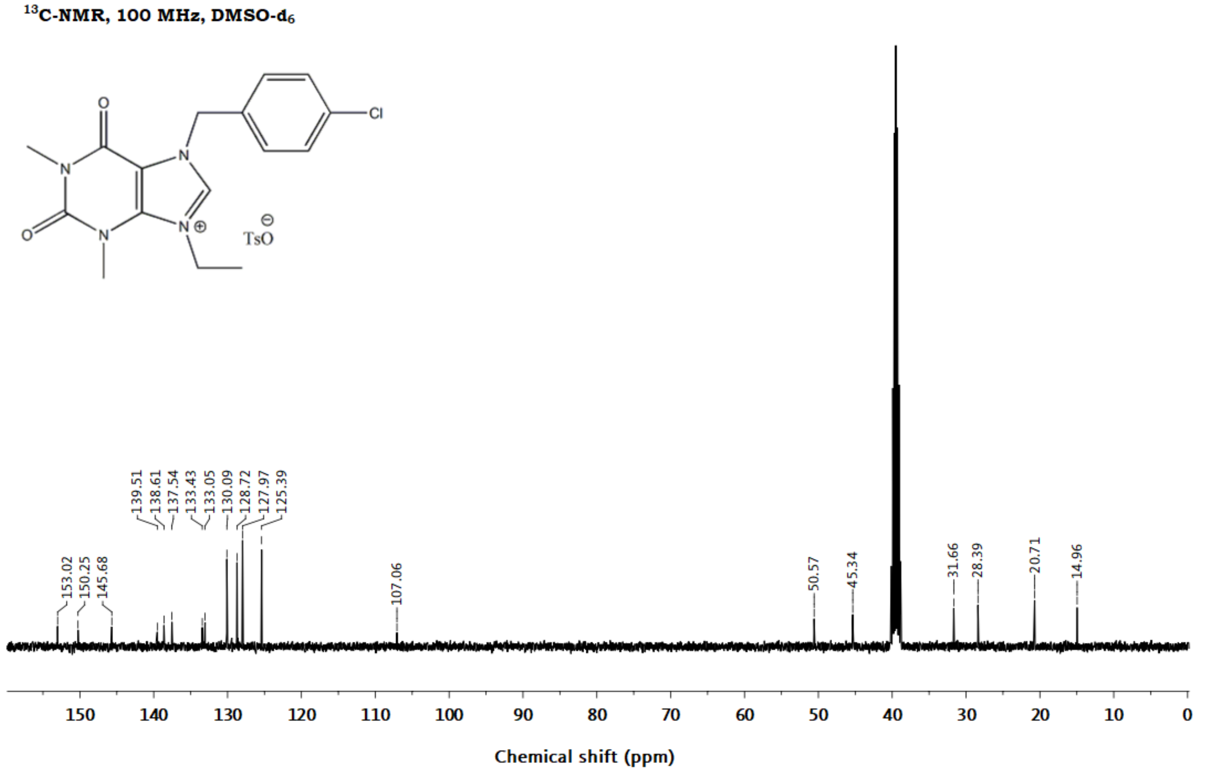

Figure S4

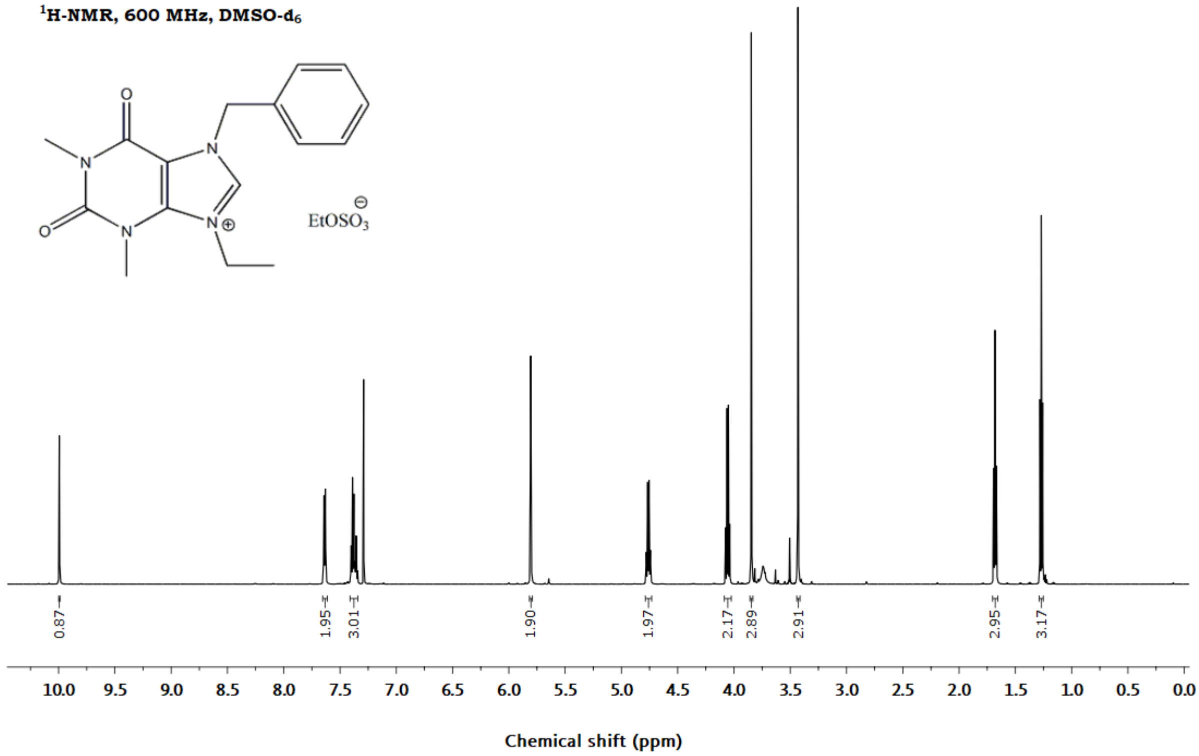

Figure S5

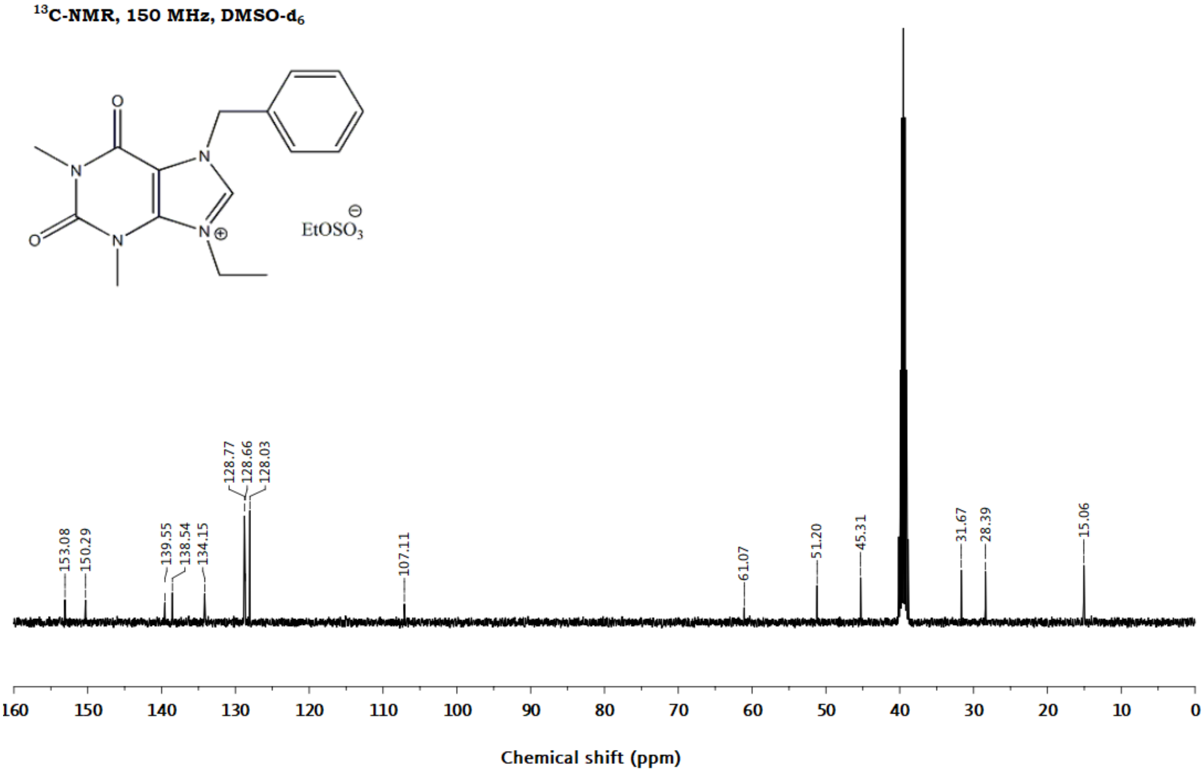

Figure S6

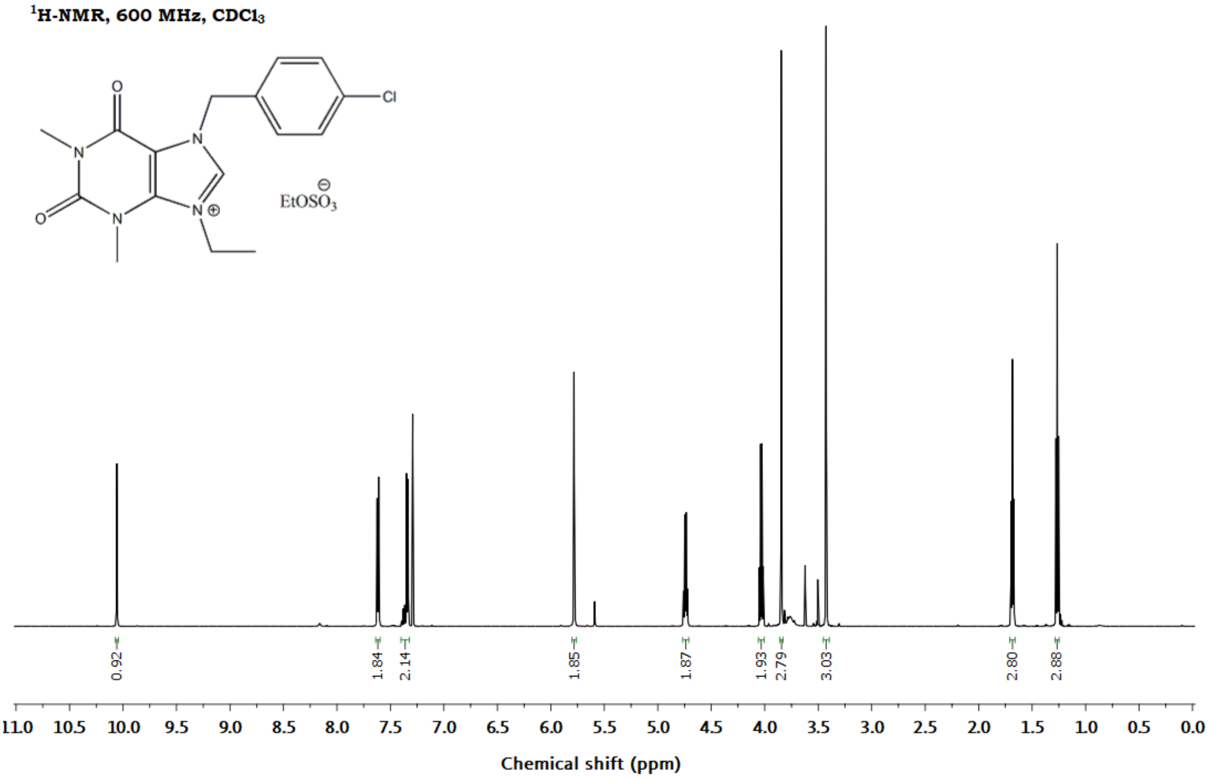

Figure S7

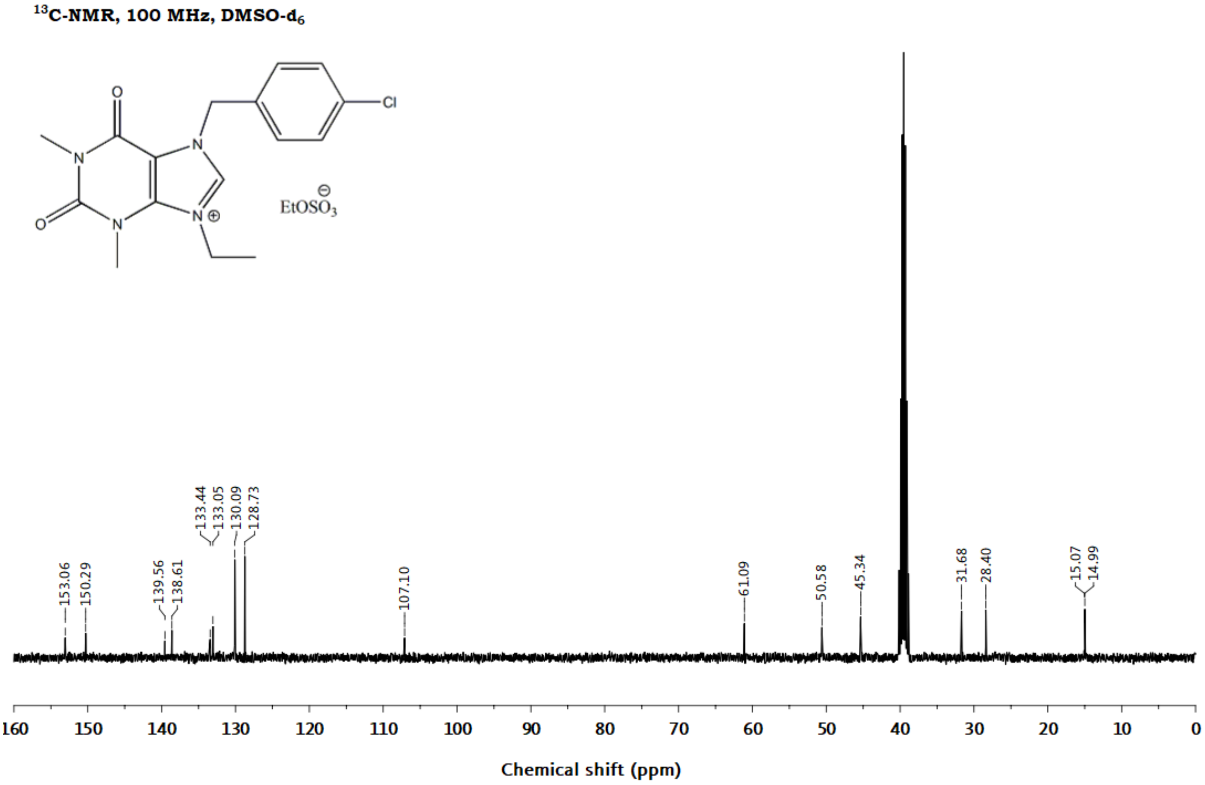

Figure S8

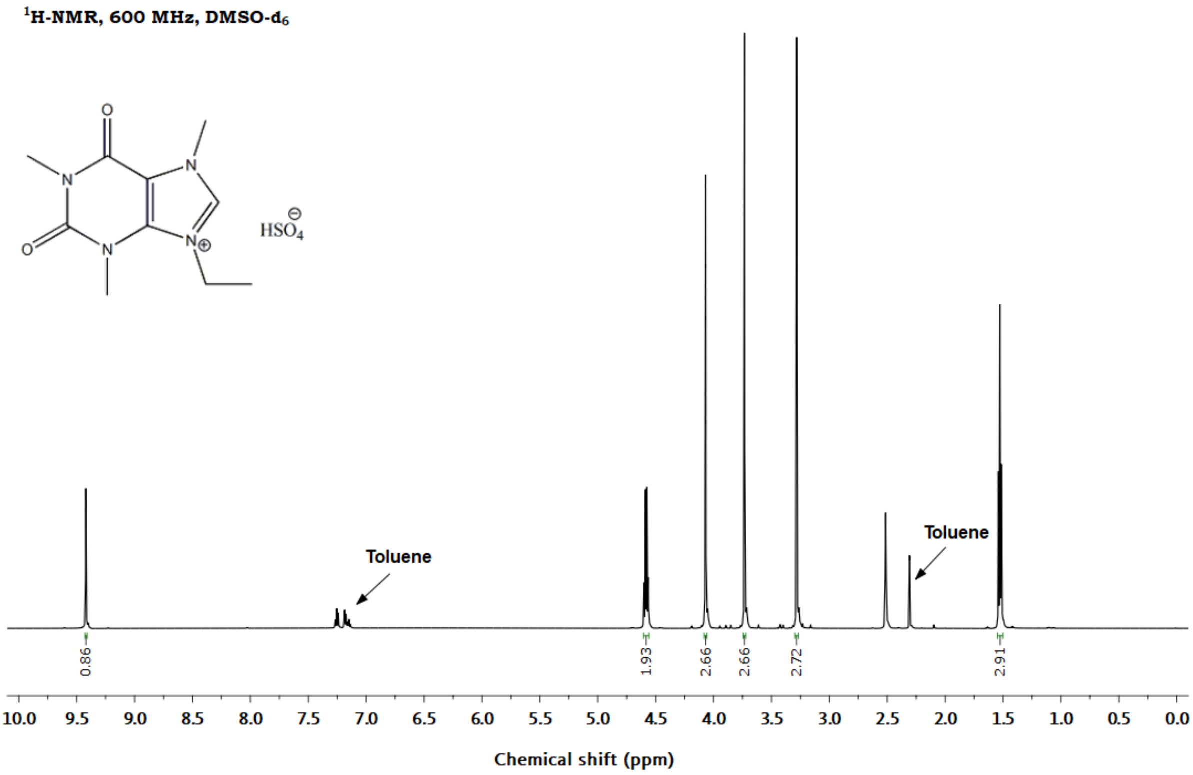

Figure S9

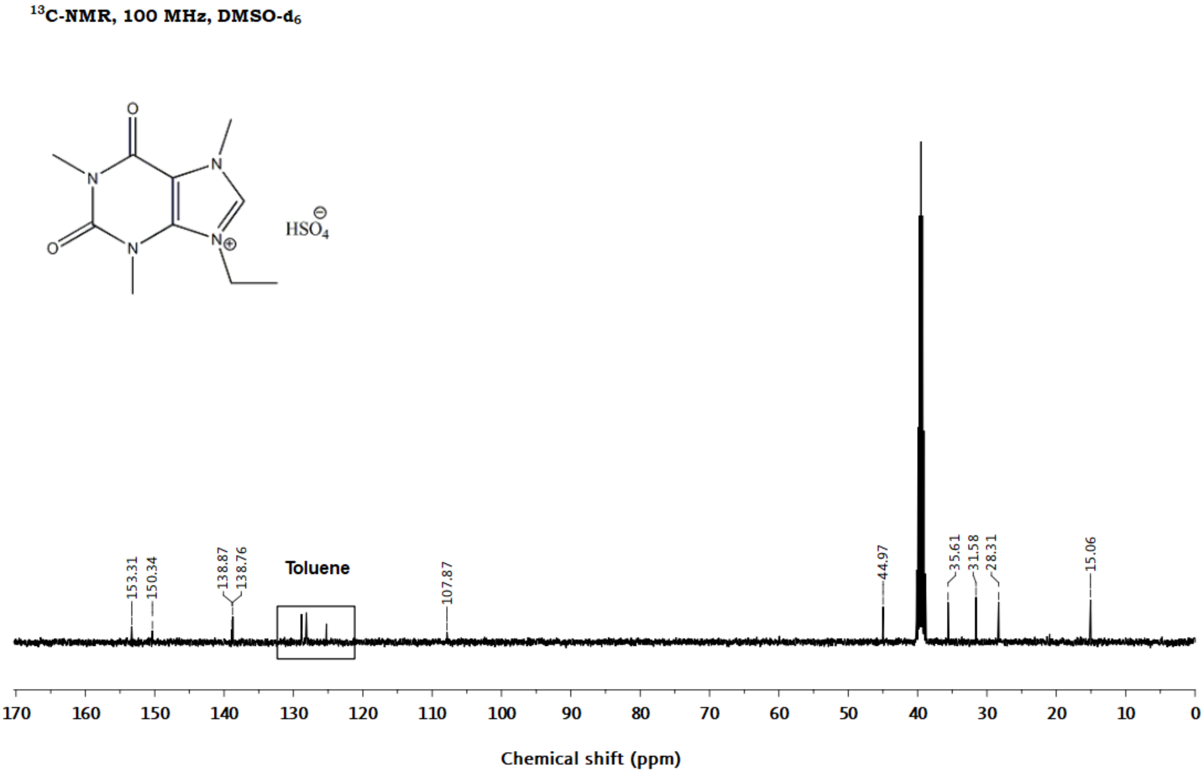

Figure S10

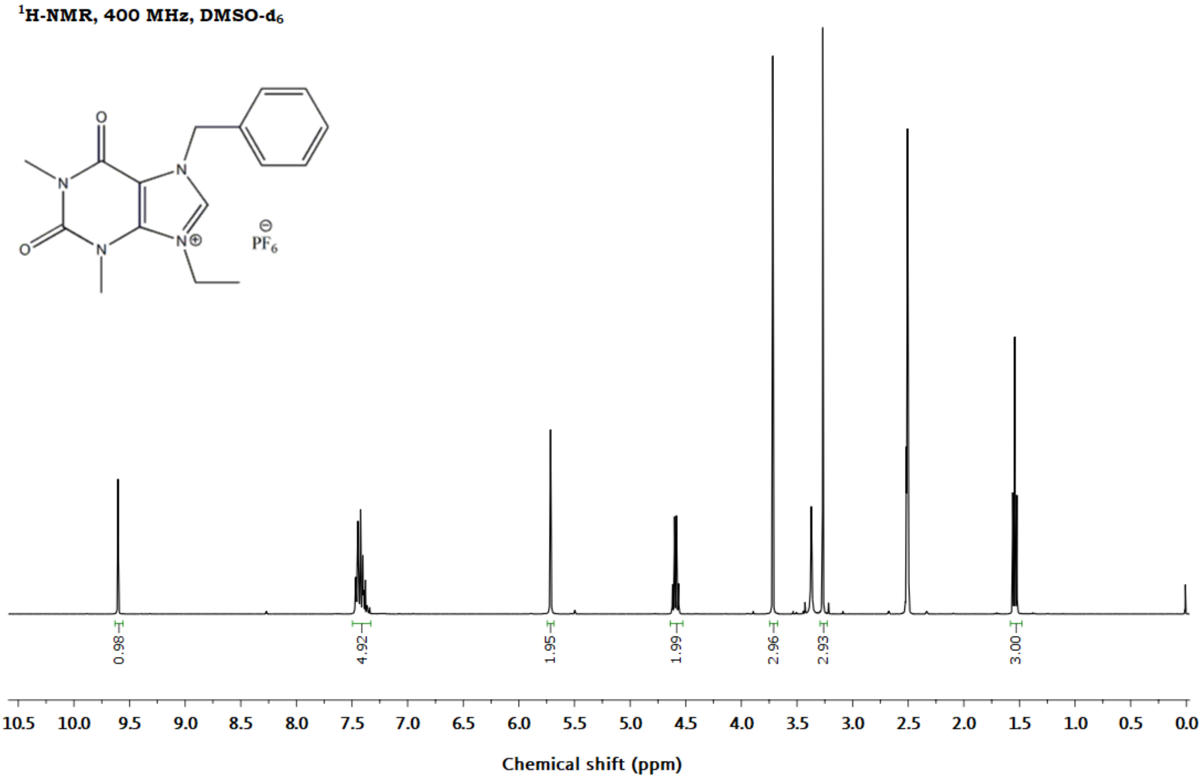

Figure S11

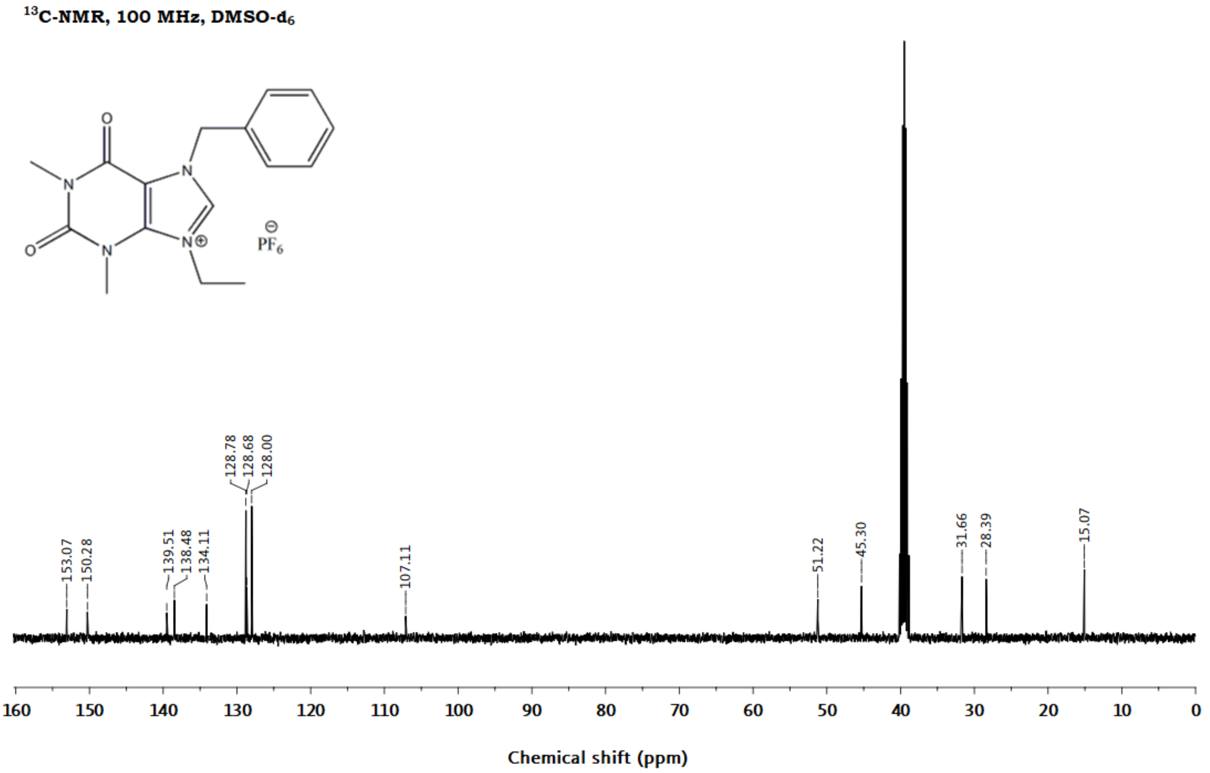

Figure S12

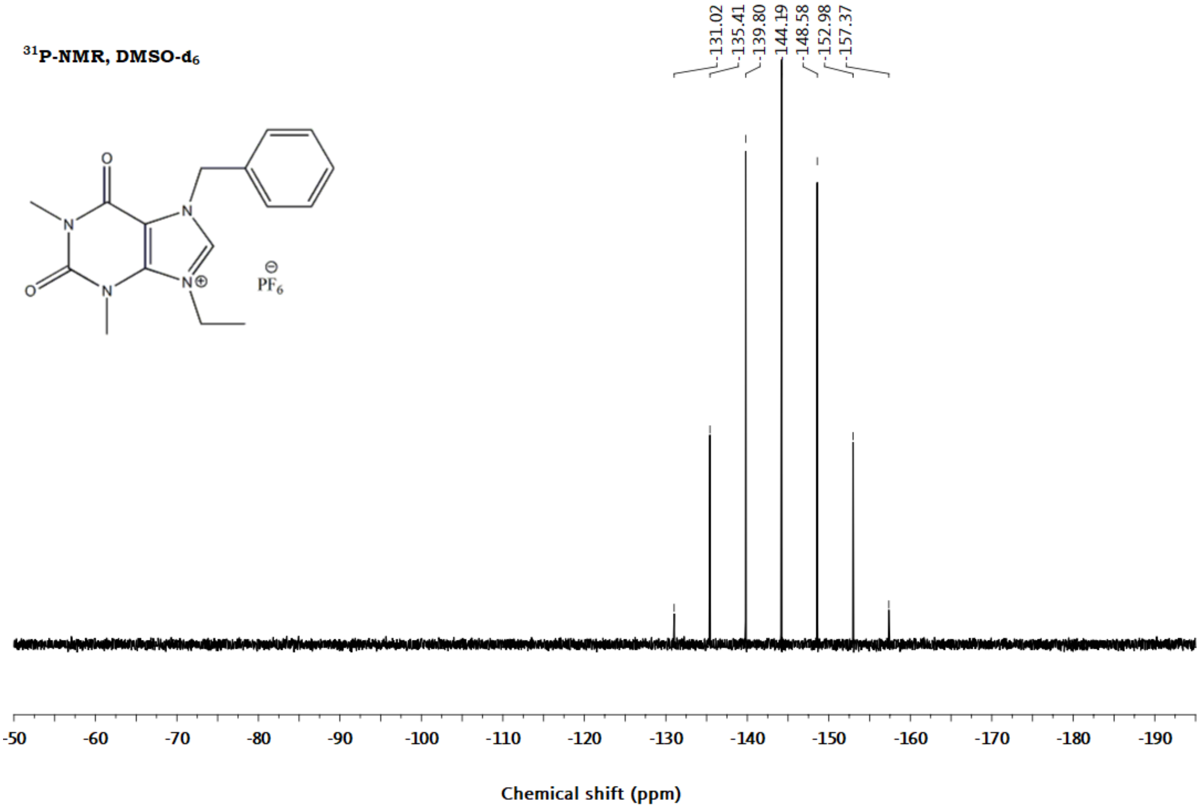

^{31}P-NMR, DMSO-d$_6$
-131.02
-135.41
-139.80
-144.19
-148.58
-152.98
-157.37
PF$_6$
-50
-60
-70
-80
-90
-100
-110
-120
-130
-140
-150
-160
-170
-180
-190
Chemical shift (ppm)

Figure S13

^{1}H-NMR, 400 MHz, DMSO-d_6

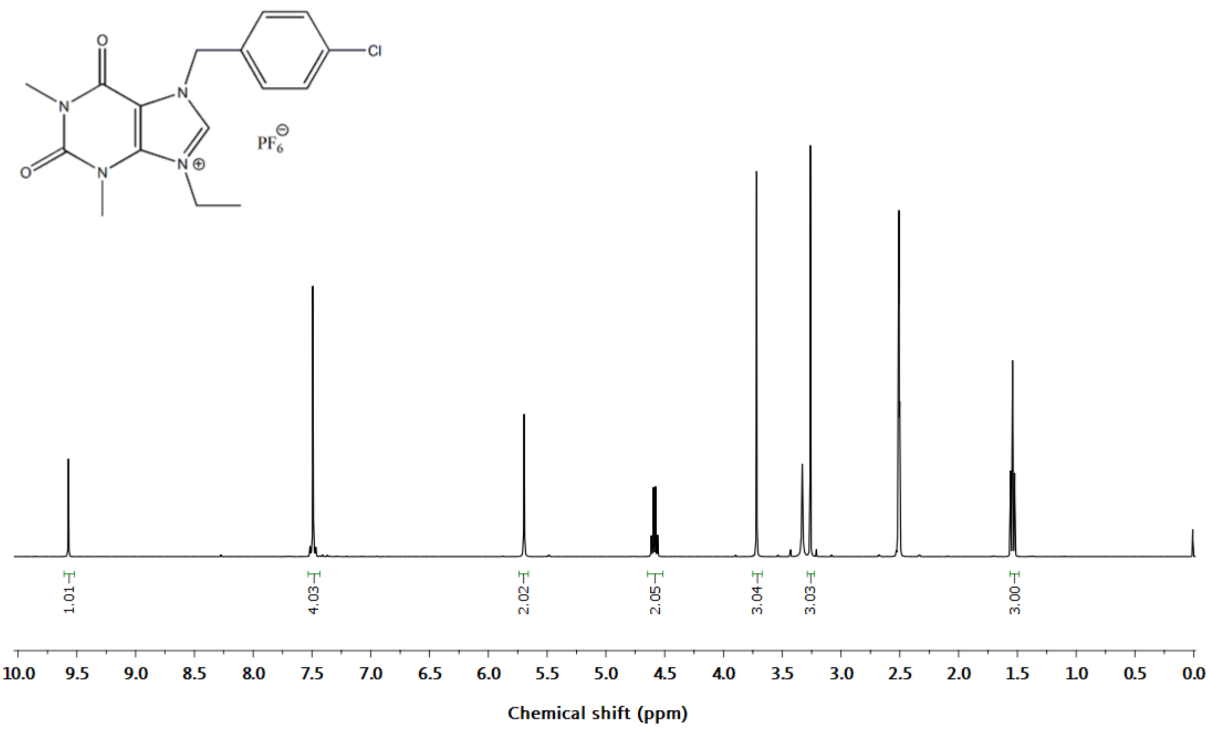

Figure S14

^{13}C-NMR, 100 MHz, DMSO-d_6

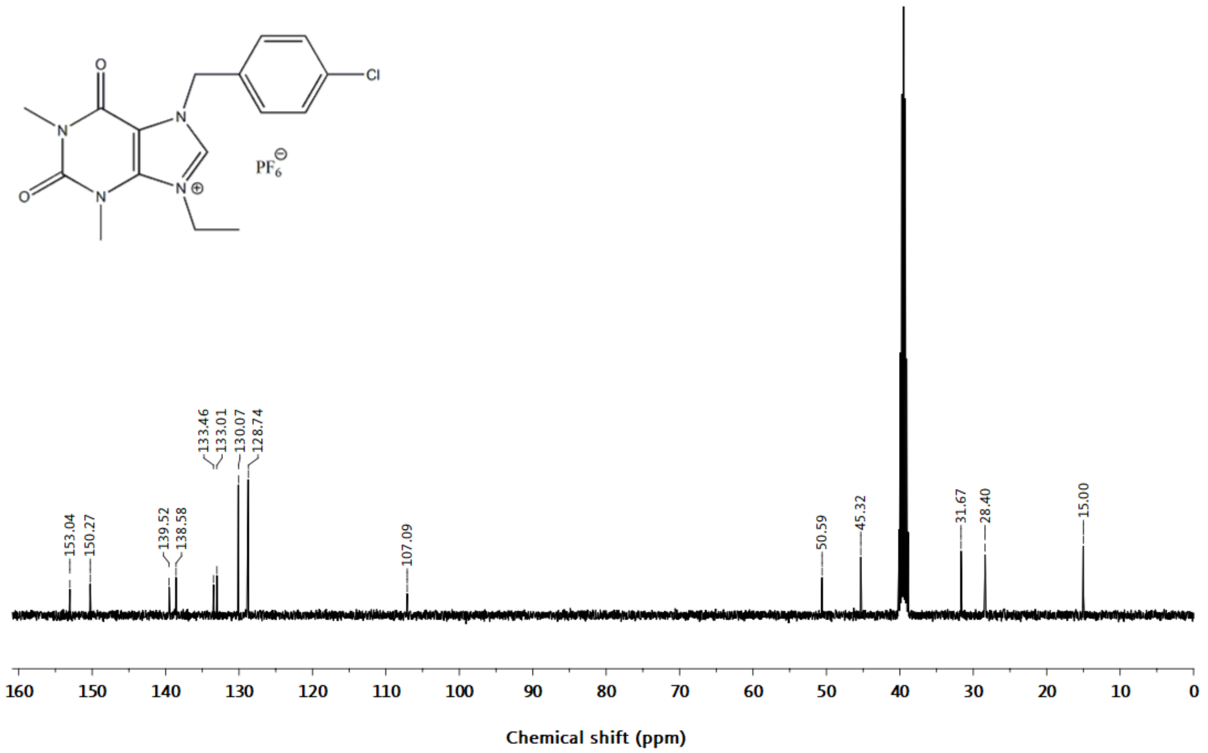

Figure S15

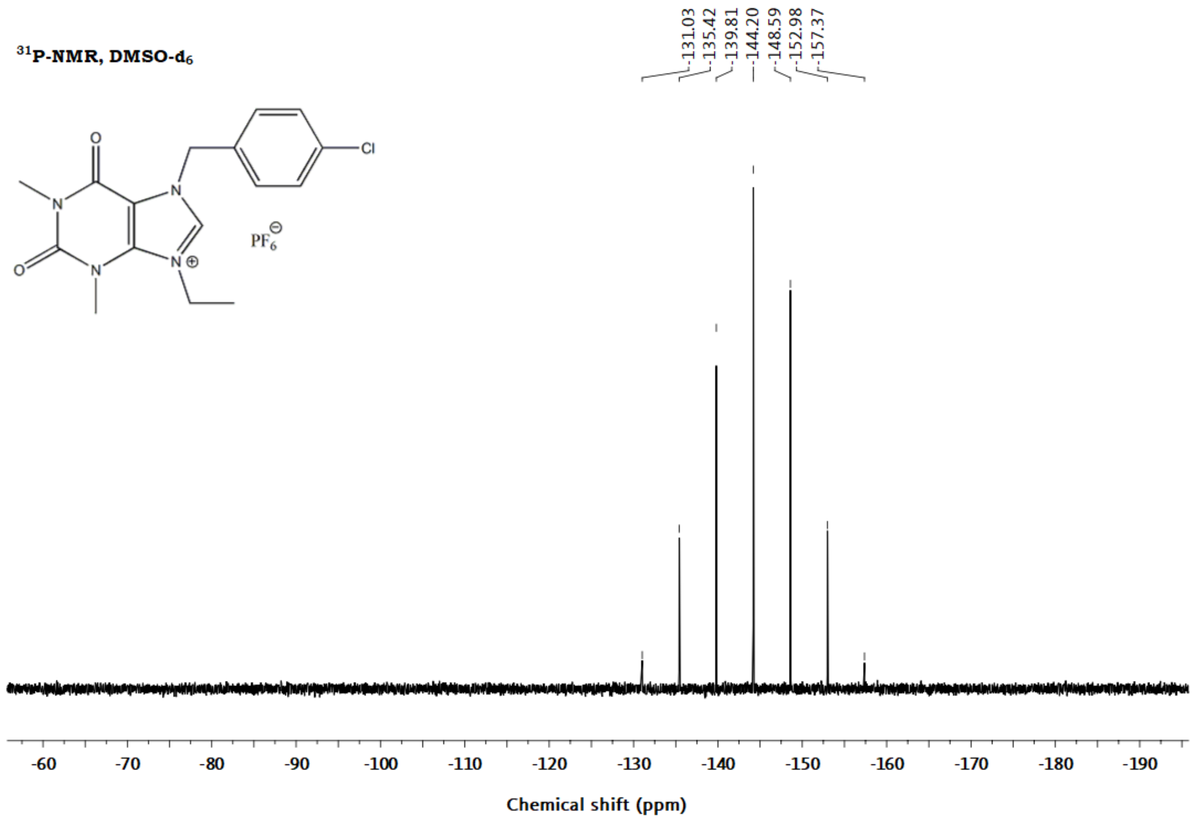

^{31}P-NMR, DMSO-d$_6$
PF$_6^{\ominus}$
-131.03
-135.42
-139.81
-144.20
-148.59
-152.98
-157.37
-60 -70 -80 -90 -100 -110 -120 -130 -140 -150 -160 -170 -180 -190
Chemical shift (ppm)

Figure S16

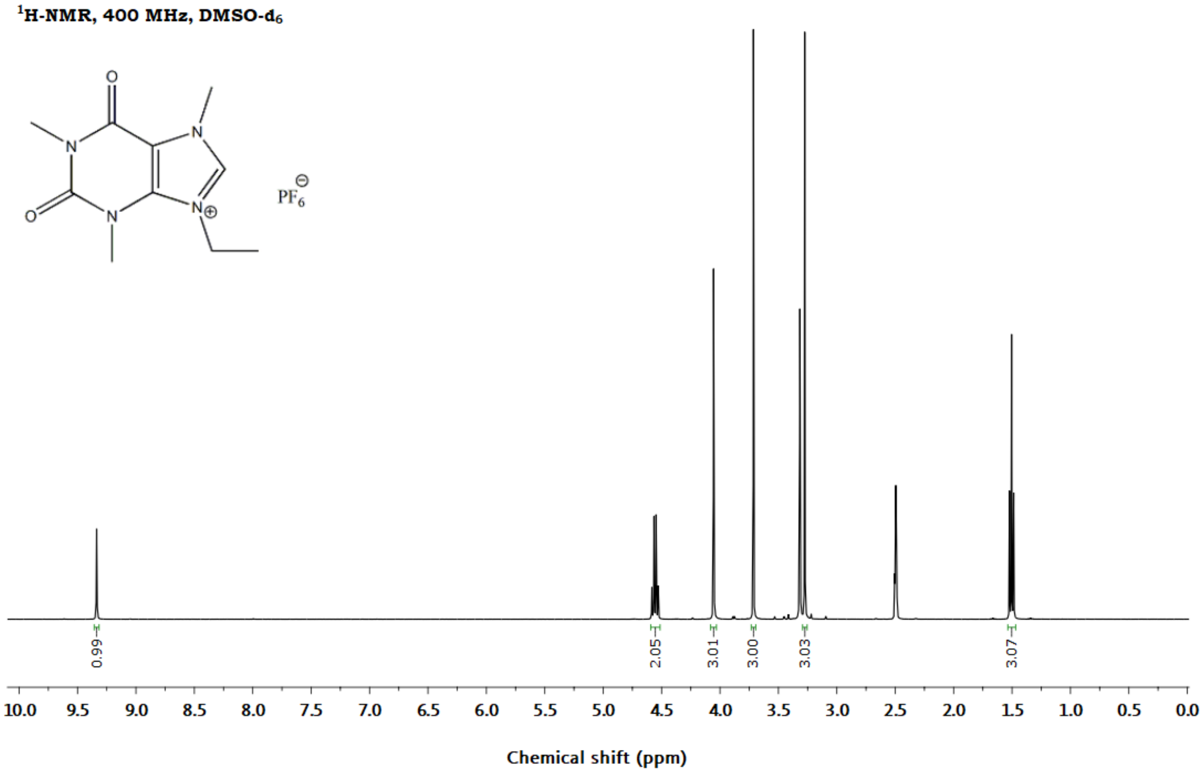

Figure S17

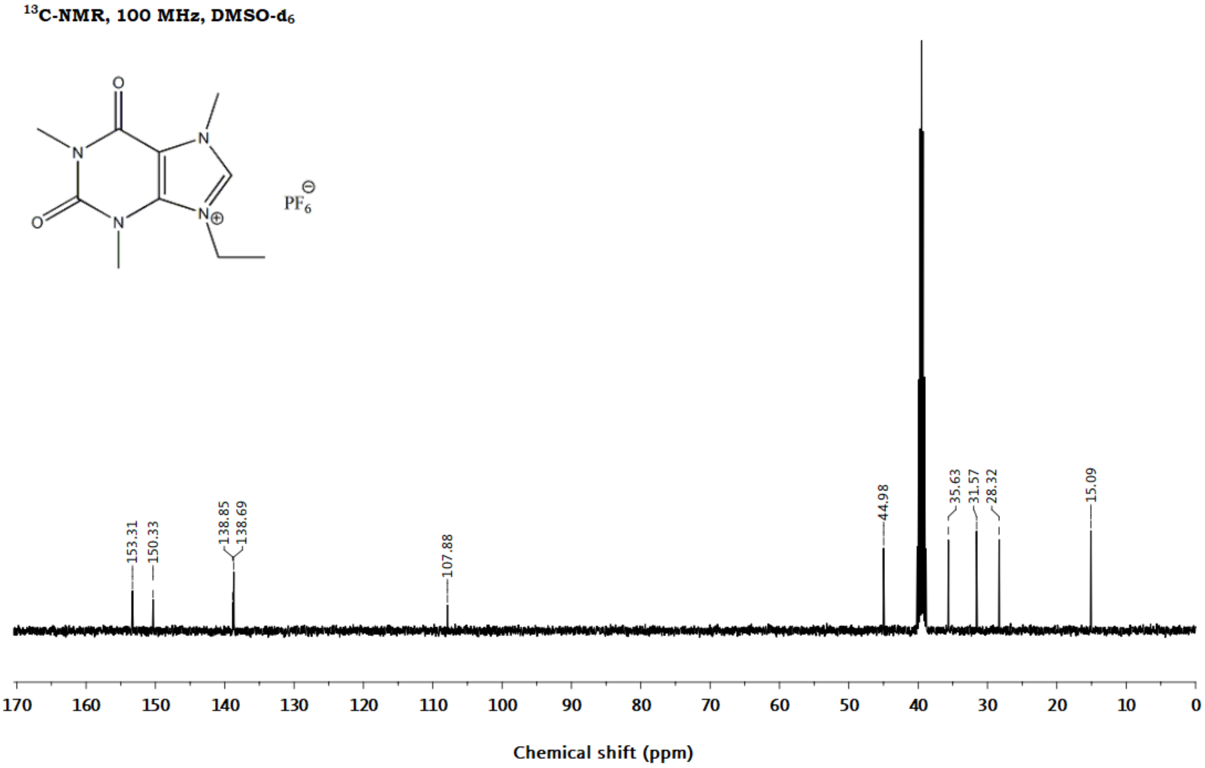

Figure S18

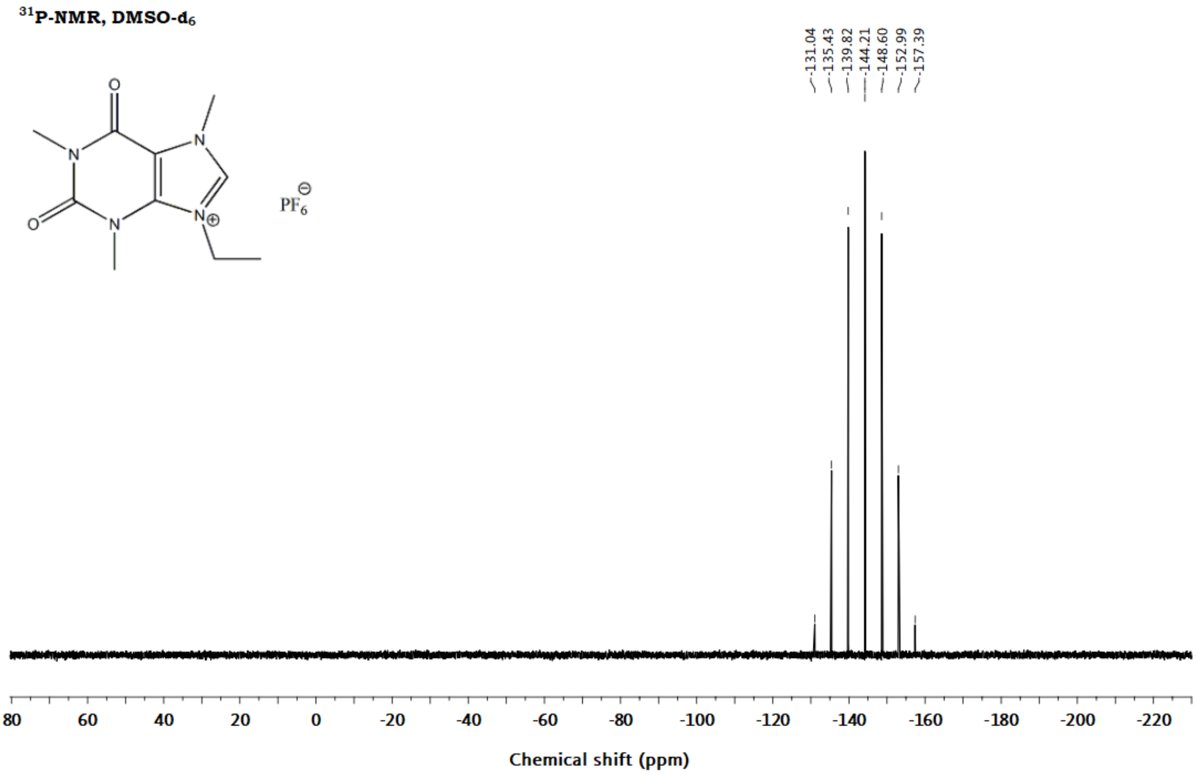

^{31}P-NMR, DMSO-d$_6$
PF$_6$
-131.04
-135.43
-139.82
-144.21
-148.60
-152.99
-157.39
80
60
40
20
0
-20
-40
-60
-80
-100
-120
-140
-160
-180
-200
-220
Chemical shift (ppm)

Figure S19

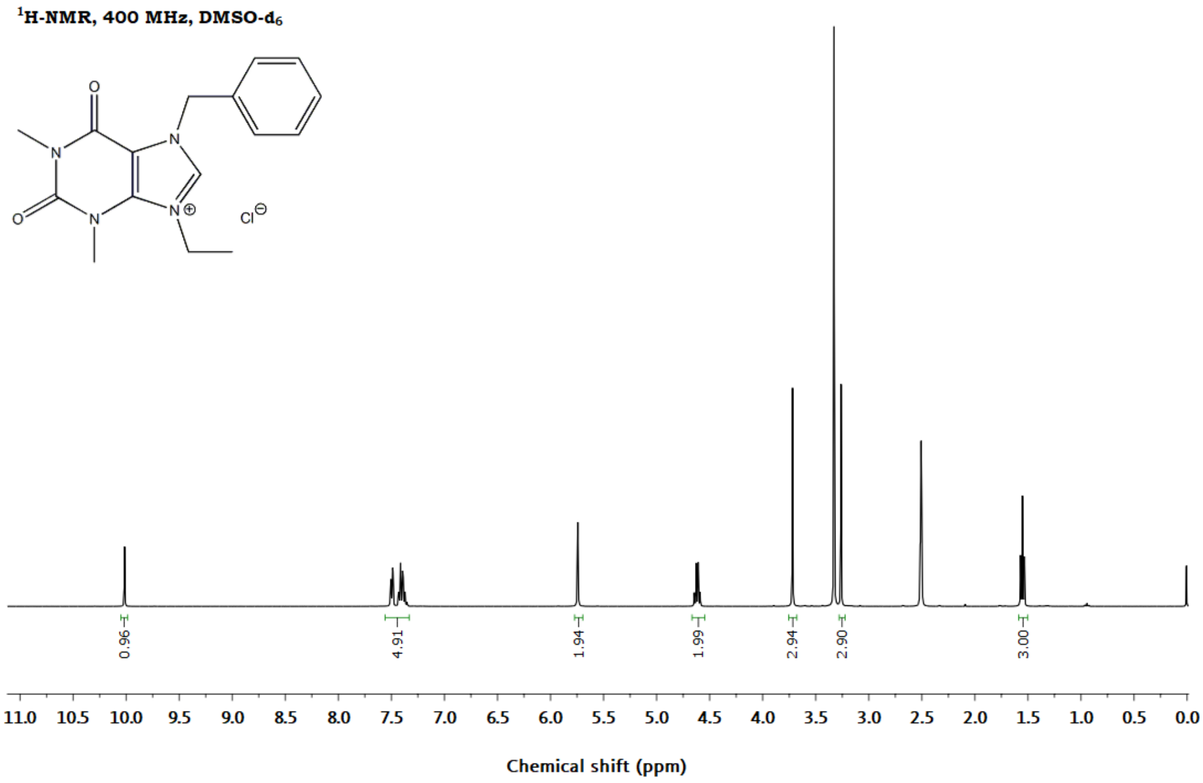

Figure S20

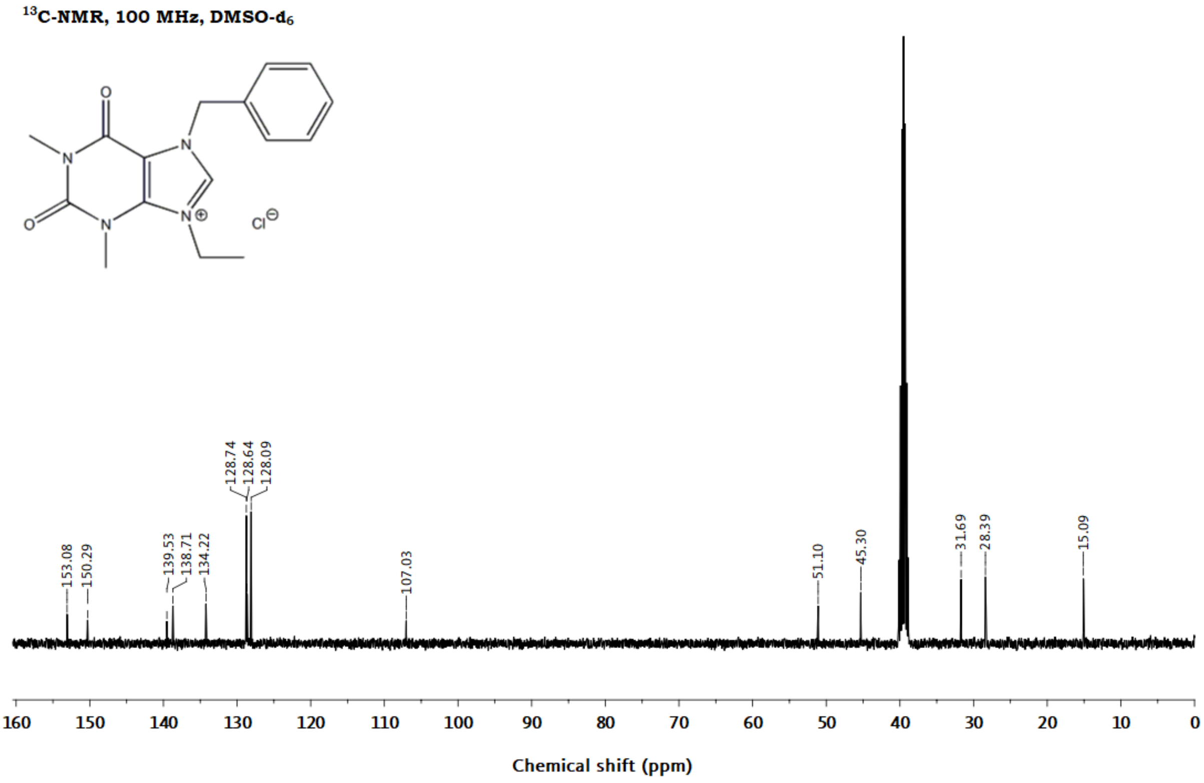

Figure S21

^{35}**Cl-NMR, 54 MHz, DMSO-d$_6$**

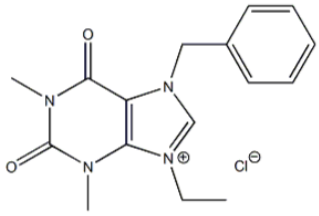

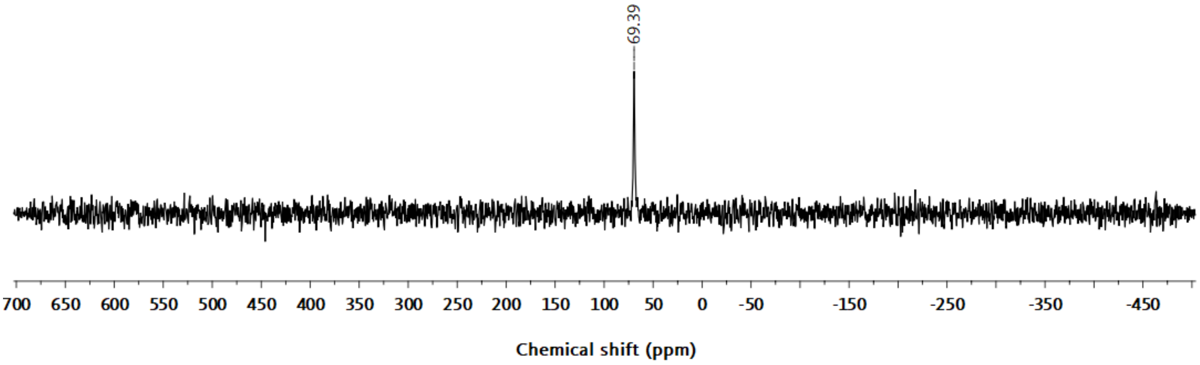

Figure S22

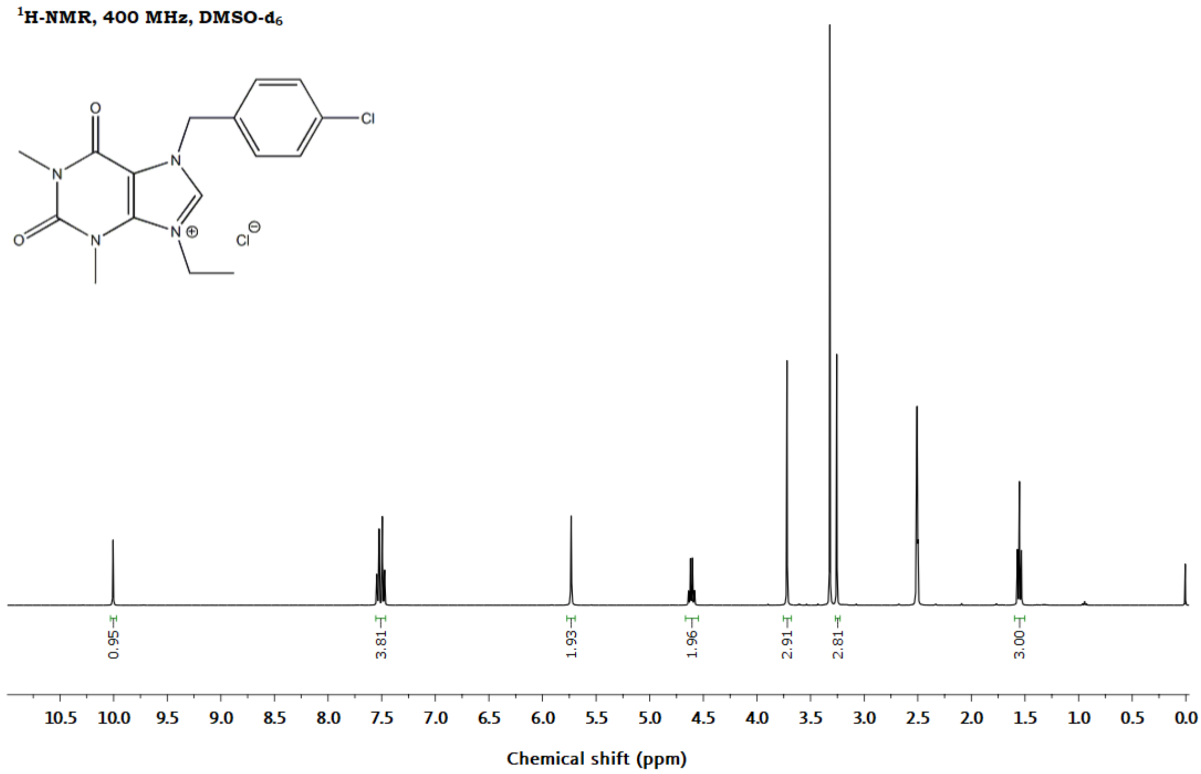

Figure S23

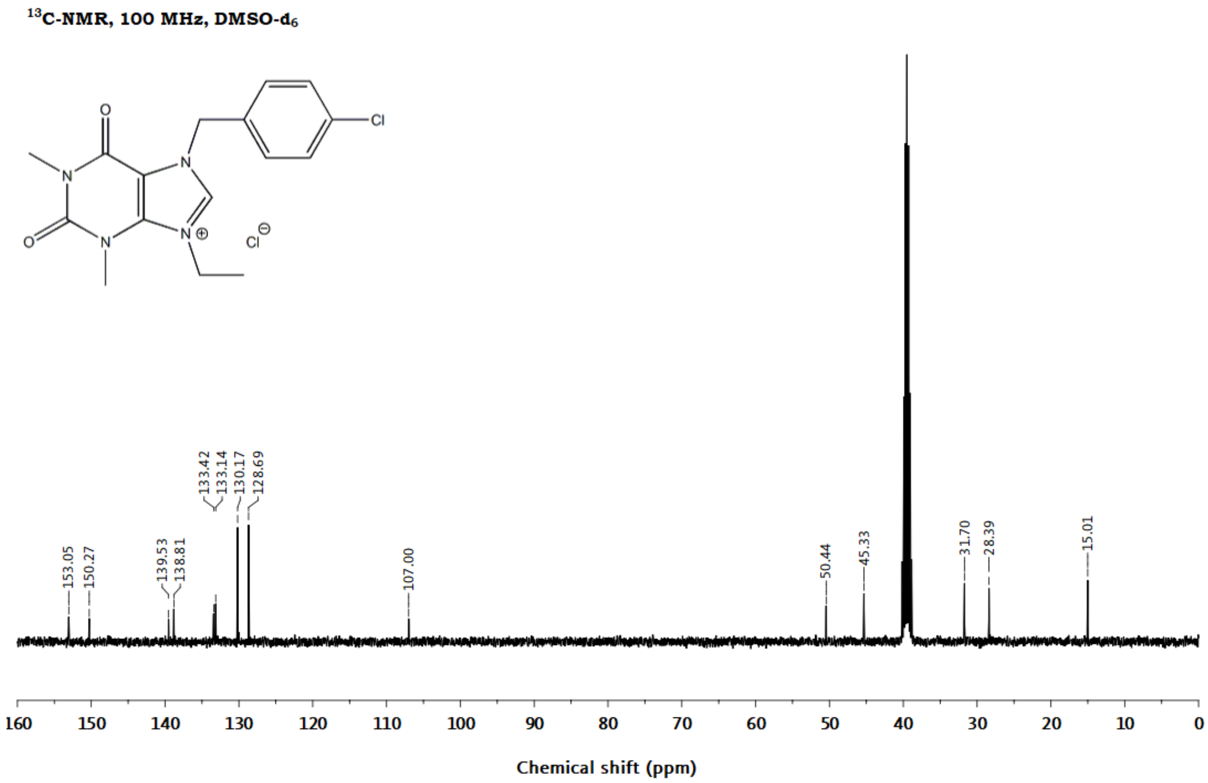

Figure S24

^{35}Cl-NMR, 54 MHz, DMSO-d_6

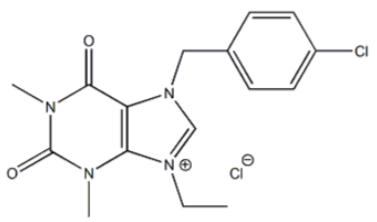

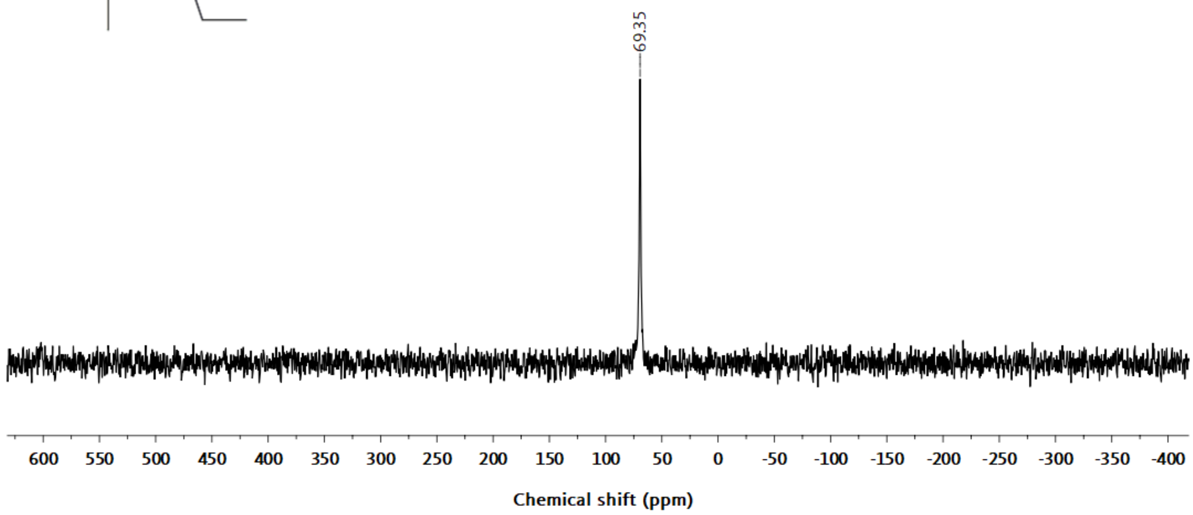

Figure S25

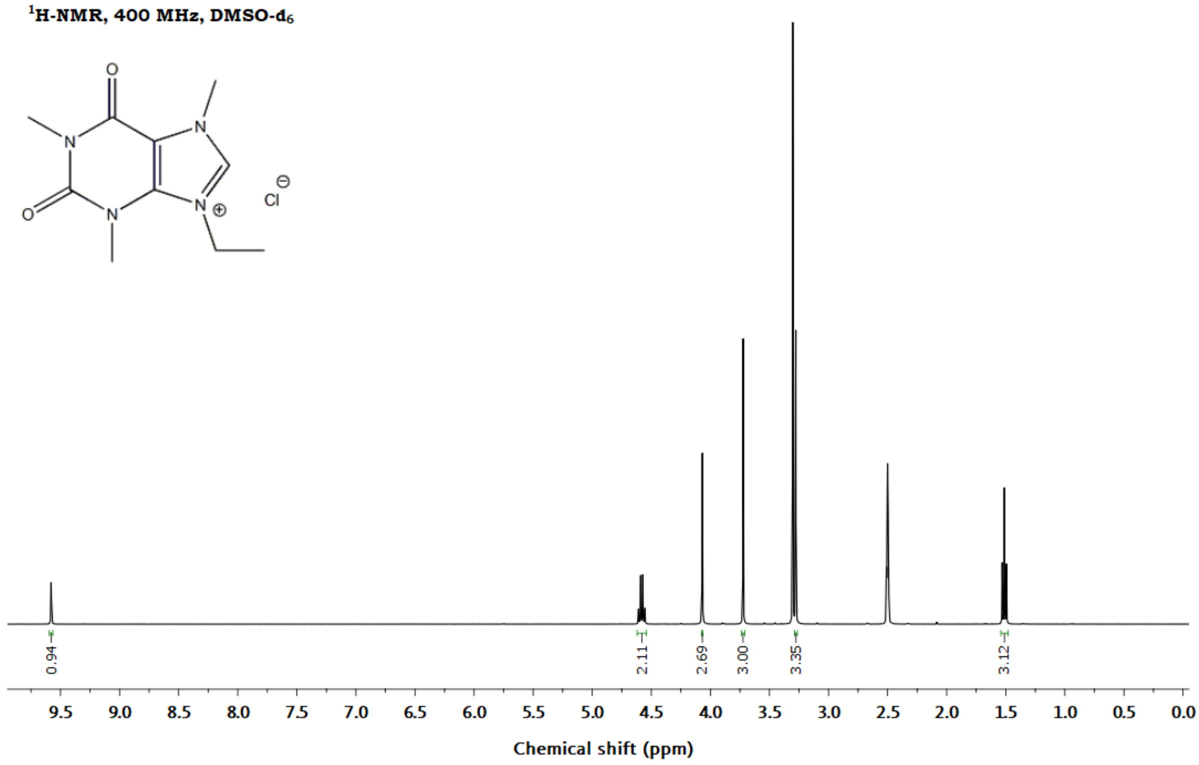

Figure S26

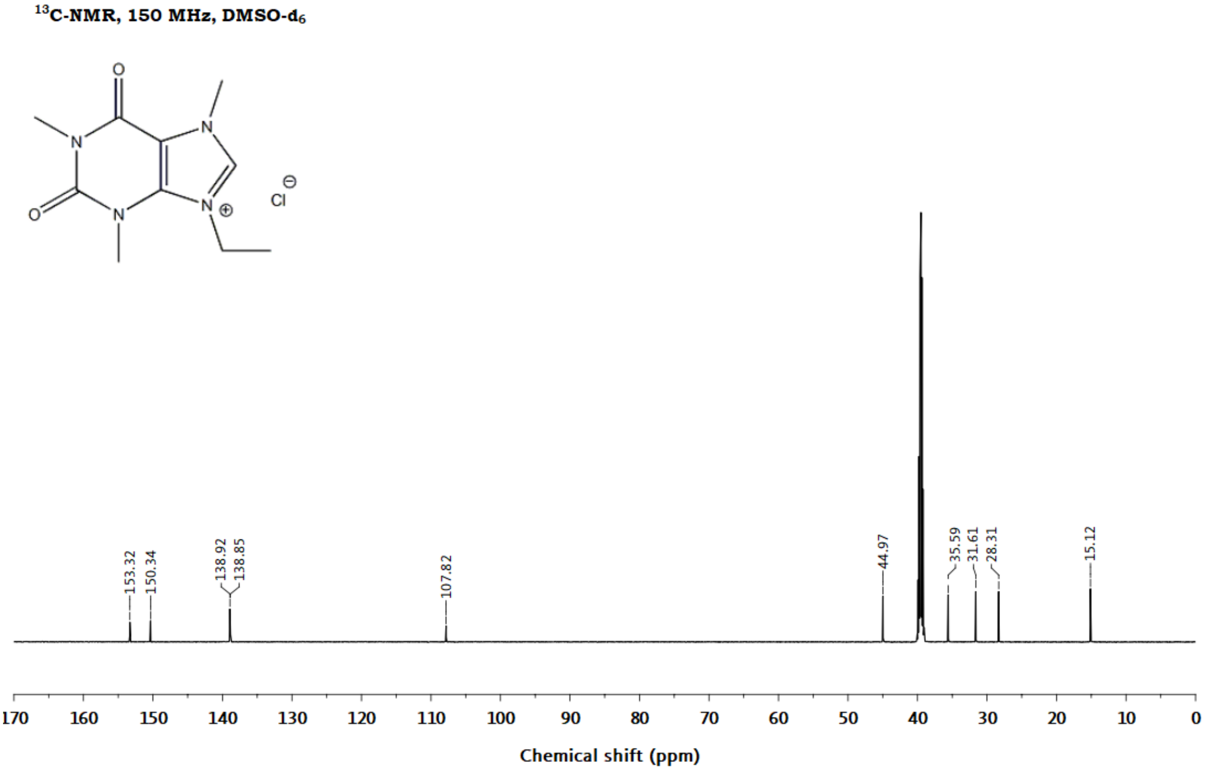

Figure S27

^{35}**Cl-NMR, 39 MHz, DMSO-d$_6$**

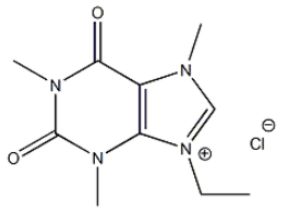

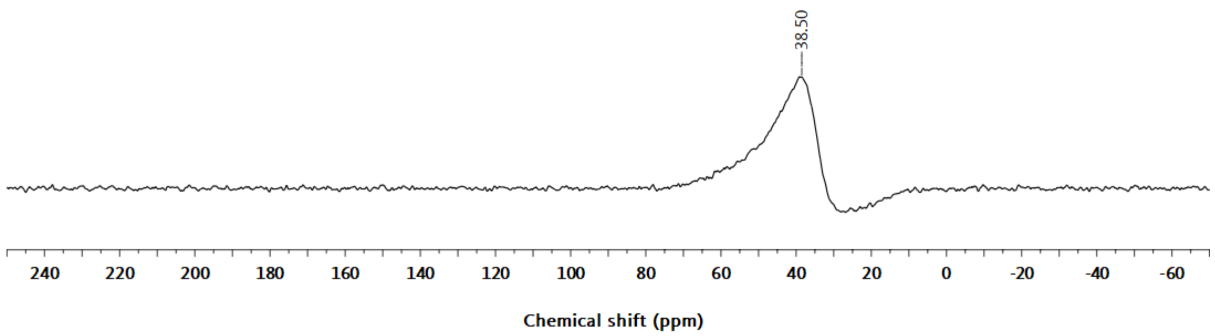

Figure S 28

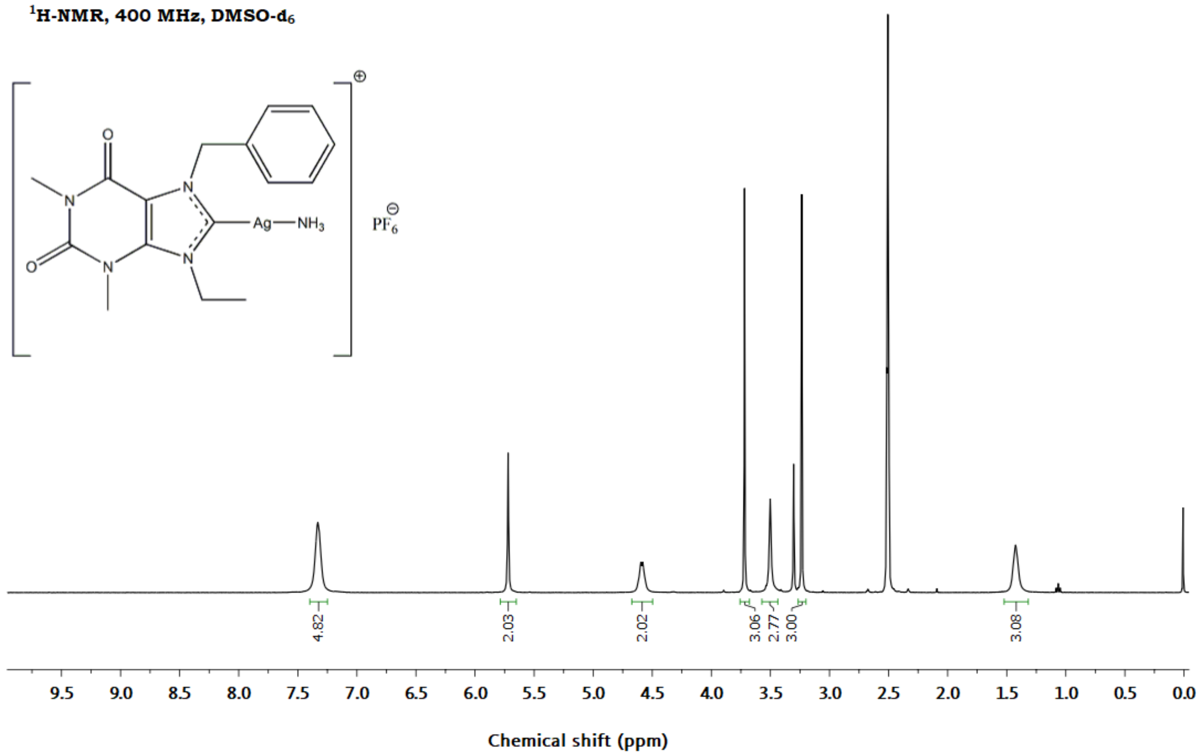

Figure S29

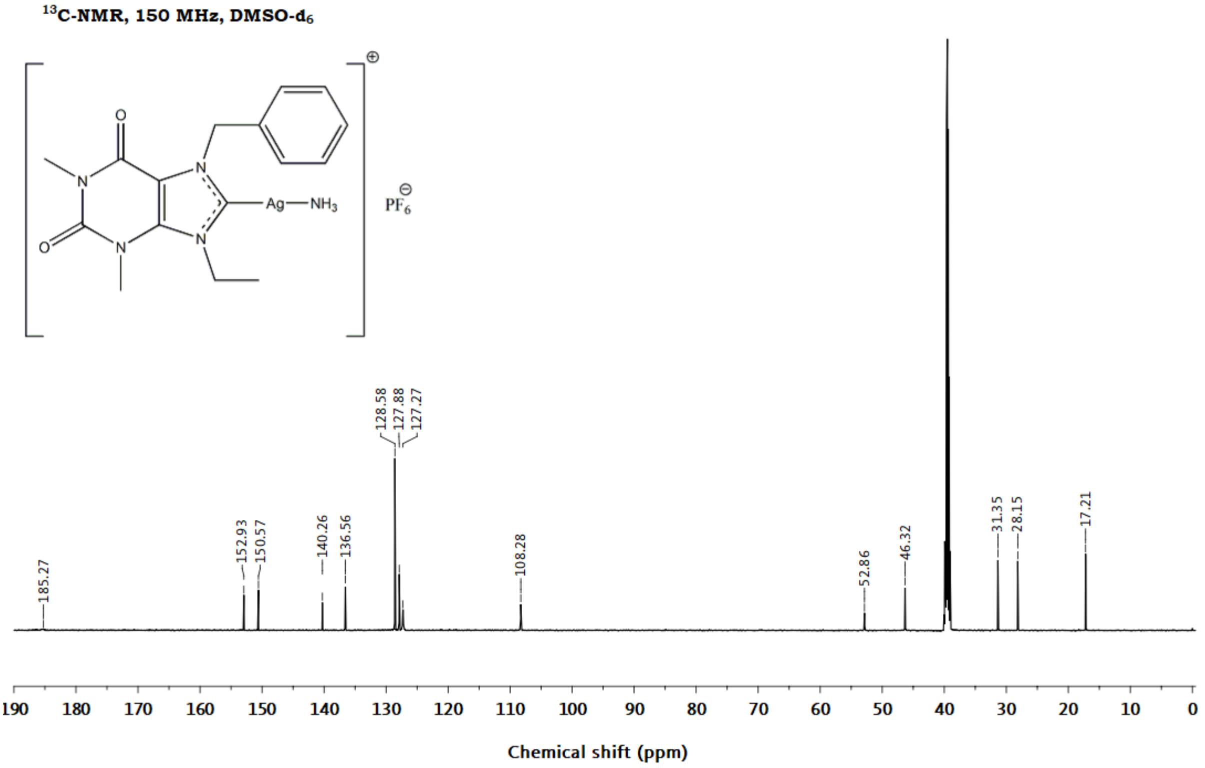

Figure S30

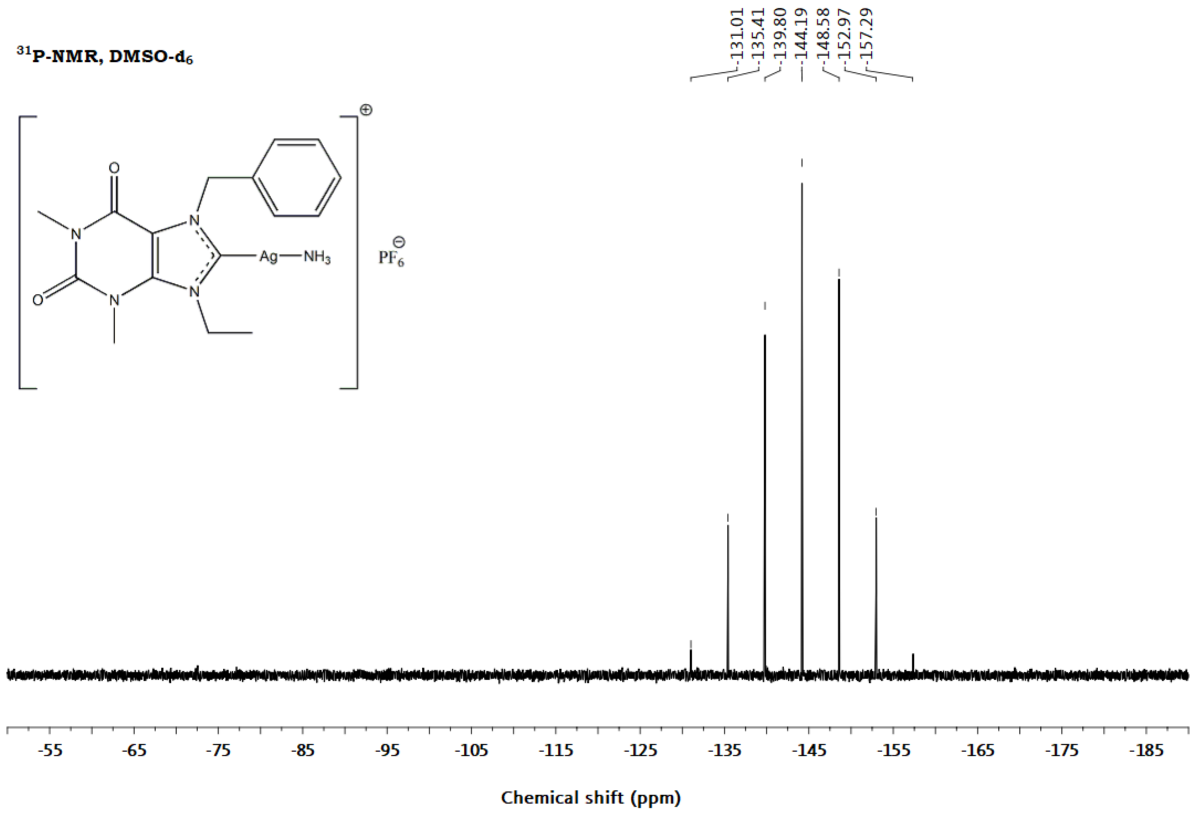

31P-NMR, DMSO-d6
-131.01
-135.41
-139.80
-144.19
-148.58
-152.97
-157.29
O
N
N
O
N
N
Ag
NH3
PF6
-55
-65
-75
-85
-95
-105
-115
-125
-135
-145
-155
-165
-175
-185
Chemical shift (ppm)

Figure S31

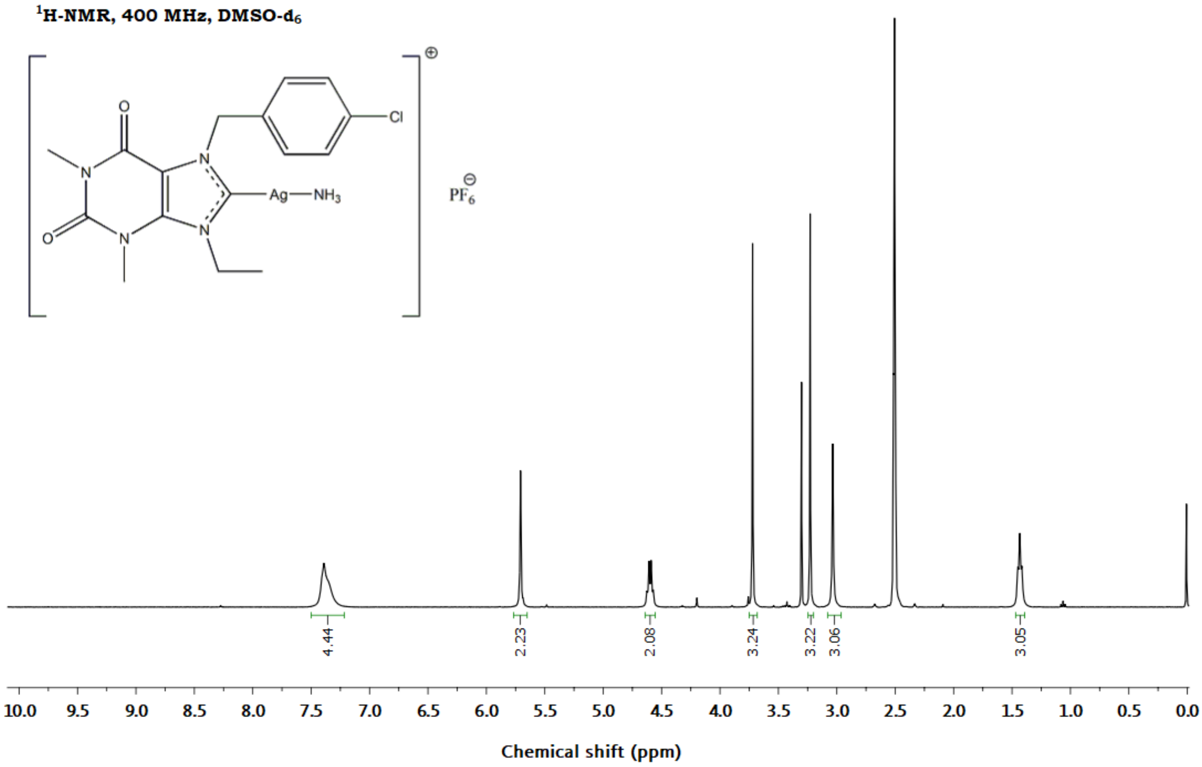

Figure S32

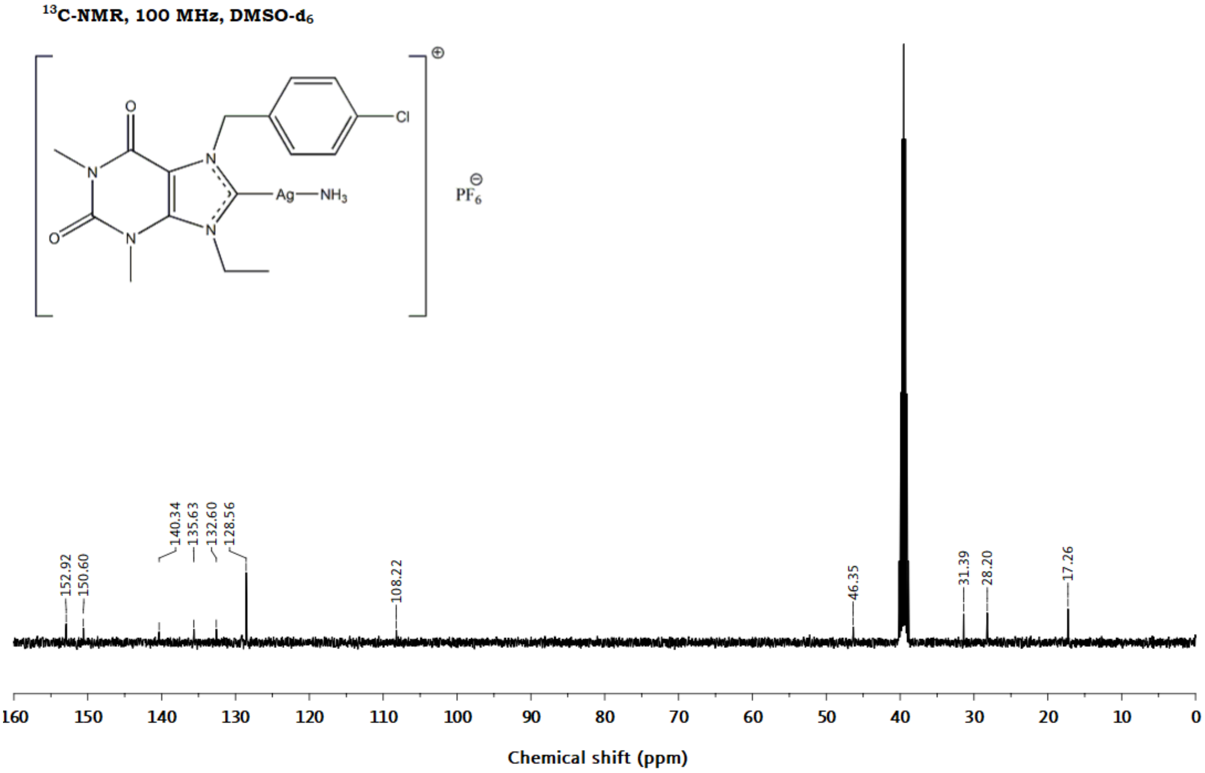

Figure S33

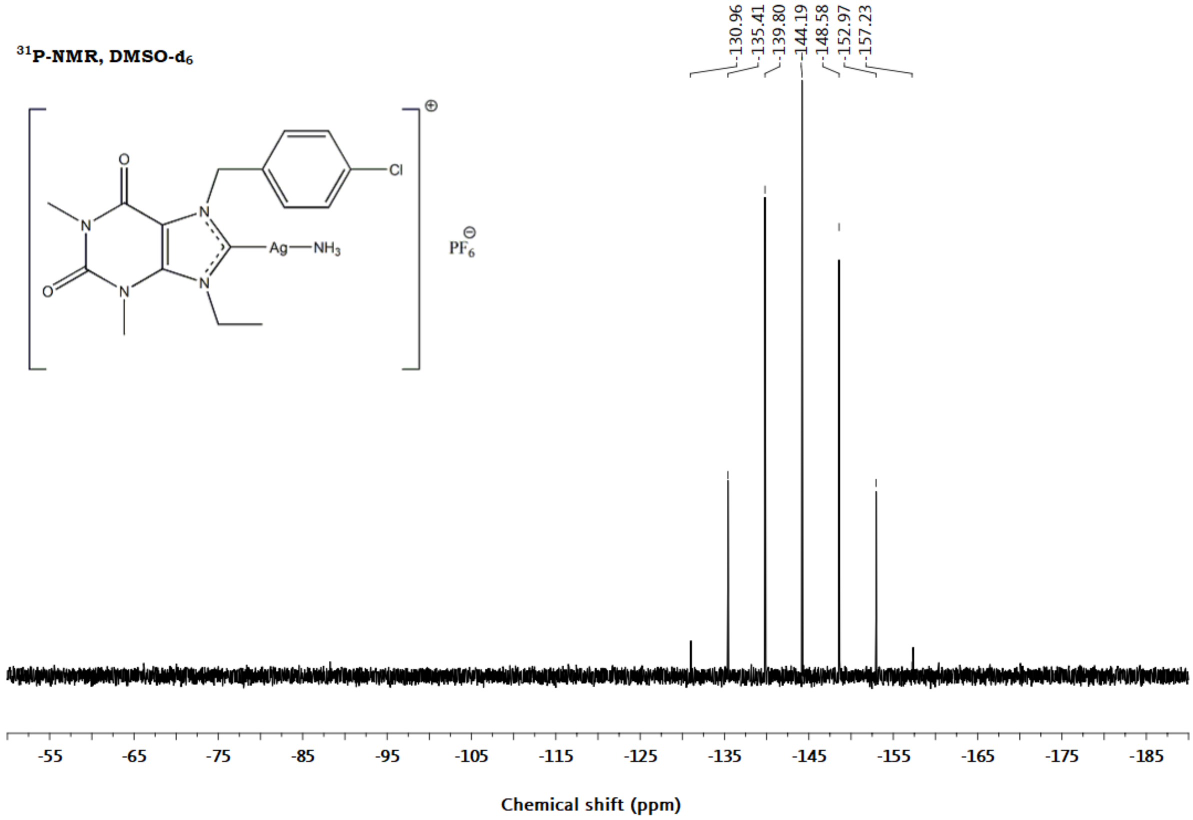

31P-NMR, DMSO-d6
Ag
NH3
Cl
PF6
130.96
135.41
139.80
144.19
148.58
152.97
157.23
-55
-65
-75
-85
-95
-105
-115
-125
-135
-145
-155
-165
-175
-185
Chemical shift (ppm)

Figure S34

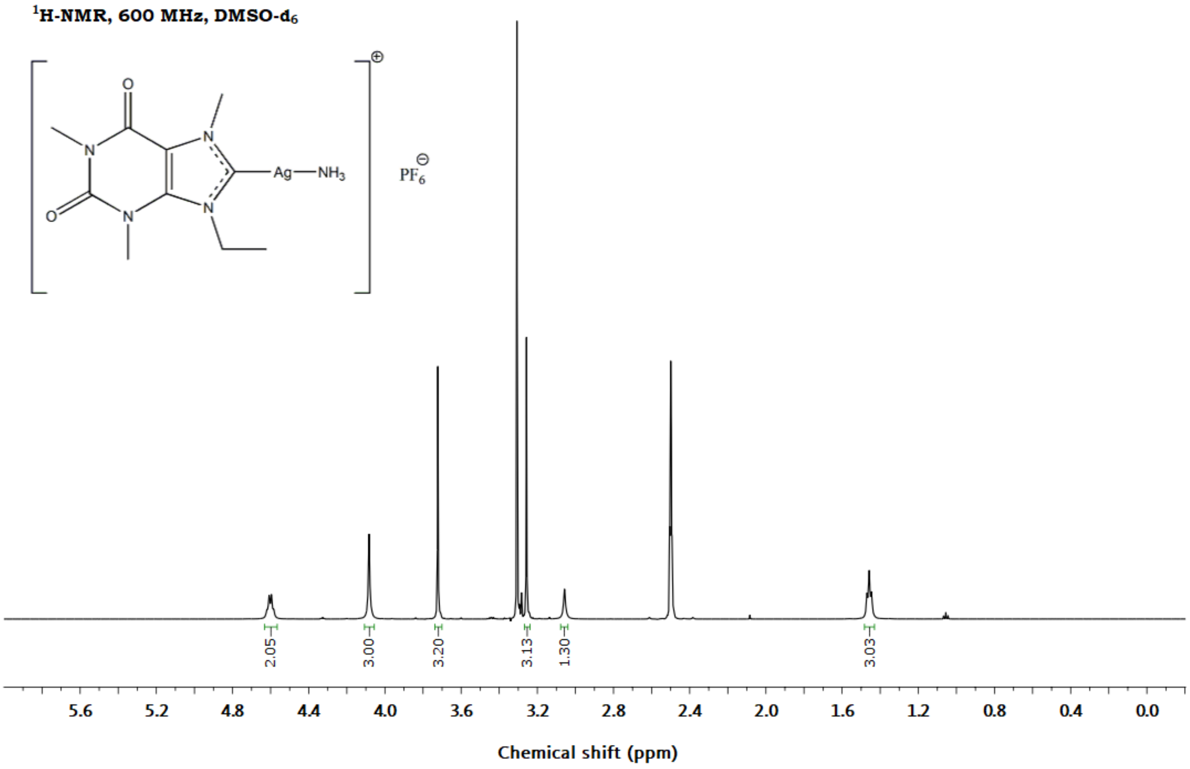

Figure S35

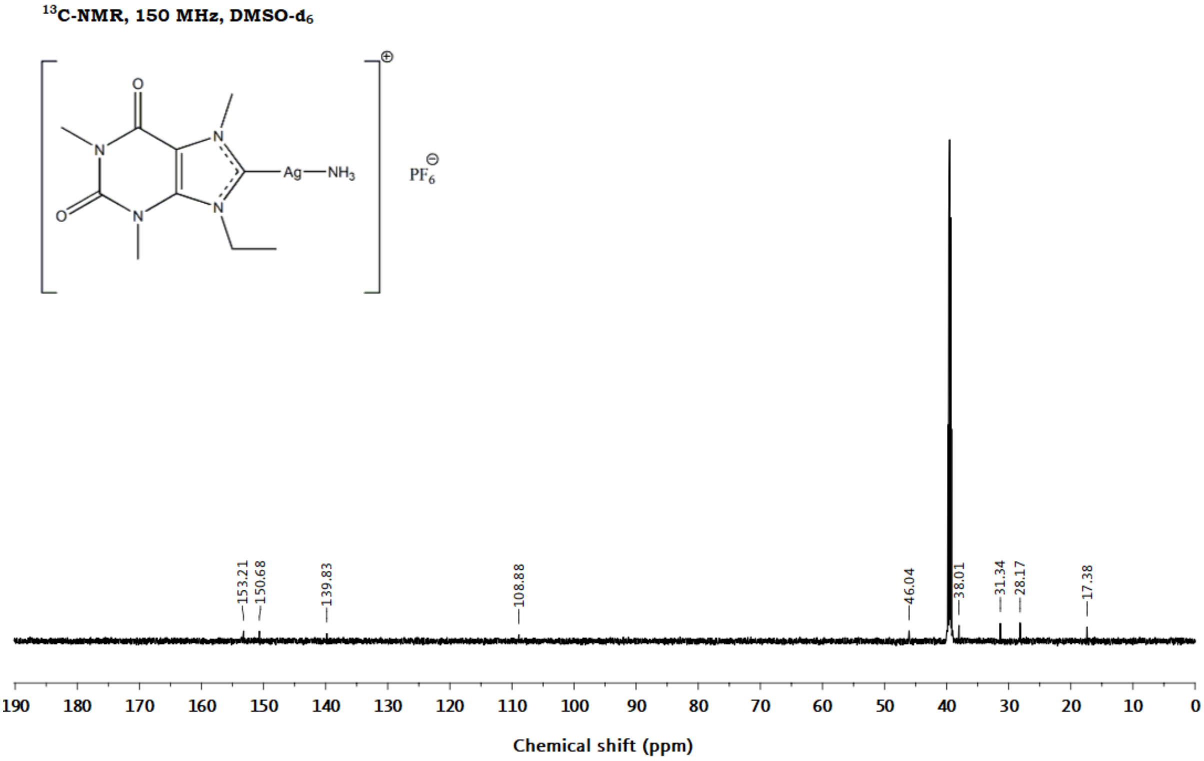

Figure S36

^{1}H-NMR, 600 MHz, DMSO-d_6

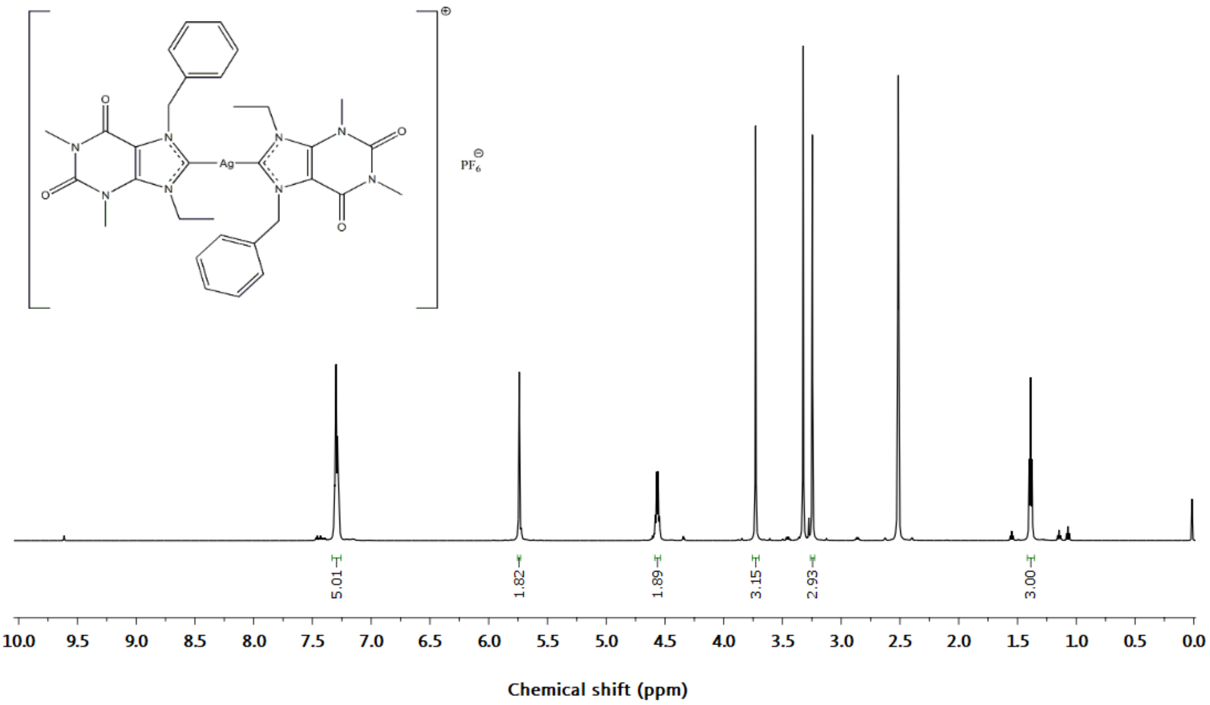

Figure S37

^{13}C-NMR, 150 MHz, DMSO-d_6

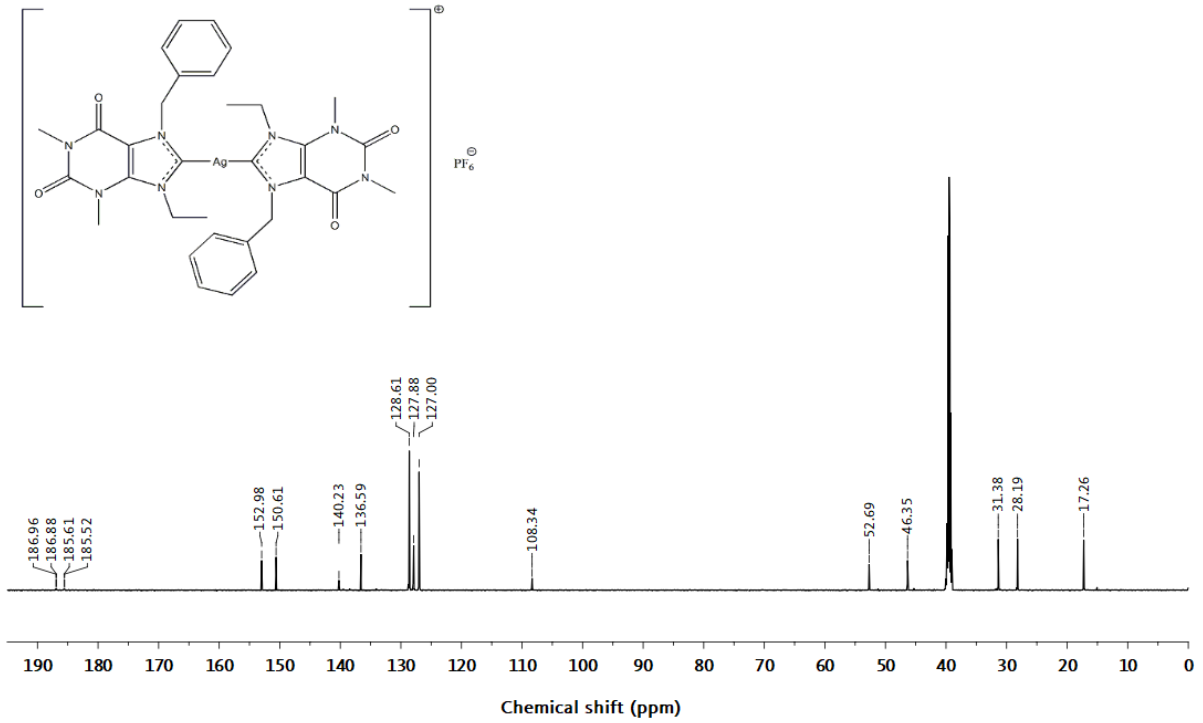

Figure S 38

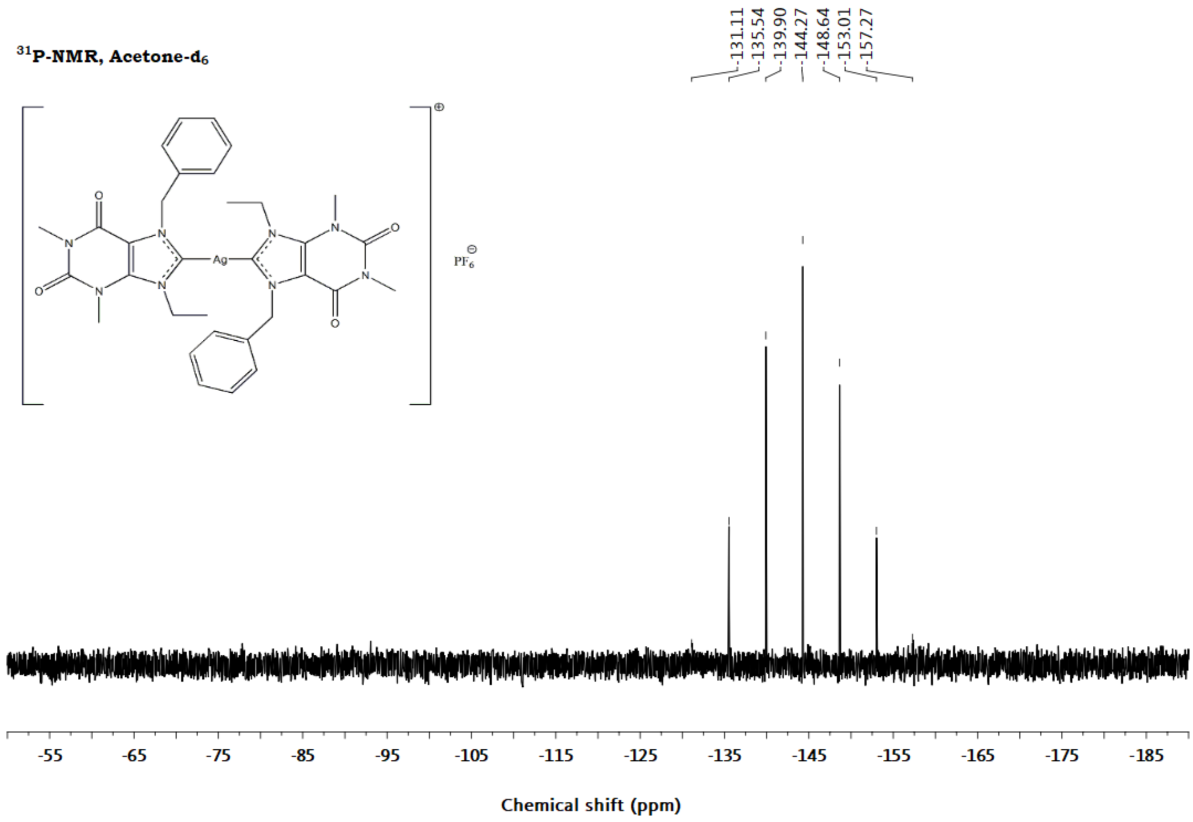

^{31}P-NMR, Acetone-d$_6$
-131.11
-135.54
-139.90
-144.27
-148.64
-153.01
-157.27
N
Ag
O
PF$_6$
-55
-65
-75
-85
-95
-105
-115
-125
-135
-145
-155
-165
-175
-185
Chemical shift (ppm)

Figure S39

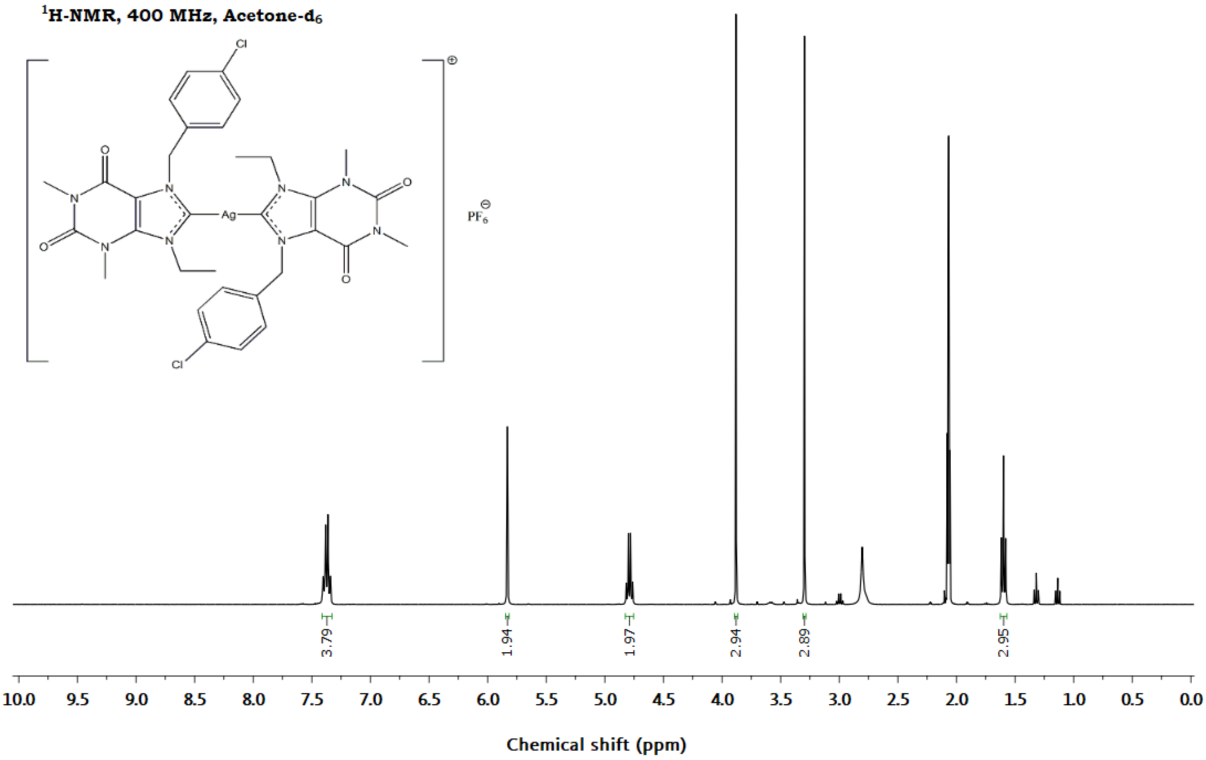

Figure S40

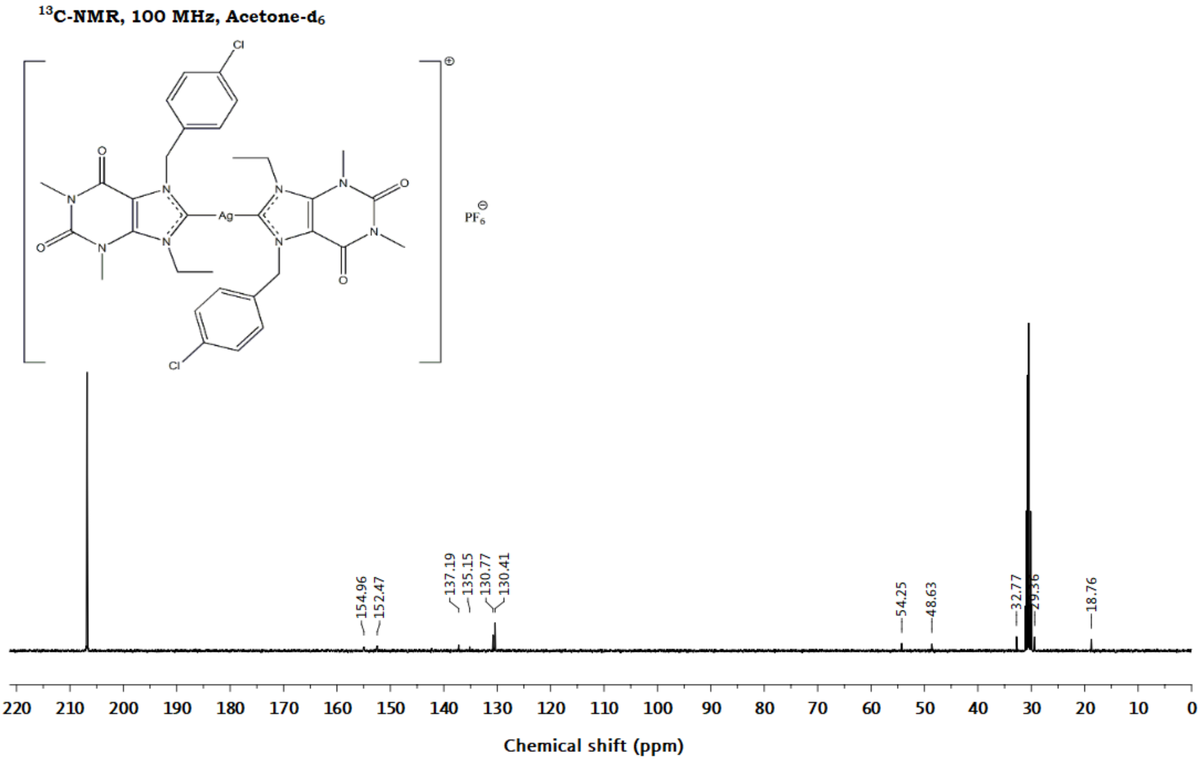

Figure S41

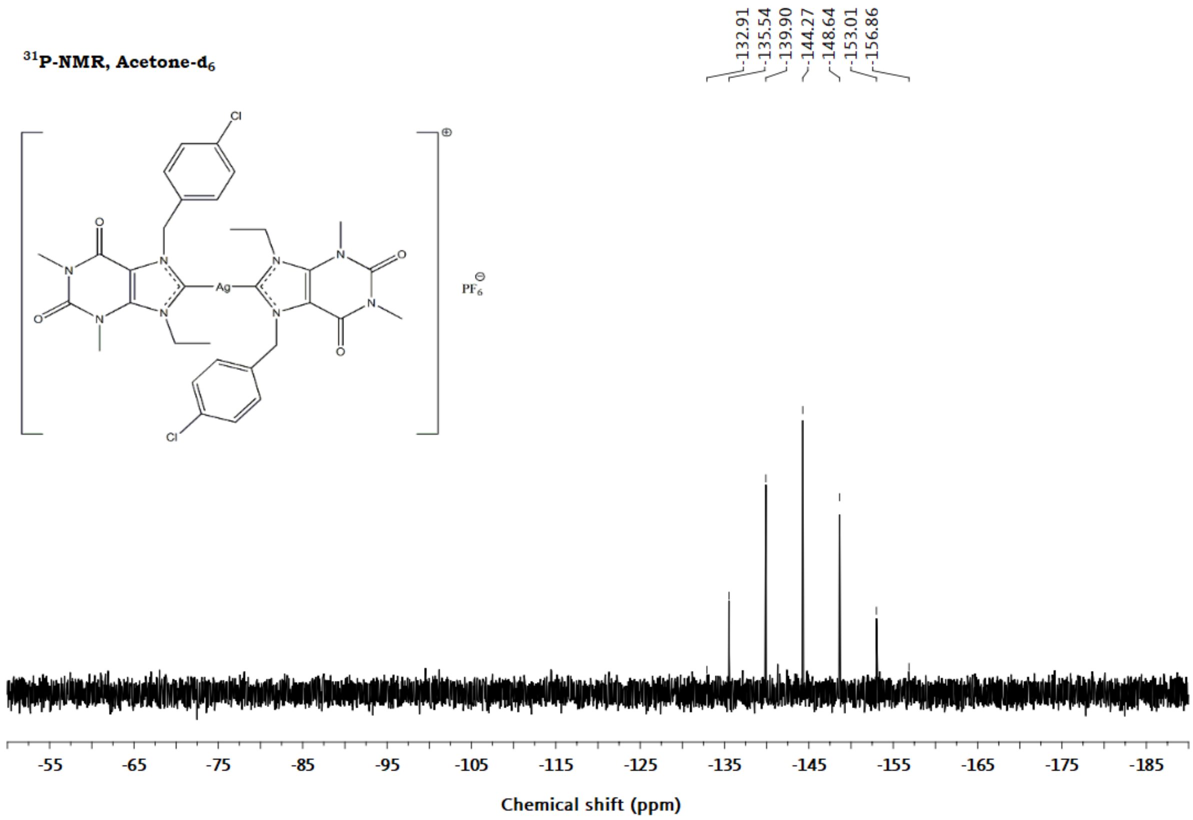

^{31}P-NMR, Acetone-d$_6$
-132.91
-135.54
-139.90
-144.27
-148.64
-153.01
-156.86
Cl
O
N
Ag
PF$_6$
-55
-65
-75
-85
-95
-105
-115
-125
-135
-145
-155
-165
-175
-185
Chemical shift (ppm)

Figure S42

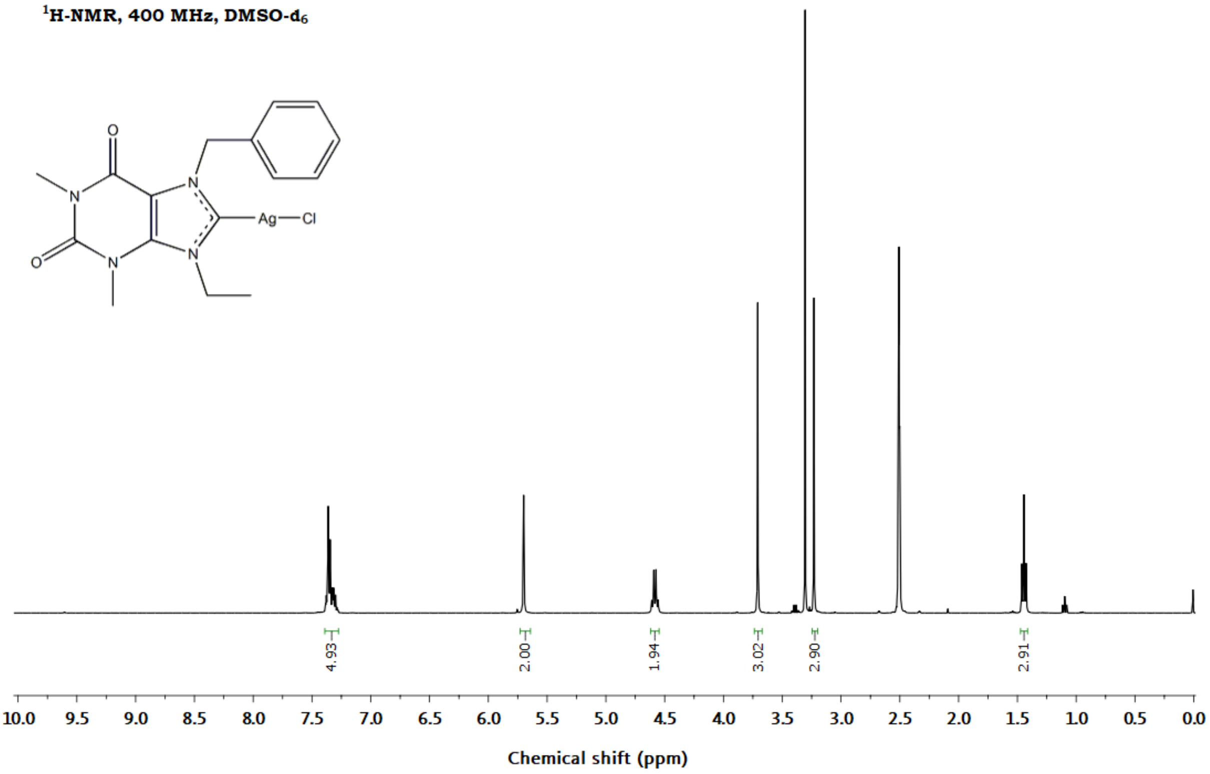

Figure S43

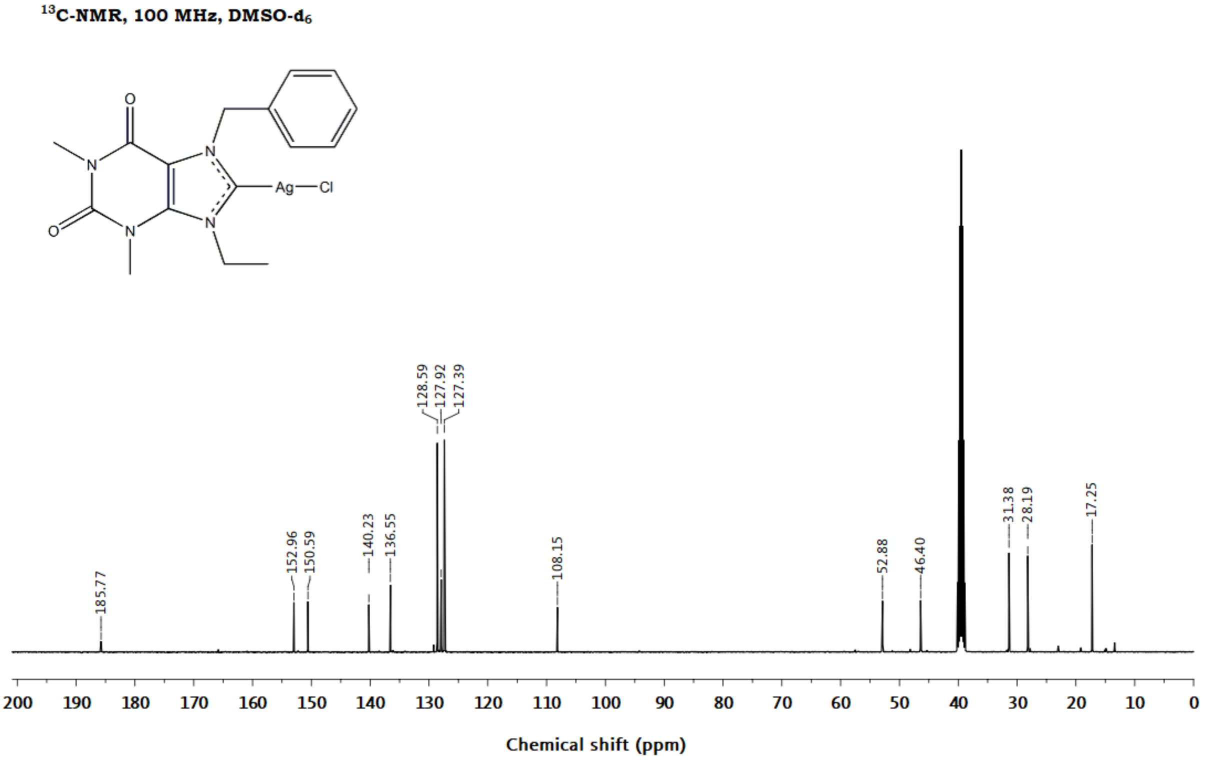

Figure S44

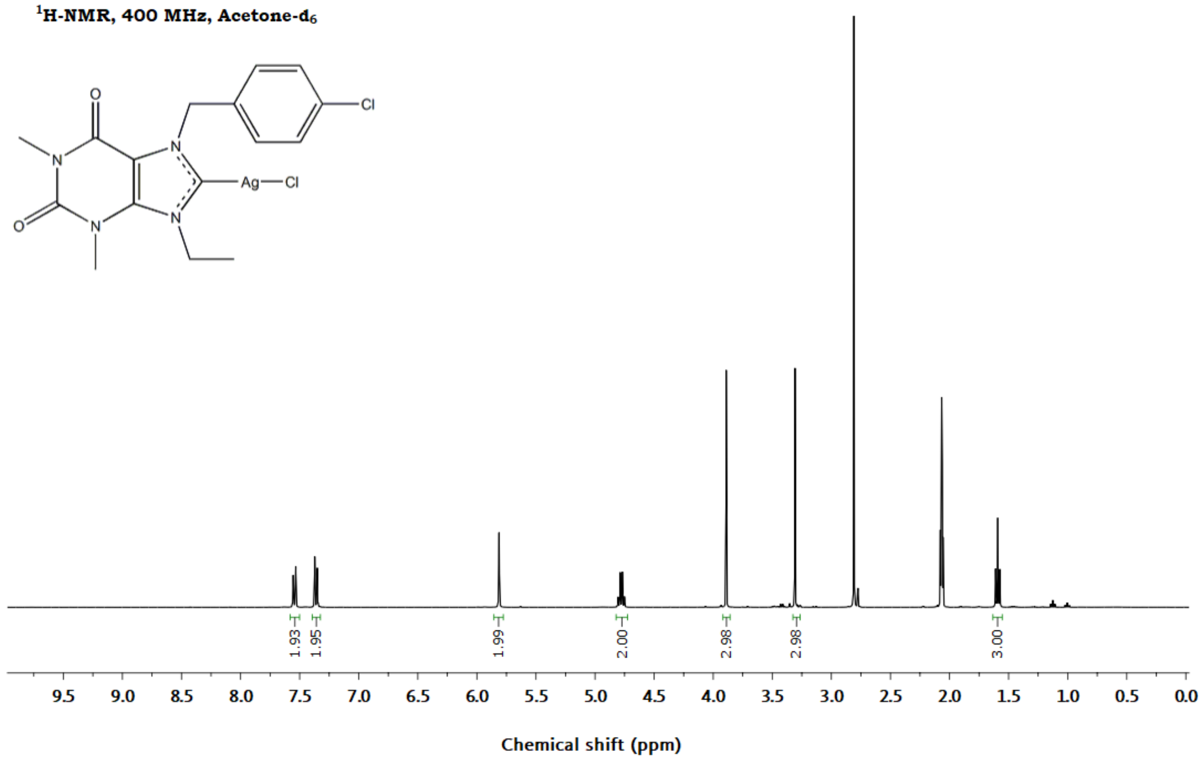

Figure S45

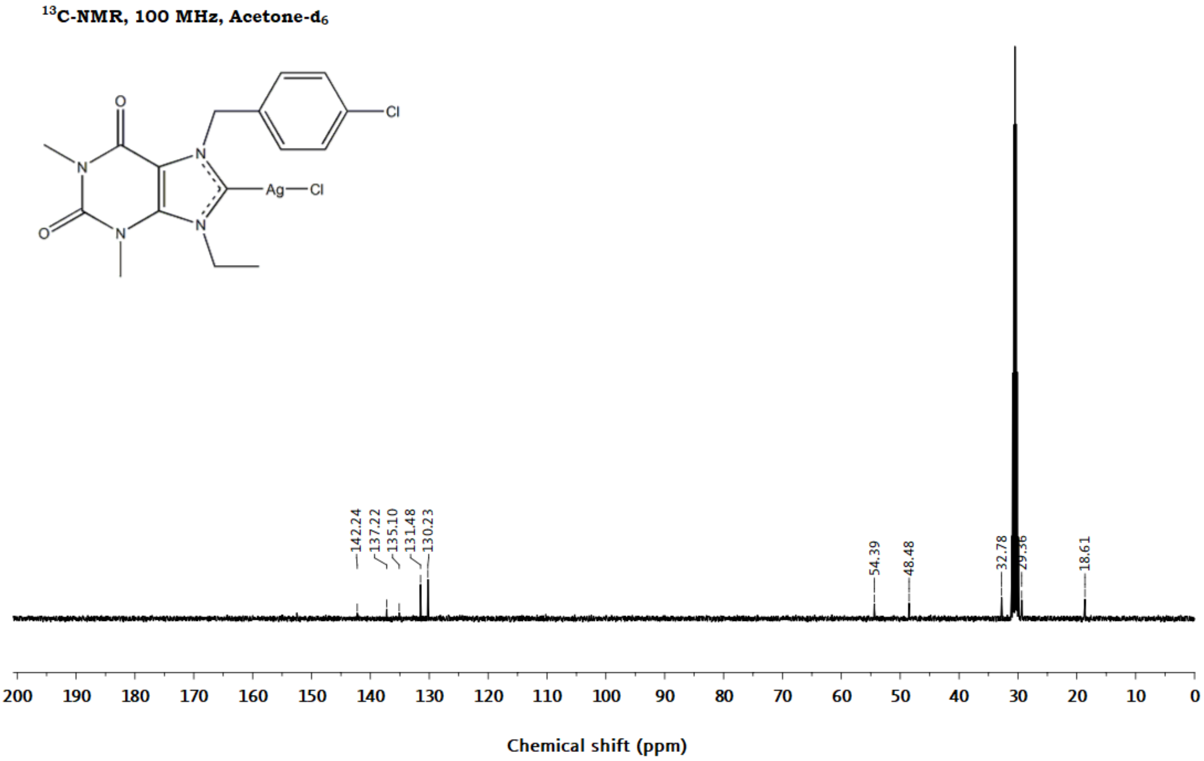

Figure S46

^{1}H-NMR, 400 MHz, CDCl$_3$, 223 K

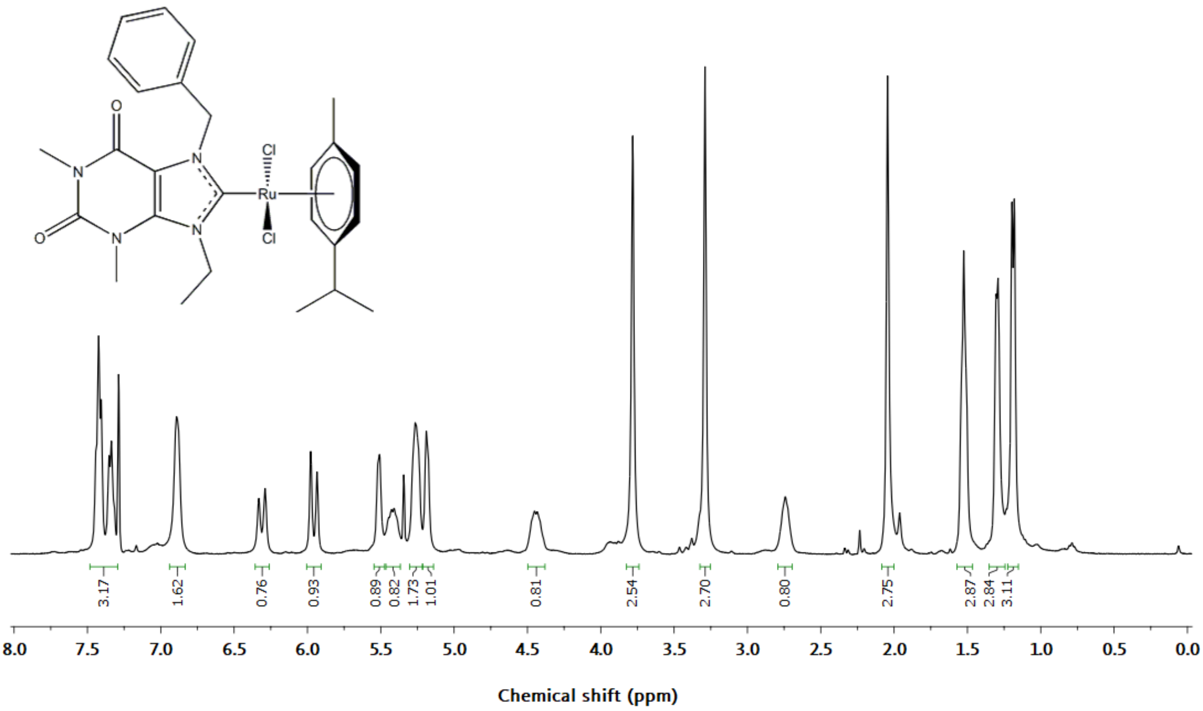

Figure S47

^{13}C-NMR, 100 MHz, CDCl$_3$, 223 K

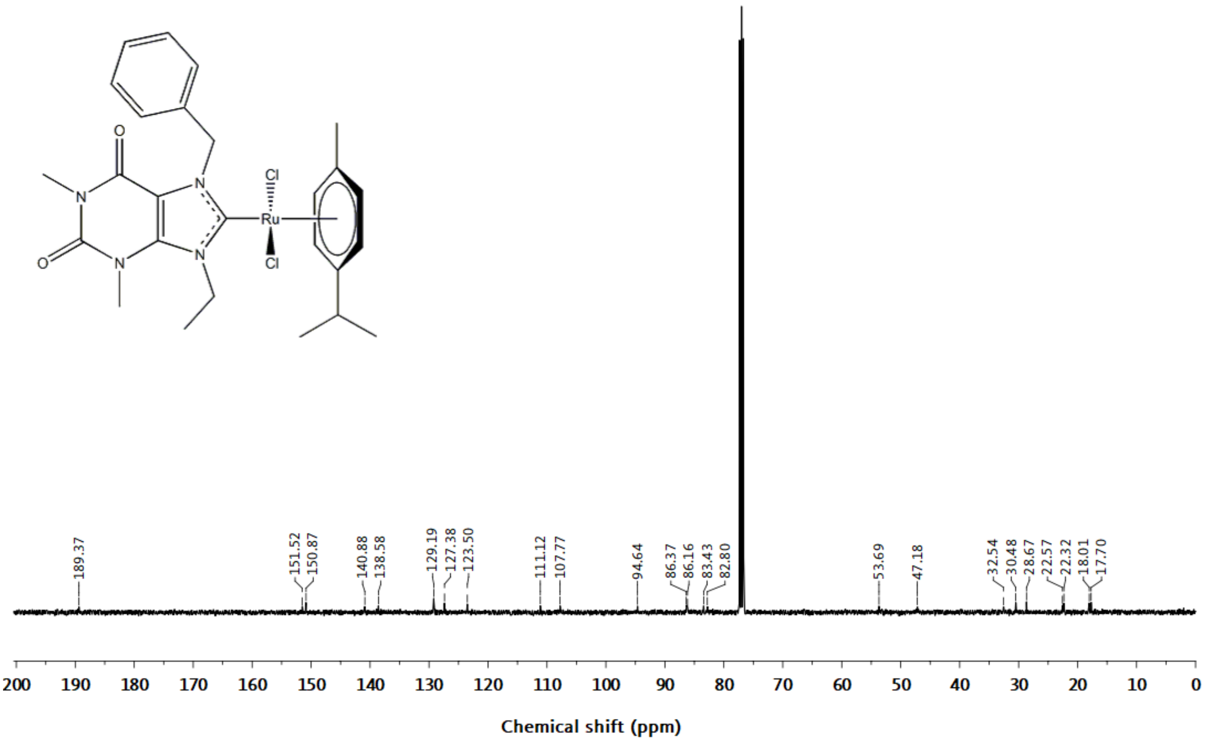

Figure S48

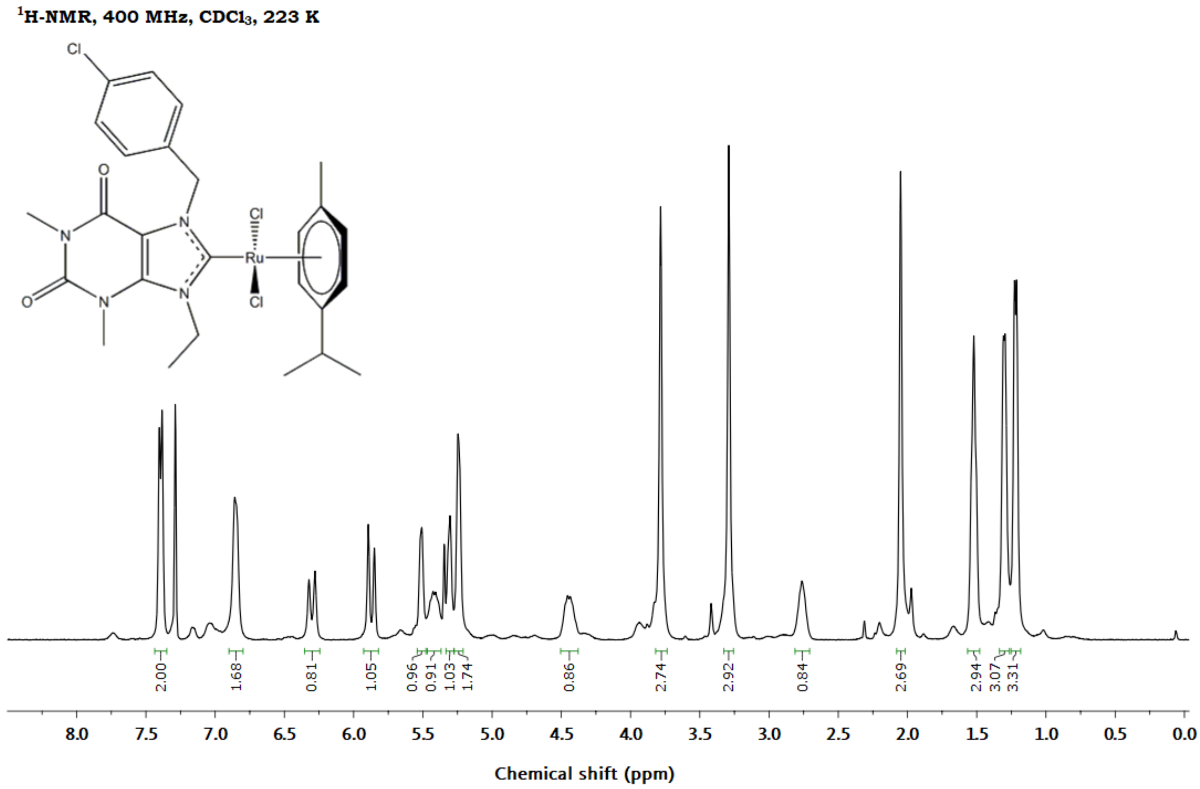

Figure S49

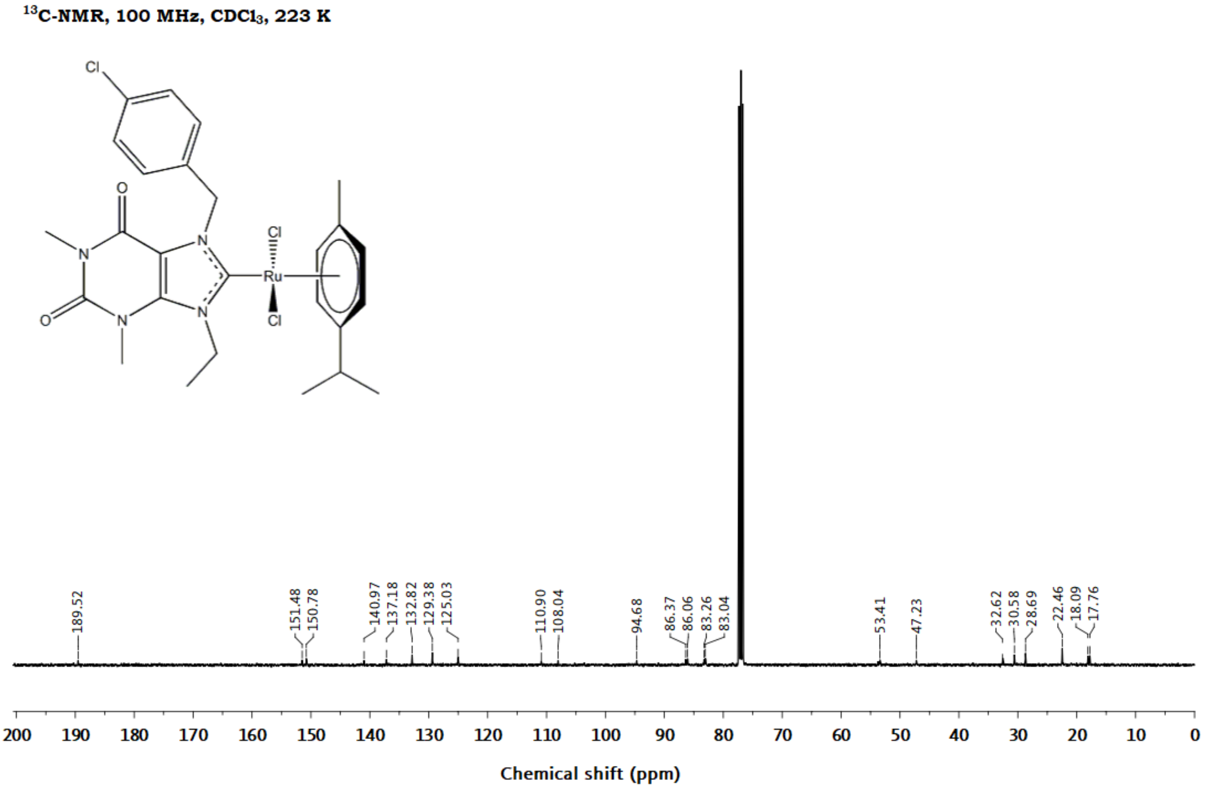

Figure S50

^{1}H-NMR, 600 MHz, $CDCl_3$

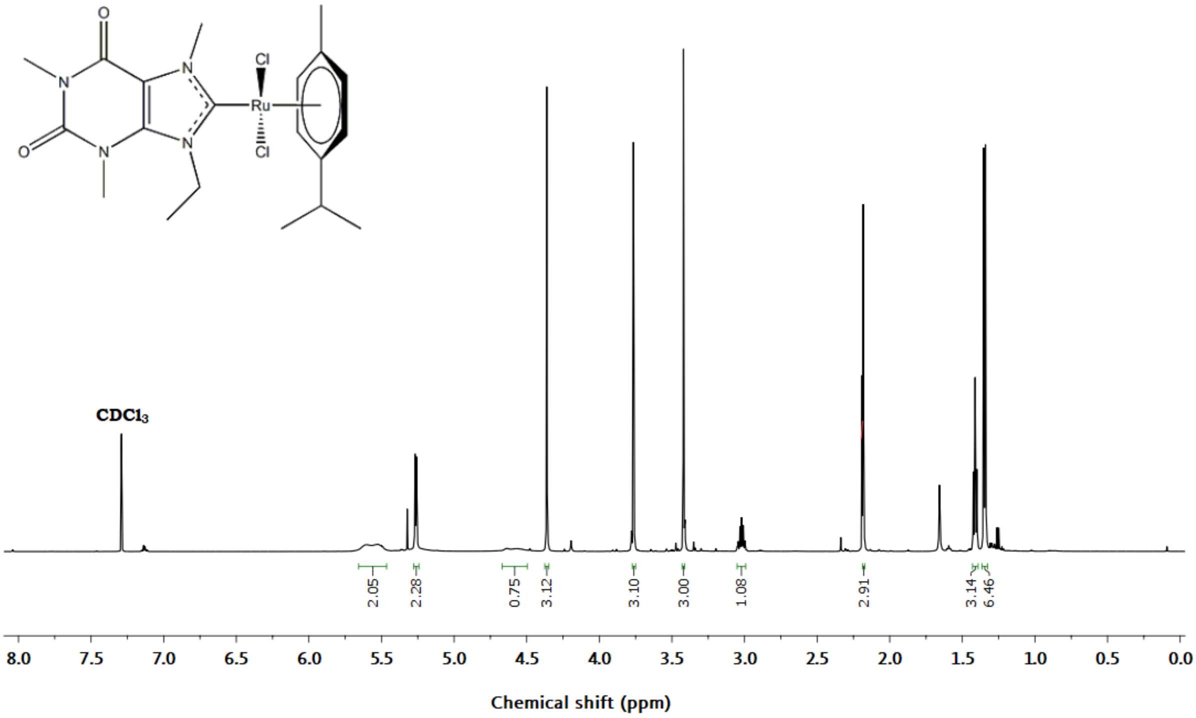

Figure S51

^{13}C-NMR, 150 MHz, $CDCl_3$

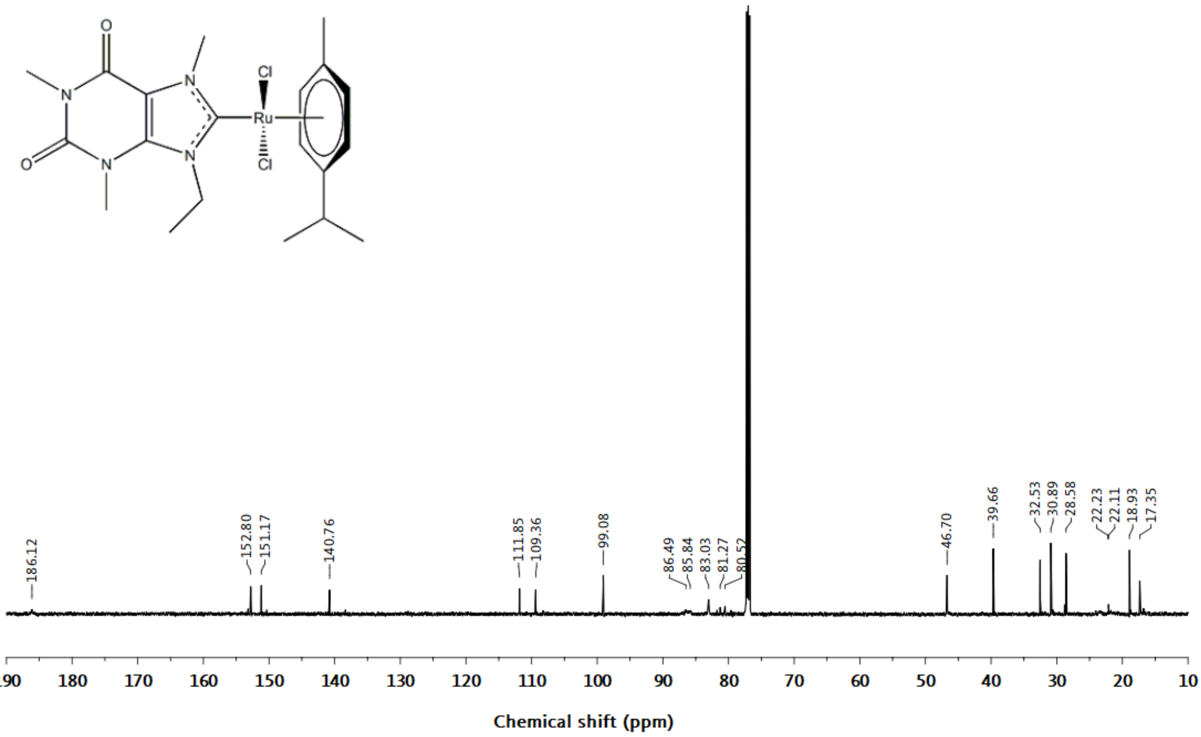

Figure S52

^{1}H-NMR, 400 MHz, $CDCl_3$

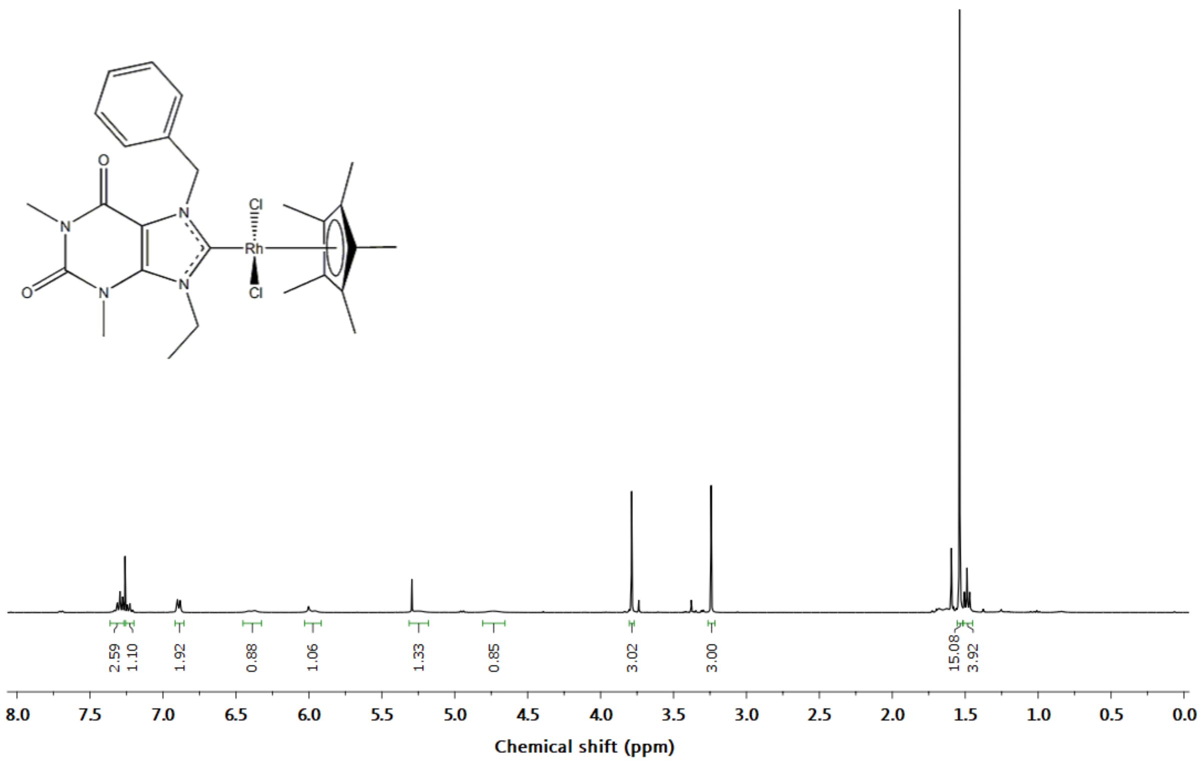

Figure S53

^{13}C-NMR, 150 MHz, $CDCl_3$

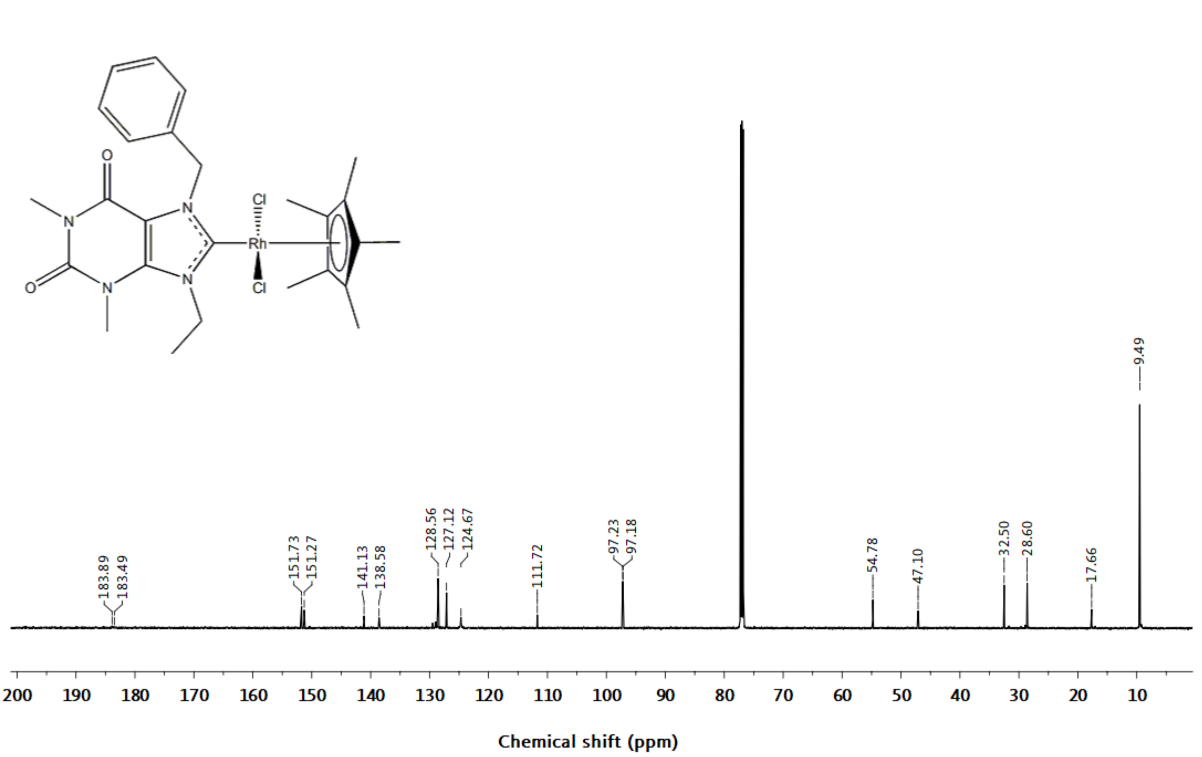

Figure S54

^{1}H-NMR, 400 MHz, $CDCl_3$

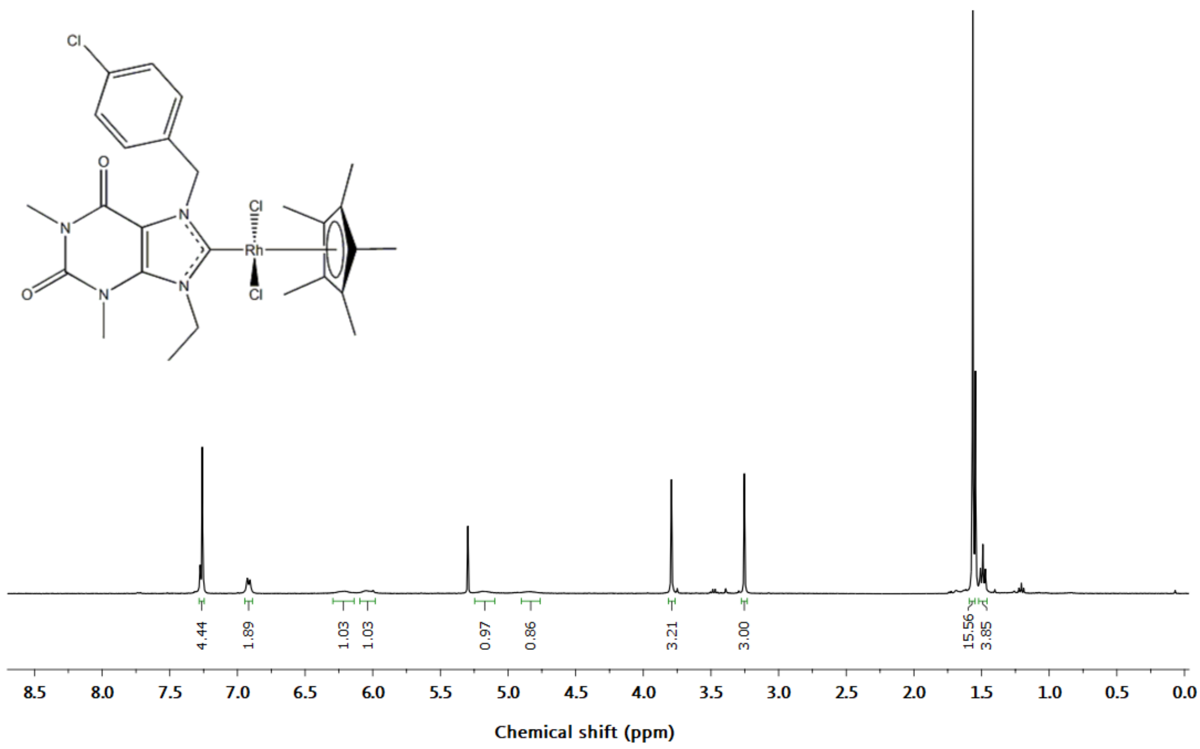

Figure S55

^{13}C-NMR, 150 MHz, $CDCl_3$

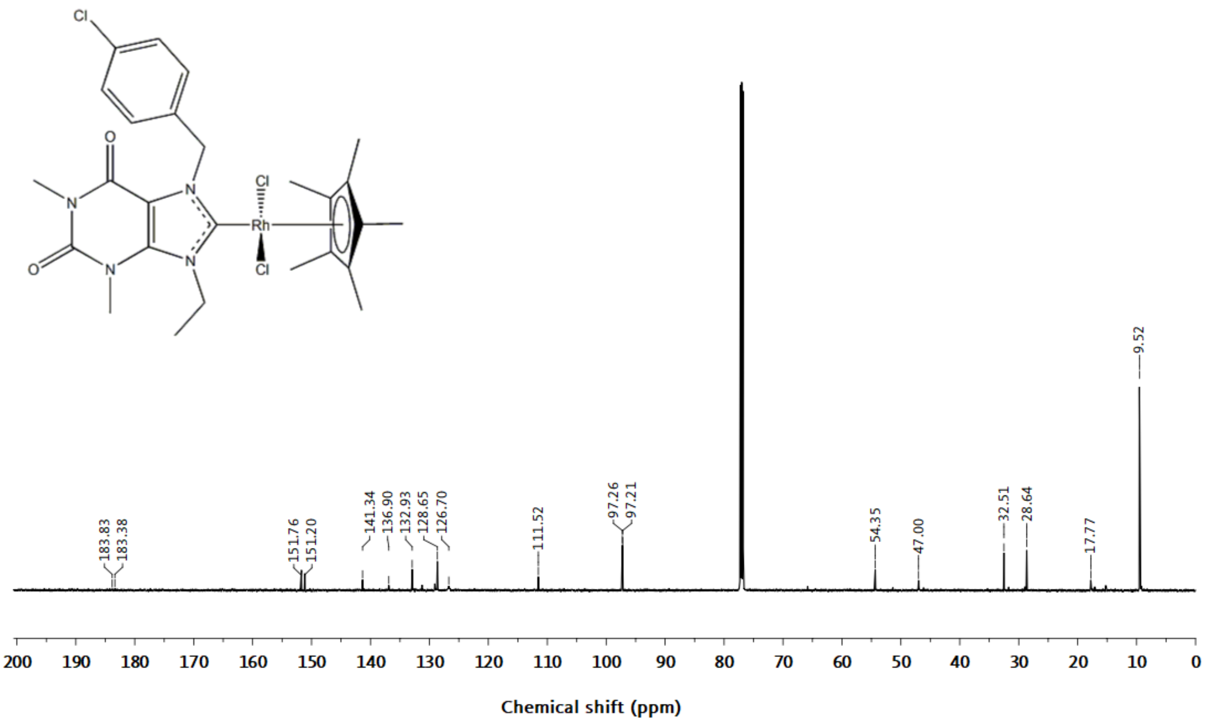

Figure S56

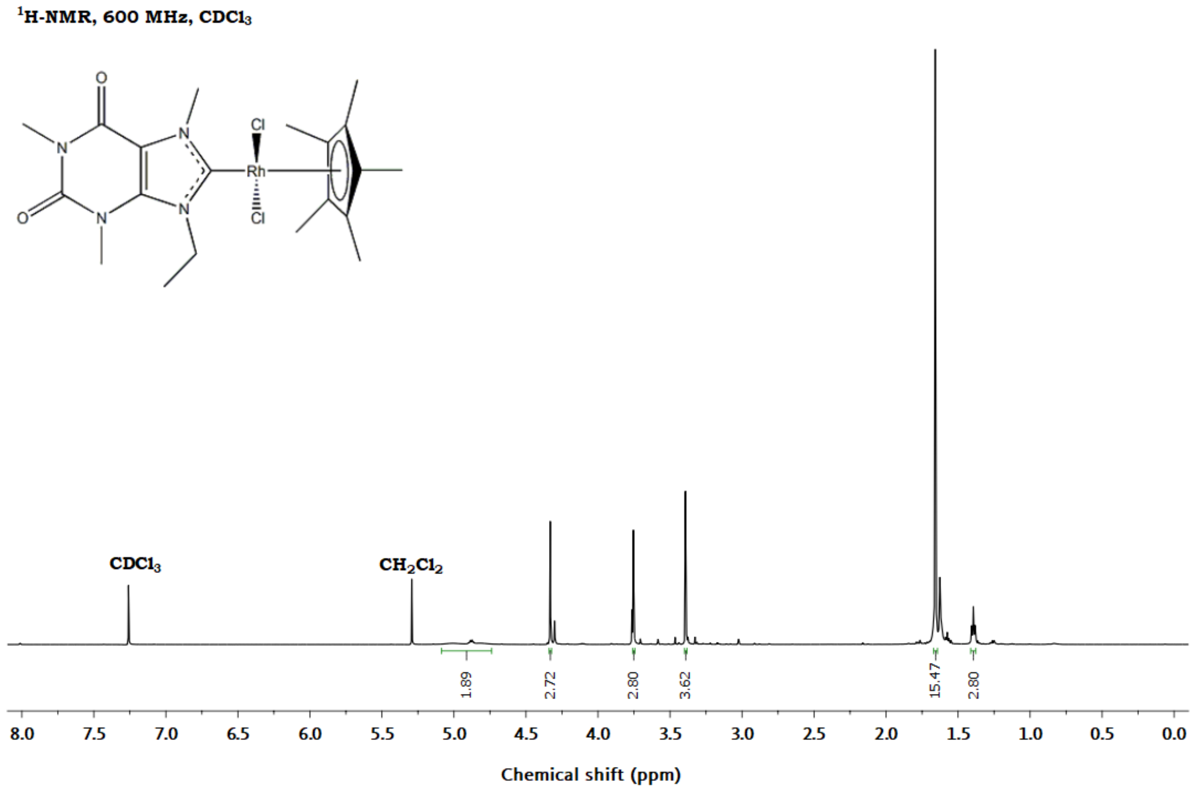

Figure S57

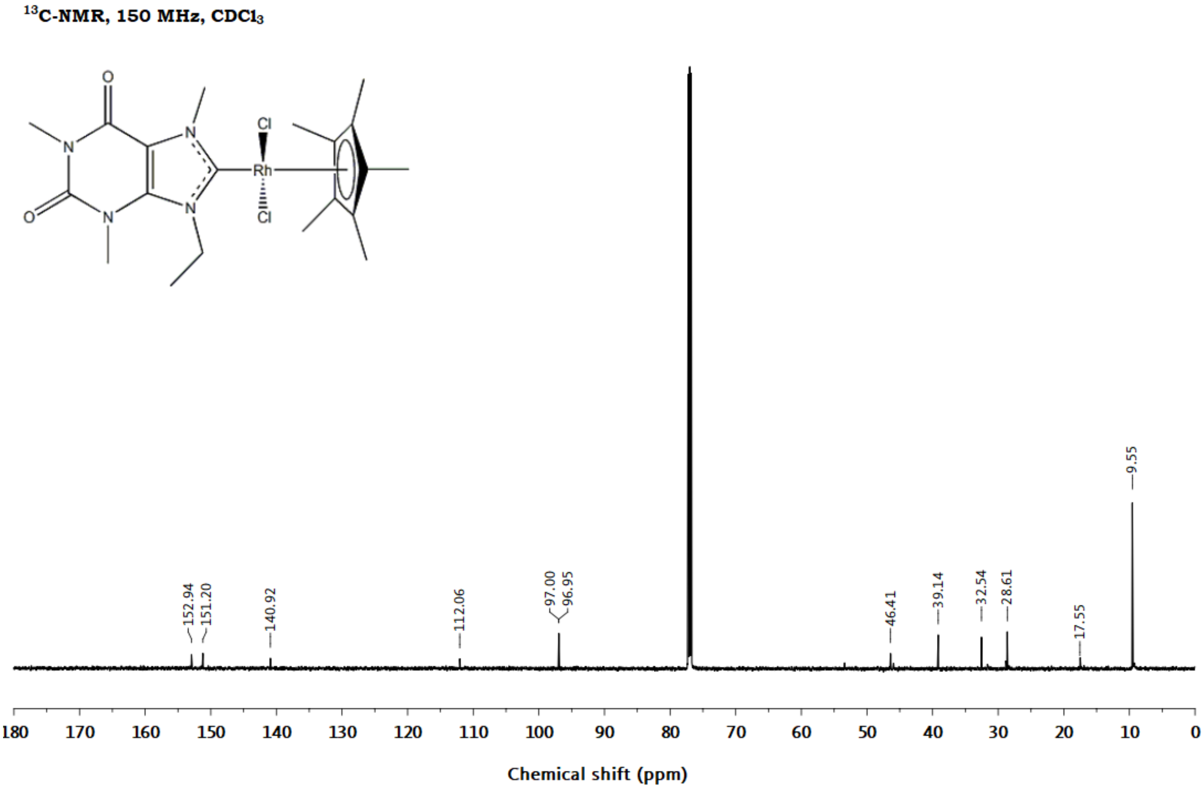

Figure S58

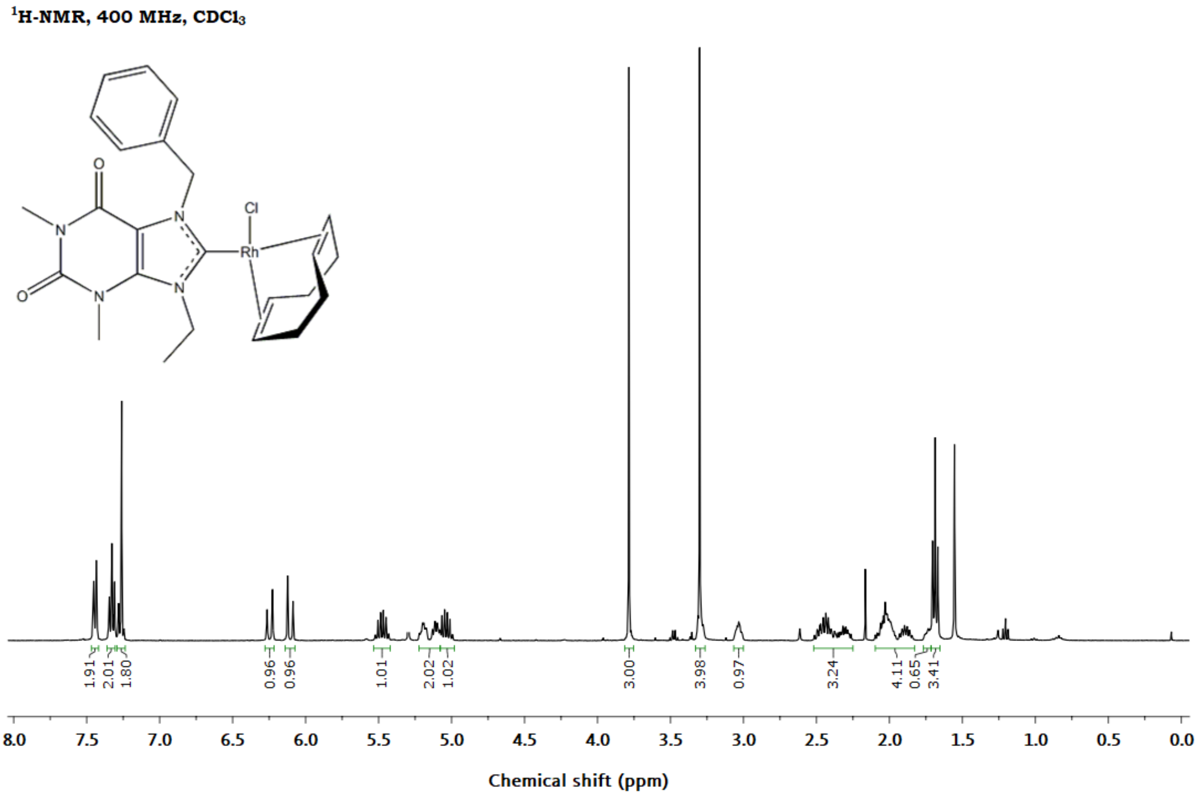

Figure S59

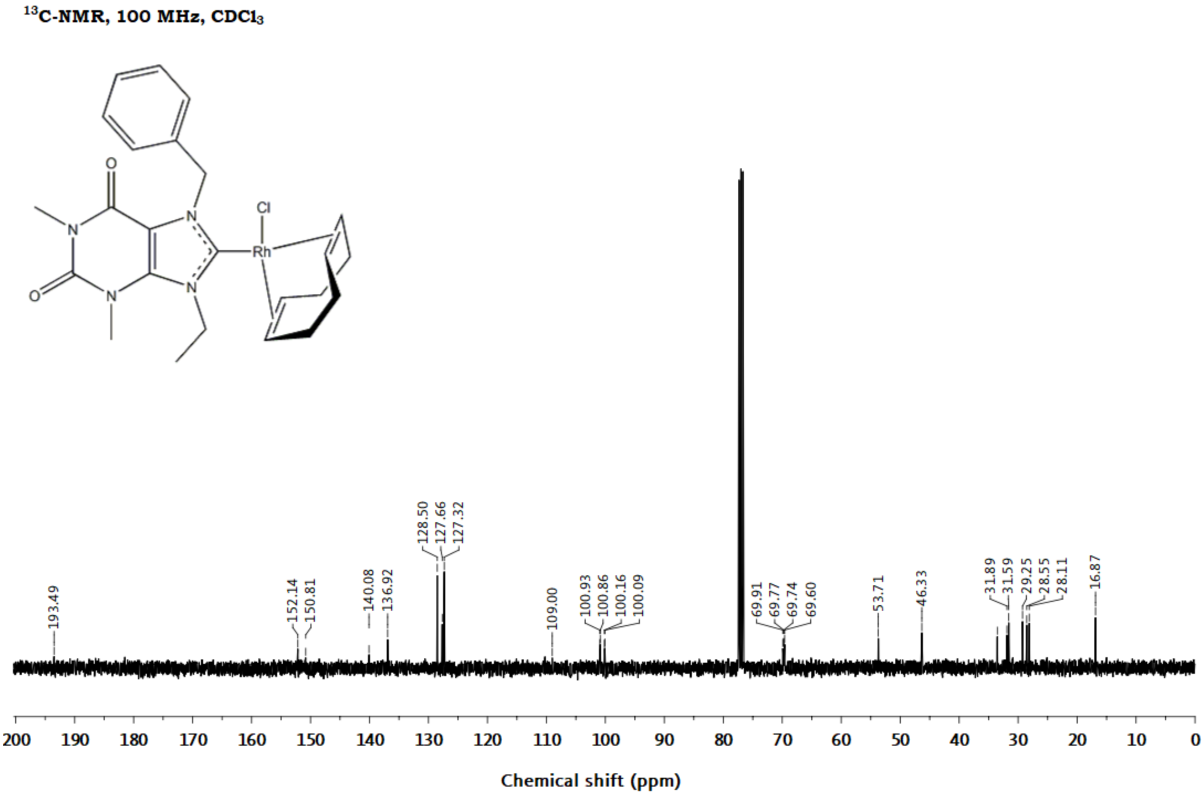

Figure S60

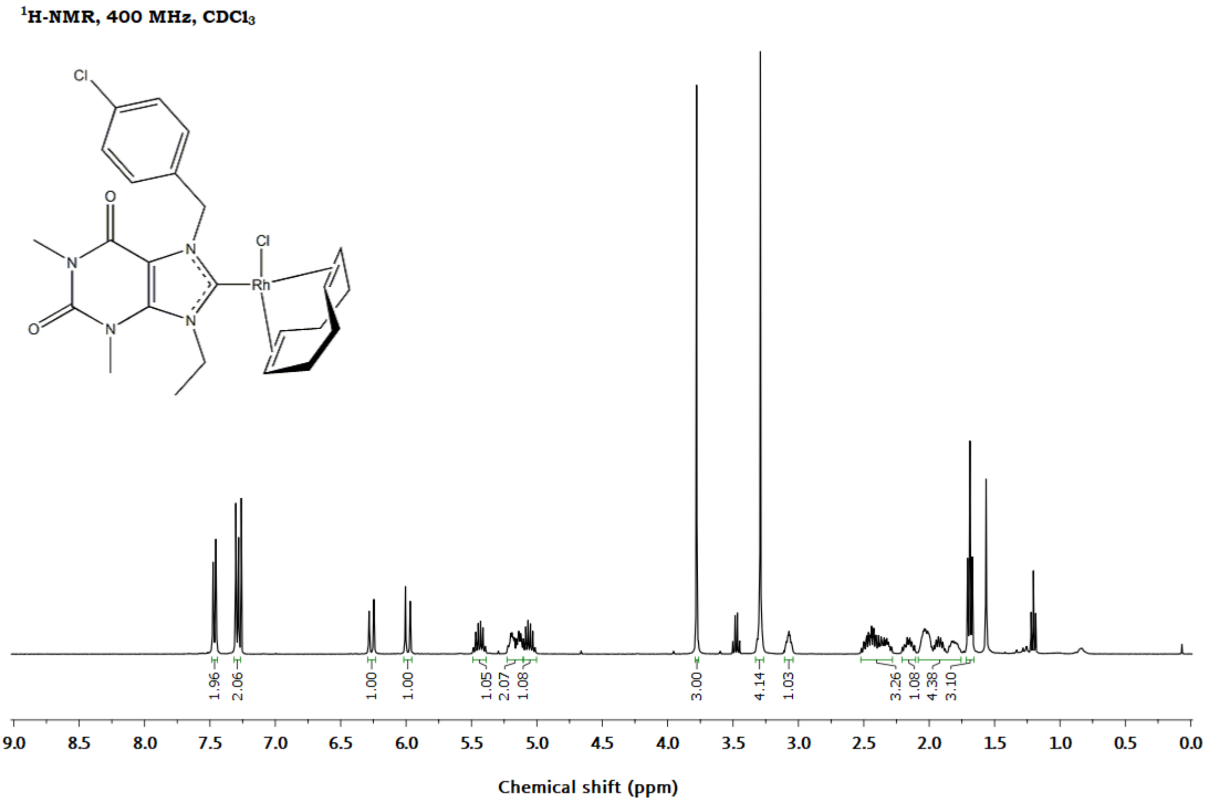

Figure S61

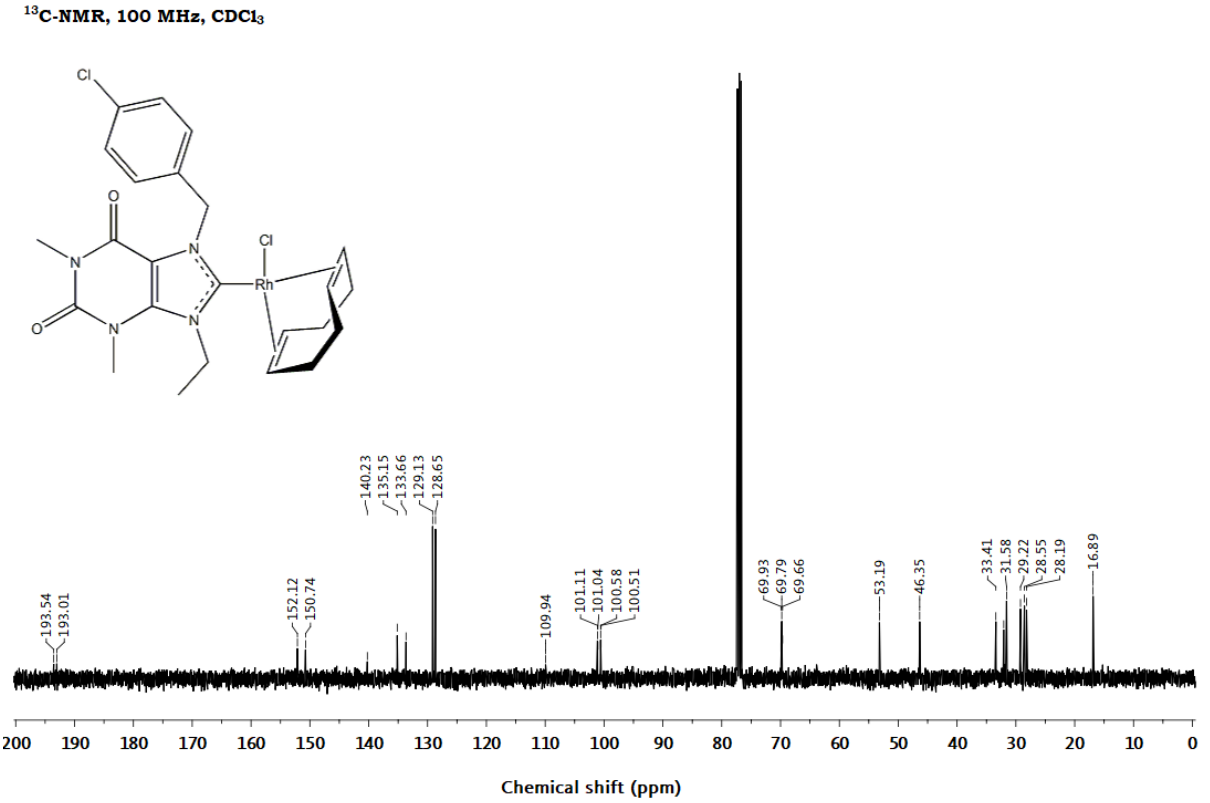

Figure S62

^{1}H-NMR, 400 MHz, $CDCl_3$

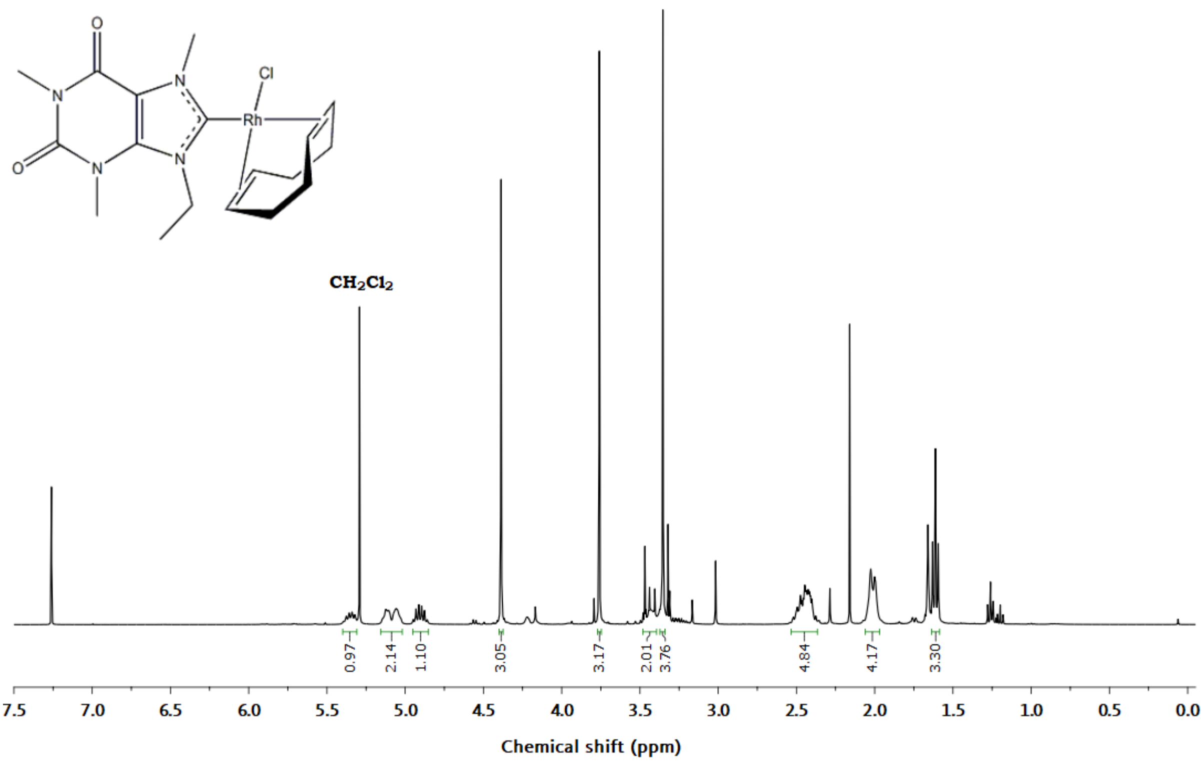

Figure S63

^{13}C-NMR, 150 MHz, $CDCl_3$

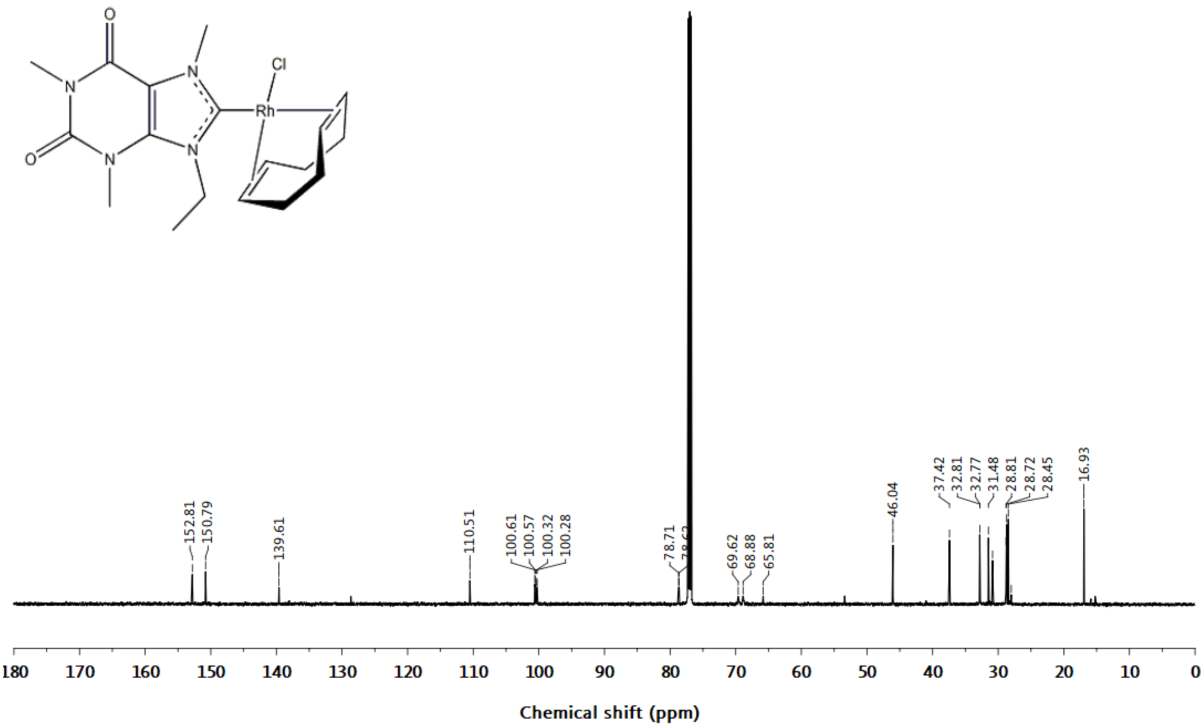

Figure S64

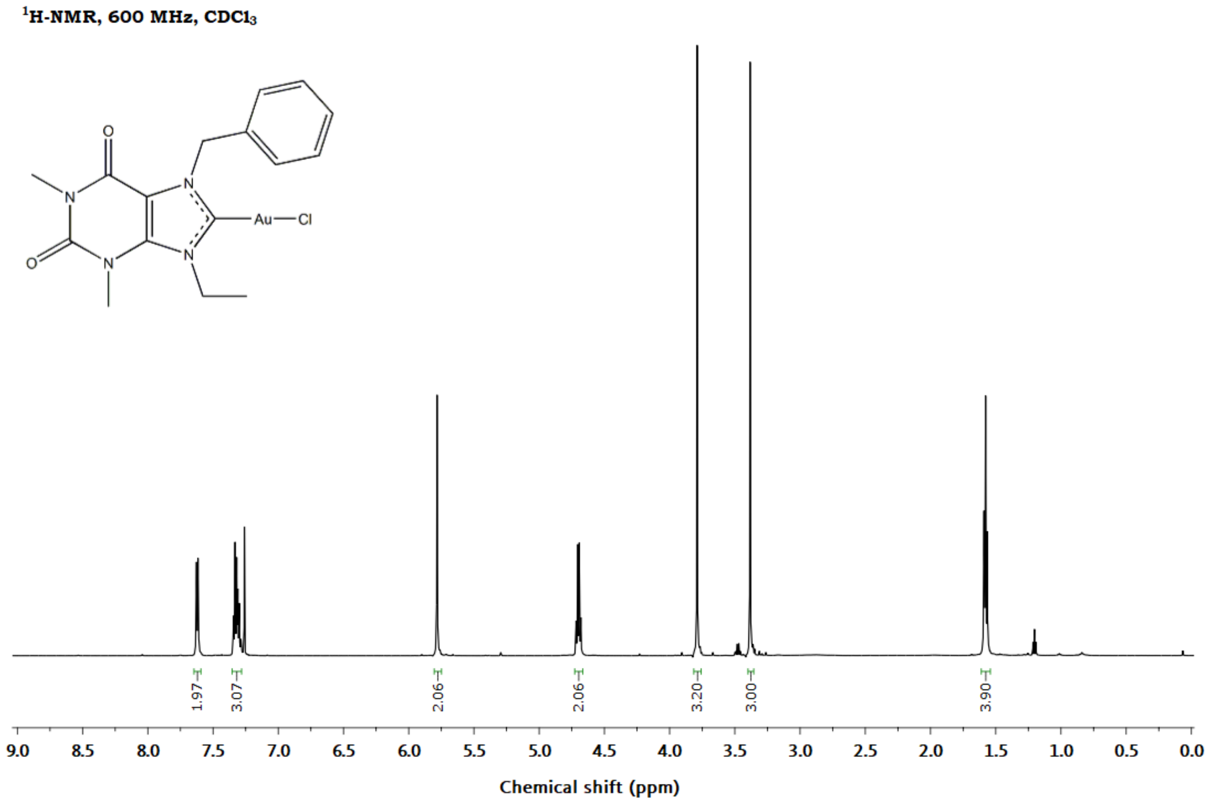

Figure S65

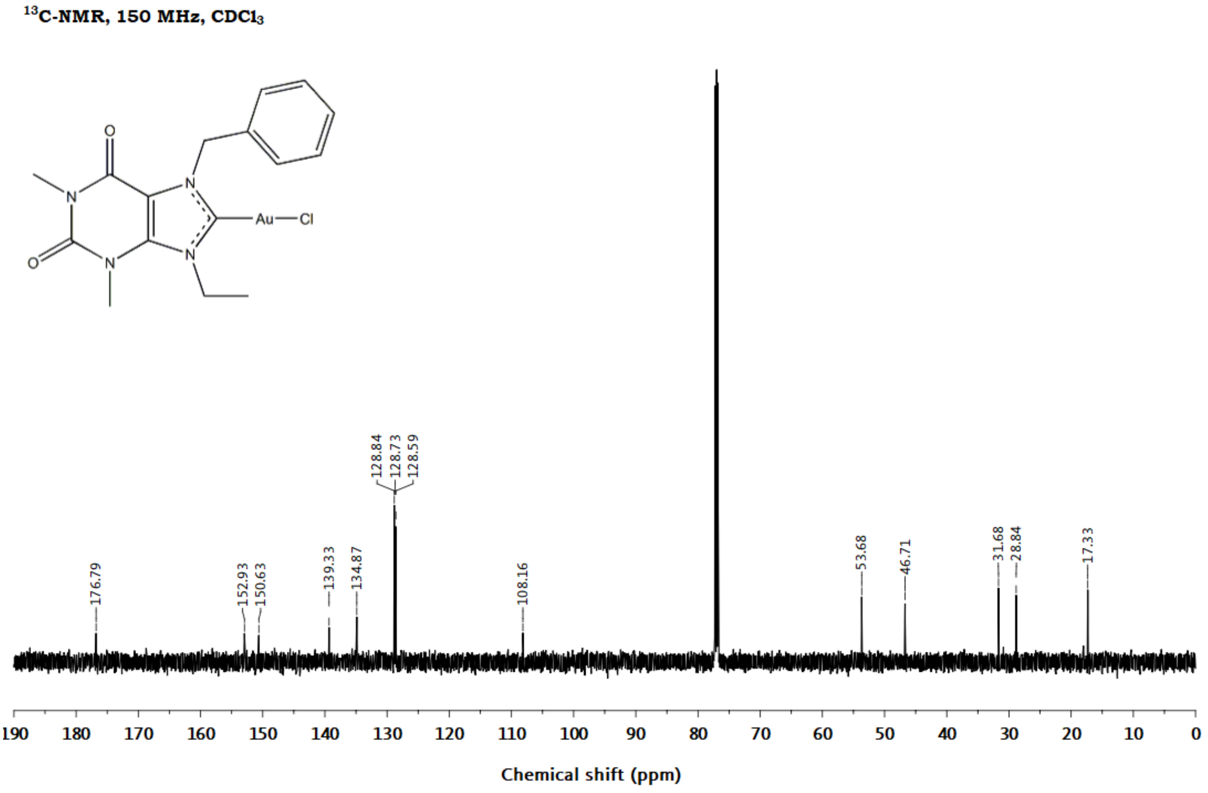

Figure S66

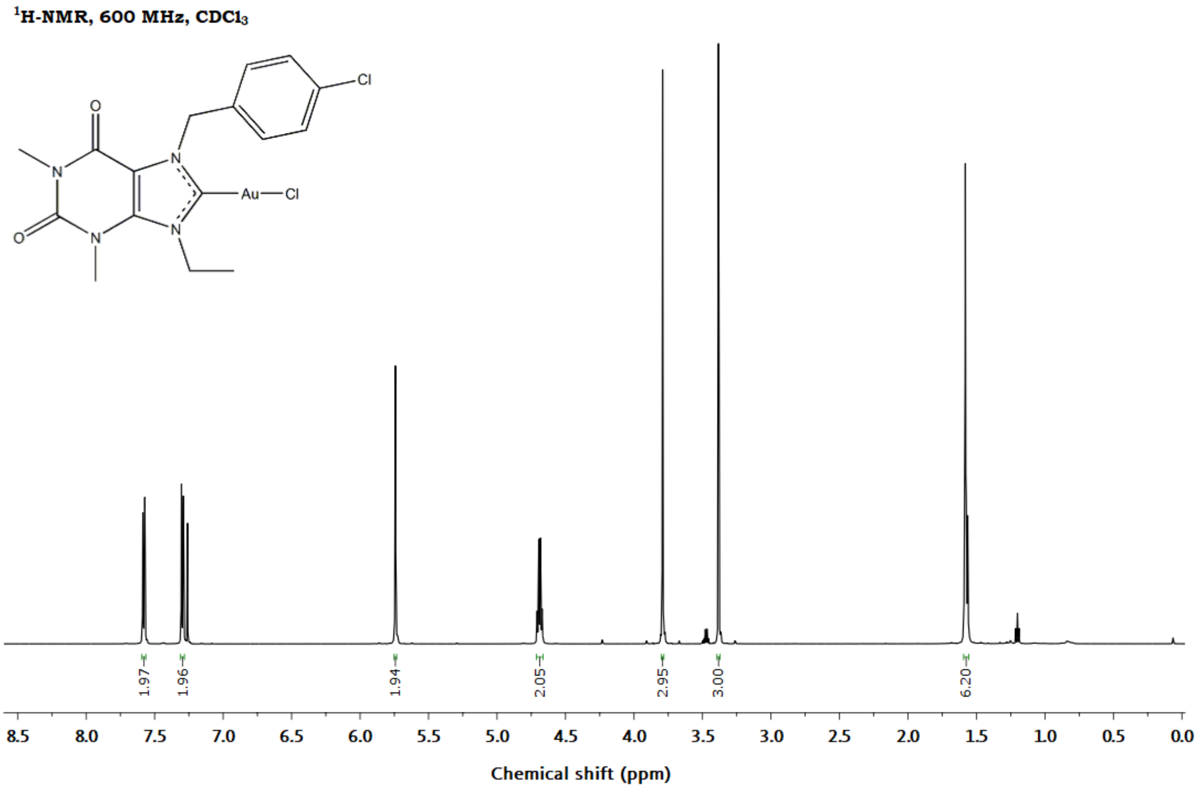

Figure S67

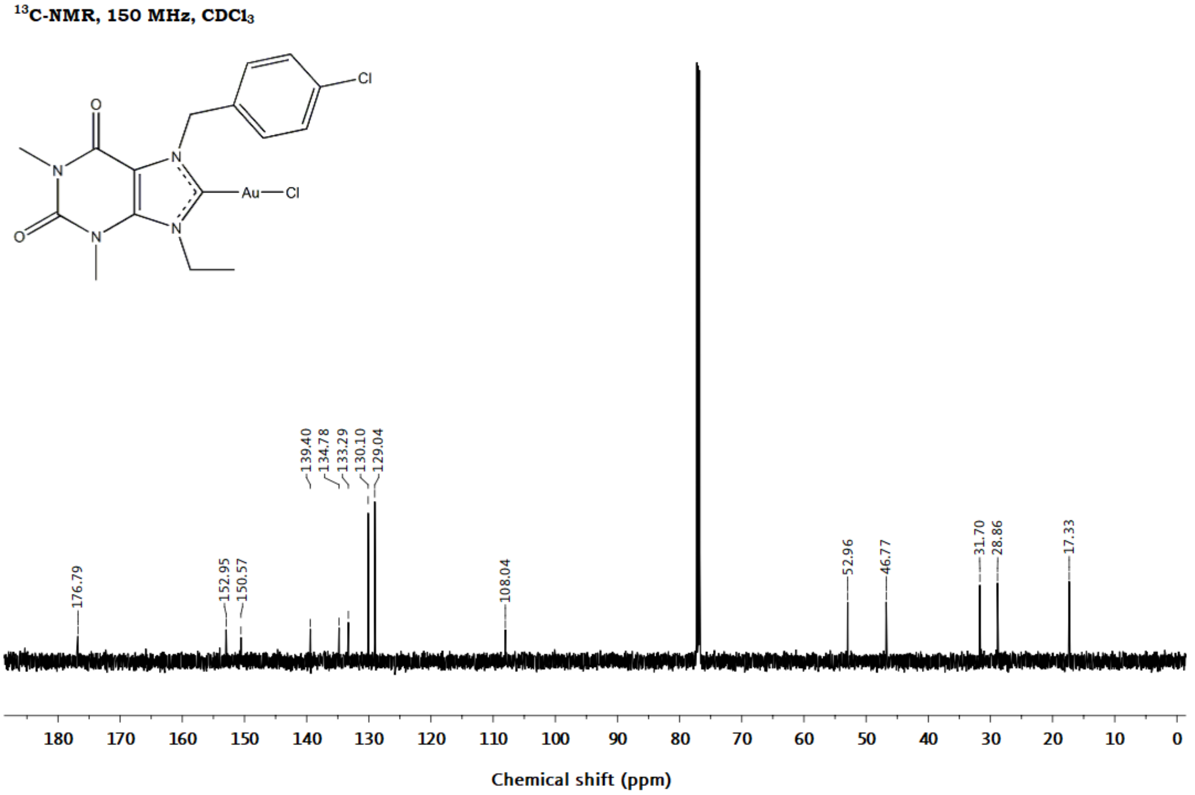

Figure S68

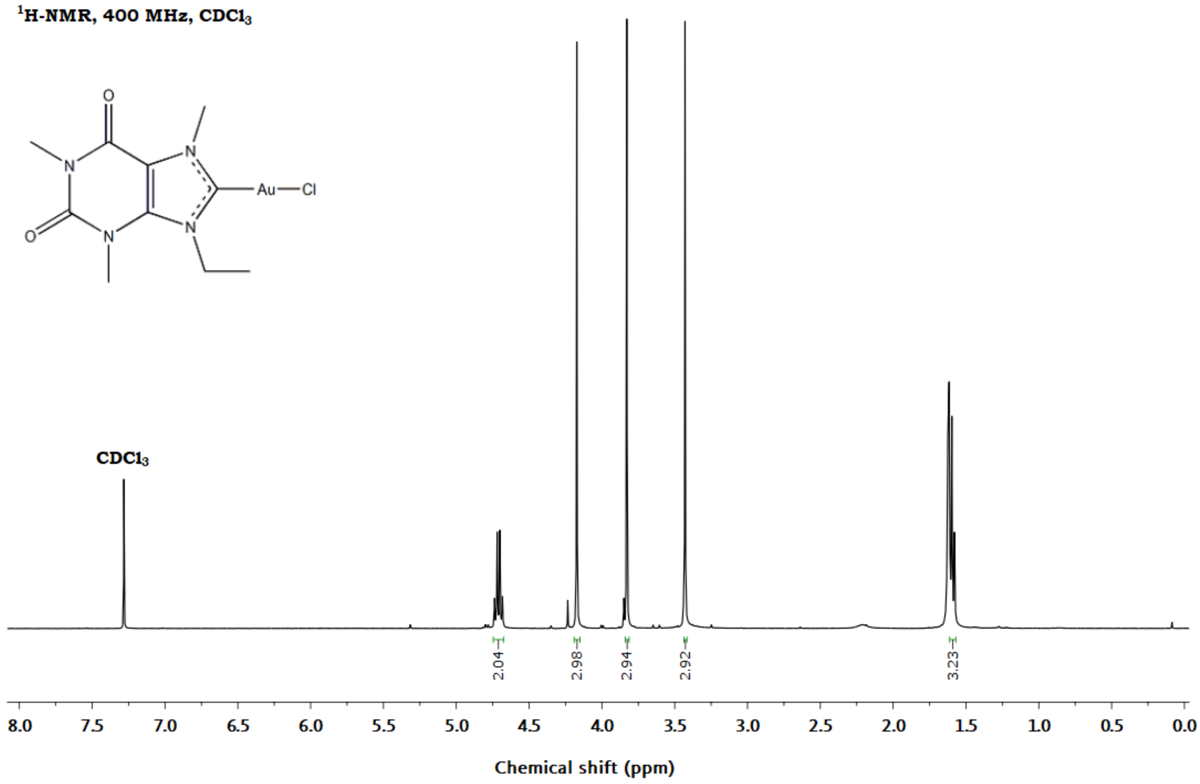

Figure S69

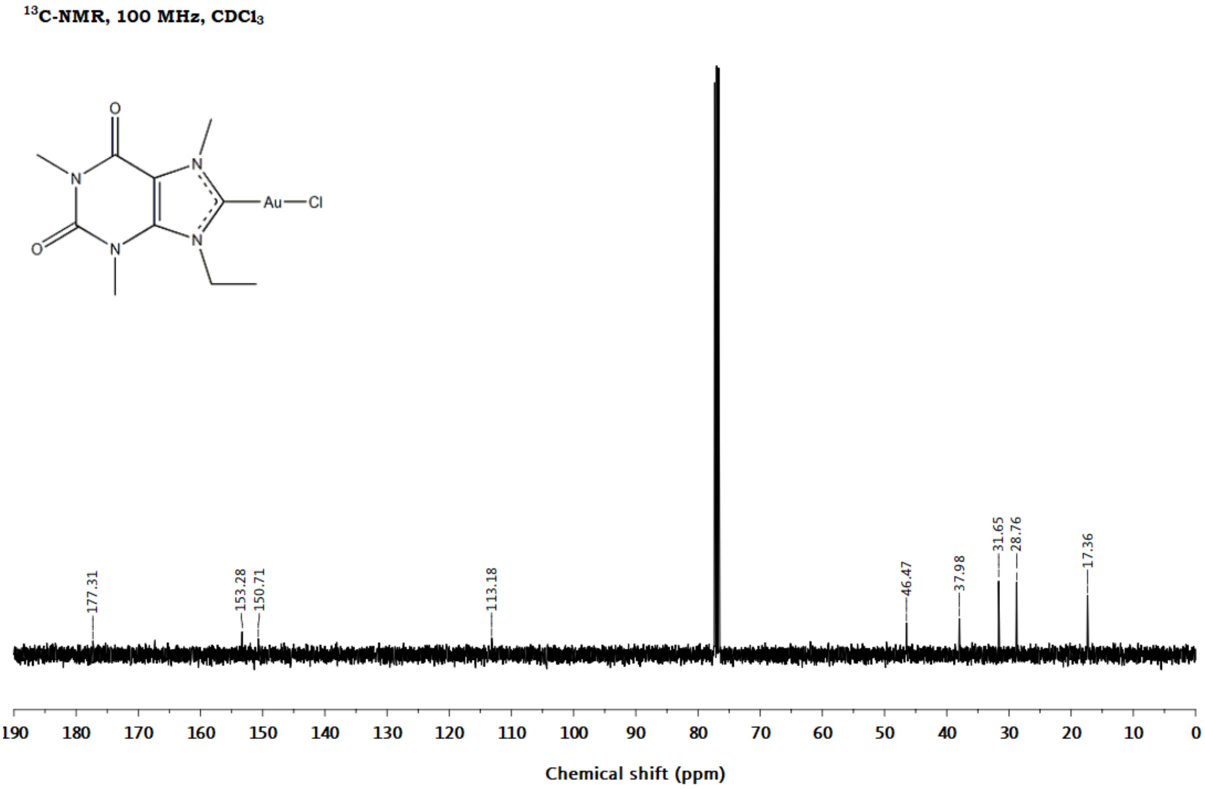

Figure S70
